# Achieving the first 90 for key populations in sub‐Saharan Africa through venue‐based outreach: challenges and opportunities for HIV prevention based on PLACE study findings from Malawi and Angola

**DOI:** 10.1002/jia2.25132

**Published:** 2018-07-22

**Authors:** Michael E Herce, William M Miller, Agatha Bula, Jessie K Edwards, Pedro Sapalalo, Kathryn E Lancaster, Innocent Mofolo, Maria Lúcia M Furtado, Sharon S Weir

**Affiliations:** ^1^ Department of Medicine UNC Institute for Global Health & Infectious Diseases University of North Carolina at Chapel Hill Chapel Hill NC USA; ^2^ UNC Project—Malawi Lilongwe Malawi; ^3^ Carolina Population Center University of North Carolina at Chapel Hill Chapel Hill NC USA; ^4^ Department of Epidemiology University of North Carolina at Chapel Hill Chapel Hill NC USA; ^5^ Tchikos Consultoría Cacuaco Angola; ^6^ Division of Epidemiology College of Public Health The Ohio State University Columbus OH USA; ^7^ Instituto Nacional de Luta contra a SIDA (INLS) Luanda Angola

**Keywords:** Key and vulnerable populations, HIV testing, HIV prevention, venue‐based outreach, hotspots, sub‐Saharan Africa, Malawi, Angola

## Abstract

**Introduction:**

Providing outreach HIV prevention services at venues (i.e. “hotspots”) where people meet new sex partners can decrease barriers to HIV testing services (HTS) for key populations (KP) in sub‐Saharan Africa (SSA). We offered venue‐based HTS as part of bio‐behavioural surveys conducted in urban Malawi and Angola to generate regional insights into KP programming gaps and identify opportunities to achieve the “first 90” for KP in SSA.

**Methods:**

From October 2016 to March 2017, we identified and verified 1054 venues in Luanda and Benguela, Angola and Zomba, Malawi and conducted bio‐behavioural surveys at 166 using the PLACE method. PLACE interviews community informants to systematically identify public venues where KP can be reached and conducts bio‐behavioural surveys at a stratified random sample of venues. We present survey results using summary statistics and multivariable modified Poisson regression modelling to examine associations between receipt of outreach worker‐delivered HIV/AIDS education and HTS uptake. We applied sampling weights to estimate numbers of HIV‐positive KP unaware of their status at venues.

**Results:**

We surveyed 959 female sex workers (FSW), 836 men who have sex with men (MSM), and 129 transgender women (TGW). An estimated 71% of HIV‐positive KP surveyed were not previously aware of their HIV status, receiving a new HIV diagnosis through PLACE venue‐based HTS. If venue‐based HTS were implemented at all venues, 2022 HIV‐positive KP (95% CI: 1649 to 2477) who do not know their status could be reached, including 1666 FSW (95% CI: 1397 to 1987), 274 MSM (95% CI: 160 to 374), and 82 TG (95% CI: 20 to 197). In multivariable analyses, FSW, MSM, and TGW who received outreach worker‐delivered HIV/AIDS education were 3.15 (95% CI: 1.99 to 5.01), 3.12 (95% CI: 2.17 to 4.48), and 1.80 (95% CI: 0.67 to 4.87) times as likely, respectively, as those who did not to have undergone HTS within the last six months. Among verified venues, <=68% offered any on‐site HIV prevention services.

**Conclusions:**

Availability of HTS and other HIV prevention services was limited at venues. HIV prevention can be delivered at venues, which can increase HTS uptake and HIV diagnosis among individuals not previously aware of their status. Delivering venue‐based HTS may represent an effective strategy to reach the “first 90” for KP in SSA.

## 1 Introduction

Key populations (KP) face a disproportionate HIV burden in sub‐Saharan Africa (SSA) 1. Driven by stigma, discrimination, limited KP‐friendly services, and other structural barriers, the HIV prevention, treatment, and care continuum remains inaccessible for many KP in the region 2, 3. As a result, progress toward ensuring universal access to HIV services for KP, and achieving HIV epidemic control, has been sub‐optimal in many SSA countries 1, 4.

Although distinct, the HIV epidemics in Malawi and Angola share features reflective of broader regional trends. In Malawi, the most recent evidence, from 2011 to 2014, indicates that HIV prevalence among the country's estimated 14,505 female sex workers (FSW) 5 is 62% to 69% 5, 6, and HIV prevalence among the estimated 38,734 men who have sex with men (MSM) 7, 8 is 18% 9—each substantially higher than the 9.2% prevalence in the 2016 general population 10. In Angola, while no national KP size estimates have been published, recent data suggest HIV prevalence of 10.5% among FSW in 2016 11 and 3.7% among MSM in 2011 12—both several times higher than the 2016 adult prevalence of 2.0%^13^. In both countries, data for transgender women (TGW) is virtually non‐existent, with one report, presenting 2011 to 2012 data, suggesting HIV prevalence among Malawian TGW may be 16% 14.

Across Malawi and Angola, important geographical variations exist in which urban areas report higher HIV prevalence than rural ones. National HIV responses have increasingly focused on reaching populations in urban locales, including KP, to achieve ambitious UNAIDS 90‐90‐90 targets 15. Realizing the “first 90” such that 90% of persons living with HIV (PLHIV), including KP, know their HIV status is important both for enabling PLHIV to start anti‐retroviral therapy (ART) and for supporting HIV‐negative people to access HIV prevention technologies. As demonstrated by recent national surveys from Malawi and other SSA countries, the most glaring challenge for HIV epidemic control remains reaching PLHIV unaware of their HIV status 2, 16.

In SSA, national efforts to reach KP with HIV testing services (HTS) and other HIV prevention offerings have traditionally relied upon generalized, facility‐based approaches 17, 18, 19. Such approaches have not been tailored to the unique needs and preferences of KP nor sufficiently addressed the myriad barriers that make facility‐based services inaccessible for many KP 3, 20, 21, 22. While scarce data from SSA describe the uptake and HIV positivity of KP‐focused HTS delivered outside facilities 23, data from other regions suggest superior HTS uptake by KP of community‐ over facility‐based approaches 24.

Since 2014, the PEPFAR‐funded LINKAGES project has partnered with KP communities throughout SSA, including in five southern African countries, Malawi and Angola among them, to improve KP access to HIV prevention, treatment, and care 25. Following WHO guidance, LINKAGES and other KP implementing partners have introduced programmes that involve KP as providers, such as KP peer educators and outreach workers, and deliver stigma‐free HIV services closer to KP communities 4, 26, 27, 28, 29, 30, 31. Emerging evidence suggests KP programmes incorporating such outreach activities may hold promise for engaging HIV‐negative KP to prevent HIV acquisition and for accelerating HIV diagnosis and linkage to care for HIV‐positive KP 32. When KP outreach services include venues where people meet new sex partners (i.e. “hotspots”), there may be additional benefits, such as reaching other populations at risk for HIV 33.

To address pressing gaps along the HIV cascade for KP, two LINKAGES‐supported countries in southern Africa—Malawi and Angola—requested PLACE (Priorities for Local AIDS Control Efforts) studies to inform LINKAGES programming. Using PLACE bio‐behavioural survey data from each country, we aimed to identify outreach strategies to accelerate progress towards the first 90 for KP and to generate new, regionally relevant insights into barriers to HIV prevention faced by KP. In this report, we highlight unmet KP programming needs and opportunities for improved HIV diagnosis and KP engagement in urban outreach settings in Malawi and Angola.

## 2 Methods

### 2.1 Ethics statement

The National Health Sciences Research Committee of Malawi (#15/7/1448), National Ethics Committee of the Ministry of Health of Angola, and University of North Carolina IRB (#15‐1903, #15‐1154) approved the study.

### 2.2 PLACE background

**Figure 1:**
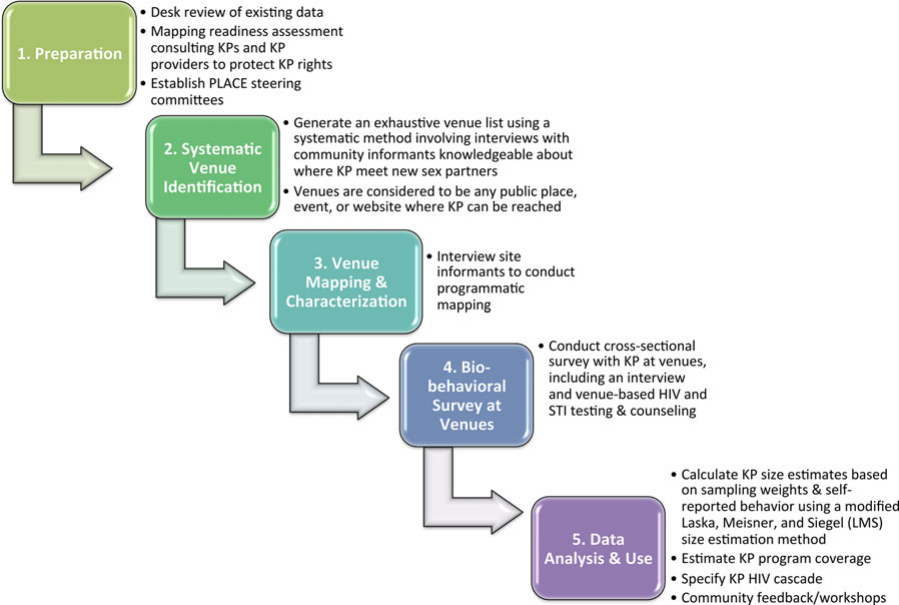
The five steps required for implementing the full PLACE protocol.

PLACE is a research methodology used at the local level to identify where to reach people most likely to acquire and transmit HIV and to assess programming coverage and gaps among those persons. PLACE can be used to estimate key population size, as well as HIV prevalence and HIV cascade indicators for KP and other at‐risk populations. PLACE methodology has been described extensively 33, 34, 35, 36, and encompasses 5 steps (Figure 1). Programmatically relevant insights can be gained from steps 1 to 3 plus step 5 (data analysis); full PLACE requires completing all 5 steps, including the bio‐behavioural survey (step 4). While PLACE is action‐oriented and involves KP in implementation, the protocol is designed to produce findings about KP programming gaps and HIV diagnostic yield that are more reflective of the local epidemiology and on‐the‐ground challenges in outreach settings than specific to the method itself.

### 2.3 PLACE geographical areas

In Malawi, six districts were selected for PLACE in response to national stakeholder guidance. One of these, Zomba, was selected for full PLACE based on implementing partner requests to identify pressing gaps along the HIV prevention‐to‐care continuum amenable to KP programming. In Angola, full PLACE data was available from two urban locales, Luanda and Benguela, selected using similar rationale 12, 37.

### 2.4 PLACE protocol overview

We implemented full PLACE concurrently in Malawi and Angola between October 2016—March 2017 as part of LINKAGES 38, using the same protocol adapted to each country's local context. Guided by PLACE steps (Figure 1), we first consulted local KP stakeholders through a mapping readiness assessment 39. Second, field teams systematically identified venues where KP could be reached and where people meet new sex partners through surveys with knowledgeable community informants. Venues included bars, clubs, motels/rest houses, brothels, festivals, and other publically accessible places and events. Third, field teams visited and verified these venues and surveyed site informants to assess HIV prevention service availability. Fourth, trained interviewers and social mobilizers from KP groups returned to a sample of venues to administer the bio‐behavioural survey to patrons and workers without regard to their KP status. We defined “patrons” as individuals socializing at, and “workers” as people employed by, the venue; either could include KP. Further details about PLACE are provided in the accompanying supporting information (Additional File 1).

### 2.5 PLACE participants

For step 3, site informants must have been: (1) knowledgeable about the venue; (2) ≥18 years old; and (3) willing to provide informed consent.

For step 4, interviewers first asked patrons and workers several screening questions designed to: be feasible in outreach settings; include all KP and other at‐risk populations based on less intrusive, general behavioural questions; avoid stigmatizing KP in public venues; and exclude people who would rarely classify as KP (Table 1). Screening questions were used to identify *potential* KP members only, and not to define participants as members of specific KP for the analysis. A random sample of individuals answering “yes” to ≥1 screening question were invited to participate in the bio‐behavioural survey, which included HTS. If the interviewers identified individuals at the site known to be FSW, MSM, or TG, these individuals were also invited to participate. Participant eligibility criteria included people: (1) ≥15 years old (≥18 years in Malawi); (2) willing to provide written informed consent; (3) new to the study; and (4) not intoxicated. After data collection, when analysing participants’ responses to sensitive questions exploring established KP behavioural factors, we applied internationally agreed upon definitions post hoc to assign a mutually exclusive KP classification of FSW, MSM, or TGW (Table 2) 4, 40, 41. For our FSW definition, we included females who received money for sex in the last six months and those who identified as FSW since both could be reached at venues with HIV prevention services and both reported similar demographic and behavioural characteristics (Additional File 2).

**Table 1:** Screening questions for PLACE bio‐behavioural survey

Country	Population	Behavioural factors assessed
Angola	Men/TGW	In the past six months, have you: Had anal sex with someone? Met a new sex partner online?
	Women	In the past week, have you had: >2 total sex partners?
Malawi	Men/TGW & Women	In the past three months, have you had: >5 total sex partners? Anal sex with anyone? Sex with someone met online or on a phone app? Missed taking prescribed medicine for a STI, such as HIV?

The following screening questions were used in each country to identify individuals to randomly select for participation in the PLACE bio‐behavioural survey; individuals answering “yes” to ≥1 questions passed the screen.

TG, Transgender women; STI, Sexually transmitted infection; HIV, Human immunodeficiency virus.

**Table 2:** Definitions for FSW, MSM, and TGW employed during PLACE data analysis

Population	Definition for Angola & Malawi
FSW	Assigned female sex at birth and received money for sex in the past six months or identified as a sex worker at time of survey.
MSM	Assigned male sex at birth and identified as male at time of survey; plus had anal sex with a man, paid a man for sex (in last six months), had ≥1 male partner (in last month), or identified as gay or bisexual at time of survey.
TGW	Assigned male sex at birth and identified with female gender or as transgender at time of survey.

Definitions based on behavioural factors identified post hoc through participants’ responses to questions in the bio‐behavioural survey.

FSW, Female sex worker; MSM, Men who have sex with men; TGW, Transgender women.

### 2.6 PLACE HTS and linkage to care procedures

All venues selected for full PLACE offered on‐site HTS, as part of the bio‐behavioural survey, at a discrete location selected in consultation with KP mobilizers and venue management, typically in a private room or project tent situated in a secluded area. All on‐site HTS was performed using HIV‐1/2 rapid antibody tests according to national guidelines 12, 42, and was conducted by trained counsellors who underwent regular proficiency testing for quality assurance purposes. Screening was performed with Determine HIV‐1/2 (Alere, Tokyo, Japan) followed by confirmatory testing with Uni‐gold HIV‐1/2 (Trinity Biotech, Bray, Co. Wicklow, Ireland). In cases of discordant or inconclusive results, repeat testing was done on a new sample: in Malawi, this involved repeat parallel testing and, in Angola, repeat serial testing 12, 42.

All bio‐behavioural survey participants provided voluntary informed consent for HTS separately. Study staff actively worked to link all participants with a new HIV diagnosis to care and treatment through phone and in‐person follow‐up and by connecting newly diagnosed individuals to peer health educators, where available. All participants received male condoms; no other reimbursement or incentive was provided.

### 2.7 PLACE data collection

For step 2, trained interviewers administered a community informant survey that asked participants to name and characterize sites where people meet new sex partners, and generated a de‐duplicated list of venues (Figure 1). For step 3, interviewers visited these venues to verify their existence and location, and to interview approximately one site informant per venue about site characteristics important for HIV prevention. Using this approach, we identified and verified a total of 1054 venues across Luanda, Benguela, and Zomba. Of these, field workers conducted bio‐behavioural surveys and offered HTS at 166 sites, including: 57 randomly and purposively selected sites in Zomba; 68 randomly chosen sites plus six events selected purposively in Luanda; and 31 randomly chosen sites plus four purposively selected events in Benguela. Survey data were collected using a tablet. De‐identified HIV test results were entered into Excel (Microsoft, Redmond, WA, USA). Survey and HIV testing data were subsequently linked through a unique identification number, and merged onto a secure database.

### 2.8 Data analyses

We present frequencies, percentages, and 95% confidence intervals (CI) for categorical variables. For all bio‐behavioural survey data, we weighted respondents according to their probability of being sampled following established methodology reported previously 43. Using this methodology, we assigned people selected randomly or through screening higher weights than those selected purposively. Each venue where participants were interviewed was assigned a venue weight based on the probability of venue selection for Steps 3 to 4. The final sampling weights combined the venue weights with each individual's probability of being selected for an interview 43. The estimated number of PLHIV unaware of their status at venues was calculated by applying the final sampling weights to the frequency of people with a positive HIV test but who did not self‐report being HIV‐positive, with CI calculations accounting for clustering by venue.

We estimated the association between outreach worker HIV prevention education and recent HIV testing, stratified by KP type, using bivariable log binomial regression and multivariable modified Poisson regression modelling and robust variance estimators. In our multivariable model, we controlled for age, country/locale and secondary education. We first conducted the analysis for each locale separately (data not shown). We then modelled results for each KP type, combining data from all locales, after demonstrating the homogeneity of the effect direction across the different geographic areas. All analyses were performed, using SAS 9.4 (Cary, NC, USA).

## 3 Results

### 3.1 Overview

We first present results for 959 FSW, 836 MSM, and 129 TGW surveyed across all locales. We then summarize results from 1054 site informant interviews to contextualize HIV prevention availability.

### 3.2 Demographic and behavioural characteristics

In Zomba, Malawi, FSW were mostly >25 years (55%), with 75% noting ≥1 new sex partner in the past four weeks. MSM and TGW were young with 76% and 82% <25 years, respectively. Approximately, 72% and 75% of MSM and TG, respectively, reported ≥1 new sex partner in the past four weeks.

In Angola, 55% of FSW were >25 years, and 89% had ≥1 new sex partner in the past four weeks. Angolan MSM and TGW were older than their Malawian counterparts with 59% and 67%, respectively, being >25 years. Approximately, 85% and 91% of MSM and TGW, respectively, reported ≥1 new sex partner in the past four weeks.

### 3.3 HIV prevalence and testing

HIV prevalence ranged from 2% (95% CI: 1% to 5%) to 62% (95% CI: 54% to 72%) depending on KP group and locale (Table 3). In Zomba, most FSW, MSM, and TGW had received HTS within the last six months, whereas in Angola, <25% of KP reported HTS in the past six months. Based on the weighted population prevalence, we estimated that 71% of KP living with HIV (KPLHIV) across all locales were not previously aware of their HIV status and received a new HIV diagnosis through PLACE venue‐based HTS. The proportion of KPLHIV newly HIV diagnosed through venue‐based HTS was highest among TGW, ranging from 23% (Zomba) to 100% (Benguela). We estimate that a combined 2022 HIV‐positive KP (95% CI: 1649 to 2477) who currently do not know their status could be newly diagnosed via venue‐based HTS should HTS be taken to scale in all study locales, including 1666 FSW (95% CI: 1397 to 1987), 274 MSM (95% CI: 160 to 374), and 82 TGW (95% CI: 20 to 197).

**Table 3:** HIV prevalence and HIV testing practices among KP who socialize at venues

	Total Sample^a^			Population Prevalence^b^								
	FSW (N = 954)	MSM (N = 832)	TGW (N = 126)	Zomba, Malawi^a^			Luanda, Angola			Benguela, Angola		
	n	n	n	FSW n = 106	MSM n = 119	TGW n = 53	FSW n = 505	MSM n = 457	TGW n = 46	FSW n = 343	MSM n = 256	TGW n = 27
Total HIV‐positive	118	22	9	62%	2%	20%	8%	2%	9%	5%	3%	6%
Self‐Reported	43	4	2	60%	50%	77%	20%	28%	57%	4%	7%	0%
Newly diagn‐osed by Venue‐based Outreach HTS	75	18	7	40%	50%	23%	80%	72%	43%	96%	93%	100%
Ever tested for HIV and received test results	550	393	80	88%	75%	72%	53%	47%	65%	58%	36%	41%
In the last six months, tested for HIV and received test results	187	164	50	75%	54%	65%	16%	17%	24%	9%	6%	21%

FSW, Female sex worker; MSM, Men who have sex with men; TGW, Transgender women; HTS, HIV testing services; HIV, human immunodeficiency virus.

a 5 FSW, 4 MSM and 3 TGW did not test for HIV or had invalid results.

b Population prevalence is weighted based on venue‐based sampling strategy.

### 3.4 Access to basic HIV prevention

KP respondents frequently reported recent condomless penile‐vaginal and anal sex (Table 4). Despite the prevalence of high‐risk sex, few respondents reported having a condom on their person or recently obtaining free lubricant. Similarly, in Zomba and Luanda, ≤50% of all participants reported receiving health information on site from an outreach worker. In Benguela, availability of this service was only slightly more common.

**Table 4:** Unmet needs for basic HIV prevention services among KP who socialize at venues

Unmet Needs	Total Sample			Population Prevalence^a^								
	FSW (N = 959)	MSM (N = 836)	TGW (N = 129)	Zomba, Malawi			Luanda, Angola			Benguela, Angola		
	n	n	n	FSW n = 111	MSM n = 123	TGW n = 56	FSW n = 505	MSM n = 457	TGW n = 46	FSW n = 343	MSM n = 256	TGW n = 27
Recent penile‐vaginal sex^b^	915	645	60	88%	77%	72%	95%	72%	17%	100%	95%	59%
Recent condom‐less penile‐vaginal sex^b^	655	490	44	67%	62%	52%	73%	79%	91%	70%	71%	86%
Recent anal sex with a man^b^	383	756	117	19%	75%	79%	57%	94%	94%	23%	89%	67%
Recent anal sex without a condom^b^	273	455	69	98%	70%	72%	71%	55%	46%	62%	67%	56%
Has condom on person at time of interview	263	237	42	46%	18%	19%	33%	42%	56%	12%	14%	16%
Obtained free lubricant in the past six months	155	163	36	34%	15%	10%	21%	24%	34%	5%	15%	18%
Received information about HIV/AIDS from outreach worker at venue in last 12 months	428	298	46	50%	23%	31%	36%	30%	31%	60%	52%	57%

FSW, Female sex worker; MSM, Men who have sex with men; TGW, Transgender women; HIV, human immunodeficiency virus; AIDS, Acquired immune deficiency syndrome.

a Population prevalence is weighted based on venue‐based sampling strategy.

b ”Recent” refers to a period within three months of survey administration in Malawi, and six months of survey administration in Angola.

Across locales, receiving HIV/AIDS information from an outreach worker was significantly associated with having undergone HIV testing in the past six months for FSW and MSM, but not for TGW (Table 5). The association observed among FSW and MSM remained statistically significant after controlling for age, education, and country/locale. In the multivariable model, FSW, MSM, and TG who received HIV/AIDS education from an outreach worker were 3.15 (95% CI: 1.99 to 5.01), 3.12 (95% CI: 2.17 to 4.48), and 1.80 (95% CI: 0.67 to 4.87) times as likely, respectively, as those who did not to have undergone HTS within the last six months.

**Table 5:** Estimated effects of HIV/AIDS education delivered by outreach workers on HIV testing uptake within the past six months

Participant Type	Received information about HIV/AIDS from an outreach worker at the venue in last 12 months?	Unadjusted		Adjusted^a^	
		PR 95% CI	*p*‐value	PR 95% CI	*p*‐value
FSW	Yes	3.23 (2.01, 5.17)	<0.01	3.15 (1.99, 5.01)	<0.01
	No	1.00		1.00	
MSM	Yes	3.05 (2.10, 4.43)	<0.01	3.12 (2.17, 4.48)	<0.01
	No	1.00		1.00	
TGW	Yes	0.80 (0.18, 3.58)	0.78	1.80 (0.67, 4.87)	0.25
	No	1.00		1.00	

PR, Prevalence ratio; CI, Confidence interval; FSW, Female sex worker; MSM, Men who have sex with men; TGW: Transgender women.

a Adjusted for age, country/locale, and education level.

### 3.5 Access to other prevention

A clinically meaningful proportion of respondents reported a recent genital sore (Table 6). Despite this, <50% of participants reported having undergone a STI evaluation by a medical provider within the last year.

**Table 6:** Opportunities for outreach screening and treatment for STIs and TB per KP who socialize at venues

Symptom/Service	Total Sample			Population Prevalence^a^								
	FSW (N = 959)	MSM (N = 836)	TGW (N = 129)	Zomba, Malawi			Luanda, Angola			Benguela, Angola		
	n	n	n	FSW n = 111	MSM n = 123	TGW n = 56	FSW n = 505	MSM n = 457	TGW n = 46	FSW n = 343	MSM n = 256	TGW n = 27
In past four weeks, had genital sore	118	119	16	13%	7%	13%	11%	13%	16%	12%	22%	10%
In past 12 months, examined/tested by a medical provider for STI (other than HIV)	251	235	30	36%	21%	27%	27%	28%	24%	25%	38%	34%
With symptoms compatible with possible TB (cough, fever, night sweats, weight loss)^b^	188	165	25	15%	18%	21%	21%	18%	38%	18%	24%	15%
In the past 12 months, provided a sputum sample for TB diagnostic test	37	57	7	9%	4%	10%	2%	4%	1%	4%	11%	0%

FSW, Female sex worker; MSM, Men who have sex with men; TGW, Transgender women; STI, Sexually transmitted infection; HIV, human immunodeficiency syndrome; TB, tuberculosis.

a Population prevalence is weighted based on venue‐based sampling strategy.

b Adapted from the World Health Organization 4‐symptom TB screen for persons living with HIV 44.

High symptom prevalence was also reported for tuberculosis (TB) (Table 6). Across locales, 15% to 38% of respondents endorsed ≥1 current TB symptom 44, but only 0% to 11% of participants reported providing a sputum sample for TB testing within the last year.

### 3.6 Outreach HIV prevention service availability

Across locales, venue‐based outreach services were infrequently available, with ≤68% of site informants reporting any on‐site HIV prevention service availability (Table 7). On‐site HTS and outreach worker‐led prevention education were uncommonly reported.

**Table 7:** Availability of any outreach HIV prevention services at venues per community site informants

	Zomba, Malawi % (n = 166)	Luanda, Angola % (n = 536)	Benguela, Angola % (n = 352)
Any HIV/AIDS prevention service available on site within the past six months?^a^	68%	57%	24%
Any on‐site HIV testing service within the past six months?	2%	6%	1%
Any availability of male condoms (free or for sale) in the past six months?	61%	51%	21%
Any availability of lubricant (free or for sale) in the past six months?	2%	27%	3%
Any safe sex education offered by an outreach worker within the past six months?	7%	12%	4%
Any visits by a mobile clinic within the past six months?	1%	3%	0%

HIV, human immunodeficiency virus; AIDS, Acquired immune deficiency syndrome.

a Any prevention” encompasses ≥1 of the following services: male condoms, lubricant, HIV testing, outreach/peer worker services, mobile clinic or needle exchange.

## 4 Discussion

We report a high proportion of KPLHIV who were previously unaware of their HIV status before being newly diagnosed through venue‐based HTS, including one of the first accounts of HIV prevalence and testing histories among TGW from Angola or Malawi. Despite the number of new HIV diagnoses made through PLACE, KP and site informants reported limited access to HTS and other venue‐based prevention services. We note that HTS can be delivered at venues, enabling new HIV diagnoses in individuals not previously aware of their HIV status. Venue‐based HTS may be particularly impactful for KP who socialize at venues, many of whom report not having received HTS in the past six months, but who may potentially be more likely to undergo HTS if they receive HIV/AIDS information from an outreach worker. Delivering outreach HTS alongside other HIV prevention services may represent an effective strategy to accelerate progress towards the first 90 for KP in SSA.

Our study demonstrated that more than 70% of HIV‐positive KP were not previously aware of their HIV status and received a new HIV diagnosis through venue‐based HTS. These new diagnoses were made even though most participants reported having previously tested for HIV. These data align with recent evidence from Malawi where relatively few MSM and FSW living with HIV were previously aware of their HIV status 6, 9, 45. We estimate that over 2000 KPLHIV who do not currently know their status could be newly diagnosed if venue‐based HTS were expanded to all venues in the studied locales. Providing HIV/AIDS information via an outreach strategy could help facilitate such scale up based on our finding that outreach worker‐led education was associated with increased recent HIV testing for FSW and MSM—an observation among the first of its kind from SSA. The fact that this association was not observed among TGW people warrants further study, and may reflect the scarcity of TGW‐tailored outreach services in SSA 14, 46.

Venue‐based HTS may also provide opportunities to serve other at‐risk populations unaware of their HIV status. For example, among 720 non‐MSM, cisgender men tested across study locales, 3% were found to be HIV positive, and of these, 76% were newly diagnosed. Similarly, HIV prevalence among all 380 non‐FSW female respondents was 2%, and 84% of these were newly diagnosed. These results suggest venue‐based HTS may be an underutilized strategy to improve testing coverage among men, single adults, and other populations not currently served by more traditional HTS approaches 47.

Beyond HTS, the high proportion of disclosed high‐risk behaviours, including condomless sex, suggest a large unmet need for HIV prevention services. Indeed, inconsistent condom use among MSM has previously been identified as a risk factor for prevalent HIV infection in Malawi 3. Despite the documented need and obvious public health importance, free condom and lubricant provision was infrequently reported at venues. Limited access to free condoms and condom‐compatible lubricant is particularly problematic for MSM and FSW who must overcome multiple structural barriers to purchase or carry these commodities 48, 49. It is not surprising, then, that prevalence of self‐reported recent genital sore was relatively high in our study, exceeding 10%. Such genital sores and STIs are easily amenable to syndromic management or point‐of‐care diagnosis and tailored treatment, either of which could be reliably provided in an outreach setting by a trained provider.

To improve access to these services, new approaches are urgently needed that involve KP leaders and serve as a bridge between communities and traditional service delivery platforms 32, 50. Hybrid models that link HIV prevention services provided by KP community groups with treatment offered through national ART programmes may be one such approach 30. In the hybrid model, outreach HTS can serve as an entry point to ART offered through government clinics or community‐based drop‐in‐centres. For such an approach to succeed, HTS entry points must be expanded and facilitated linkage to care strengthened 23, 51.

Given the preponderance of high‐risk behaviours, limited STI screening, and substantial HIV diagnostic yield reported here, our study provides evidence to support greater focus on delivering venue‐based outreach services in SSA. A basic service package could include: HTS, outreach HIV prevention education, free condoms and lubricant, STI and TB screening and treatment, and peer navigator support to help newly HIV‐diagnosed KP link to care and initiate ART 52, 53. Such a package echoes ongoing efforts to reach KP in SSA 27, 28, 32. While the cost‐effectiveness of such services requires further investigation, modelling data suggest that simply focusing HIV prevention interventions on the places and populations with highest risk could advert thousands of new infections without requiring additional resourcing 54.

Due to the cross‐sectional nature of our study, we could not infer causality nor evaluate the effects of venue‐based HTS, or other outreach services, on longitudinal HIV‐related outcomes, including successful linkage to care for newly HIV‐diagnosed KP. In addition, the low HIV prevalence identified among MSM in Zomba, compared to prior Malawi estimates 9, 55, raises the possibility that some sites where MSM socialize were missed, or that some PLHIV who already knew their status declined participation because HTS was a required study procedure. Finally, our pragmatic approach precluded us from carrying out more time‐intensive study procedures, such as in‐depth interviews, to fully assess KP attitudes and preferences regarding venue‐based HIV services.

For any service delivery model to succeed, KP constituency engagement and support for model design, implementation, and monitoring is essential 30, 50, as is involving public‐sector partners to ensure universally accessible HIV, STI, and TB prevention, treatment, and care. With greater resourcing, hybrid models providing venue‐based outreach services could expand to include mobile clinics employing trained health workers and peer navigators to provide a comprehensive suite of health services aligned with international normative guidance 4, 31.

## 5 Conclusions

If efforts to promote KP human rights and achieve epidemic control in SSA are to be realized, service providers must take advantage of all opportunities to expand access to HTS, and other HIV prevention services, for KP and other at‐risk groups. Venue‐based outreach may be one such opportunity to serve these populations at the sites where they socialize. While the capacity of many providers may be insufficient to offer a full HIV service delivery package in outreach settings, this need not be an excuse for inaction. Rather, offering on‐site HTS and a basic suite of HIV prevention services can be an important initial step towards reaching the “first 90” and increasing access to HIV services for those who need them most.

## Authors’ contributions

MEH, WMM and SSW had overall responsibility for implementing the study, and conceived and designed the study, analysed the data, and led the manuscript writing. MEH, WMM, AB, JKE, KEL, IM and SSW contributed to developing the study concept and design. AB, PS, WMM and SSW contributed to data collection. WMM, JEL and SSW assisted with data analysis and results interpretation. MEH, WM and SSW contributed to drafting the manuscript. All authors reviewed the manuscript critically for intellectual content. All authors read and approved the final draft of the submitted manuscript.

## Competing interests

The authors declare that they have no competing interests.

## Supporting information


**Additional file 1**: Angola PLACE protocol

**Additional file 2**: Table S1. Demographic and behavioural characteristics of women identifying as FSWs and women reporting receiving money for sex in the last six months.
