# Supplementary material for: Achieving the first 90 for key populations in sub‐Saharan Africa through venue‐based outreach: challenges and opportunities for HIV prevention based on PLACE study findings from Malawi and Angola
Source: J Int AIDS Soc. 2018 Jul 22;21(Suppl Suppl 5):e25132. doi: 10.1002/jia2.25132 (PMC6055127; doi:10.1002/jia2.25132)
Supplement: Supplementary file 1 — Additional file 1: Angola PLACE protocol [file JIA2-21-e25132-s001.pdf]

# Mapping Readiness Assessment, Programmatic Mapping and Biobehavioral Surveillance among Key Populations in Angola

**Principal Investigator:**

Dra. Maria Lucia Mendes Furtado, Instituto Nacional de Luta contra à SIDA (INLS)

**Co-Investigators:**

Dr. Marquez Gomez, Instituto Nacional de Luta contra à SIDA (INLS)
Pedro Sapalalo, Tchikos Consultoria Empresarial
Michael Herce, Projecto LINKAGES/USAID
William Miller, Projecto LINKAGES/USAID
Kate Muessig, Projecto LINKAGES/USAID
Sharon Weir, Projecto LINKAGES/USAID
Lauren Zalla, Projecto LINKAGES/USAID
Ana Diaz, Projecto LINKAGES/USAID
Giuseppe Cristino, Projecto LINKAGES/USAID
Joana Cardão, USAID
Armando Cotrina, USAID

**Funding Source:** UNC, FHI 360 through USAID LINKAGES Project

**Location:** Luanda, Benguela, Bié and Cunene provinces, Angola

**November 2015**

**v.1.5**

**Table of Contents**

I. ACRONYMS .......... 5
II. SUMMARY .......... 7
III. COLLABORATORS (IN ALPHABETICAL ORDER) .......... 8
IV. BACKGROUND .......... 11
A. ESTIMATING THE SIZE OF THE KEY POPULATIONS .......... 12
B. SAMPLING STRATEGIES FOR KEY POPULATIONS .......... 12
V. AIM AND OBJECTIVES .......... 14
VI. METHODS .......... 16
A. STUDY AREA .......... 16
B. STUDY POPULATIONS .......... 16
C. MAPPING READINESS ASSESSMENT PROCEDURES .......... ERROR! BOOKMARK NOT DEFINED.
POPULATIONS .......... ERROR! BOOKMARK NOT DEFINED.
OBJECTIVES .......... ERROR! BOOKMARK NOT DEFINED.
DATA ELEMENTS .......... ERROR! BOOKMARK NOT DEFINED.
METHODOLOGY .......... ERROR! BOOKMARK NOT DEFINED.
D. PLACE-PROGRAMMATIC MAPPING SURVEY PROCEDURES .......... 17
STEP 1: MAPPING READINESS ASSESSMENT, PREPARATION AND PROTOCOL ADAPTATION .......... 17
STEP 2: COMMUNITY INFORMANT INTERVIEWS .......... 18
STEP 3: VENUE VISITS AND MAPPING .......... 19
STEP 4: KEY POPULATION INTERVIEWS AMONG SITE WORKERS AND PATRONS; HIV, CD4 AND STI TESTING SELECTION OF SITES AND EVENTS FOR WORKER AND PATRON SURVEYS AND TESTING .......... 21
STEP 5: FEEDBACK AND DATA USE .......... 26
RISK ASSESSMENT AND COLLECTION OF BIOLOGIC SPECIMENS .......... 26
TEST RESULTS .......... 30
INDICATIONS FOR ANTIRETROVIRAL THERAPY (ART) .......... 31
STORAGE OF SAMPLES .......... 31
E. RDS SURVEY PROCEDURES .......... 32
STUDY SITE .......... 32
INCLUSION CRITERIA .......... 32
PEER RECRUITMENT AND SURVEY PARTICIPATION .......... 32
COUPON .......... 33

*Incentives* ... 34
*Collection of biologic specimens and testing* ... 35
F. Sample Size ... 35
G. Population Size Estimation Procedures ... 36
*PLACE-Programmatic Mapping Population Size Procedures* ... 36
*RDS Population Size Procedures* ... 36
H. Selection and Training of the Field Team ... 37

VII. DATA MANAGEMENT ... 39

A. Data Management ... 39
B. Coupon Data Management ... 39
C. Population Size Estimation Data Management in Cabinda ... 40
D. Study Identification Numbers ... 40
E. Data Quality Control ... 40

VIII. DATA ANALYSIS ... 40

A. RDS Analysis ... 40
B. PLACE Analysis ... 41
C. Population Size Estimate Analysis ... 46
*Population Size Estimation Analysis using PLACE* ... 46
*Population Size Estimation Analysis using Unique Object Multiplier and RDS* ... 48

IX. ETHICAL CONSIDERATIONS ... 49

A. Protocol Approval and Confidentiality Protection ... 49
B. Confidentiality and Data Protection ... 50
C. Consent Process ... 50
D. Potential Risks to Participants ... 50
E. Potential Benefits to Participants ... 51
F. Reimbursement for Participation ... 51
G. Possible Adverse Events from Survey ... 51

X. DISSEMINATION OF FINDINGS ... 51

XI. CHRONOGRAM ... 53

XII. APPENDICES ... 55

*APPENDIX 1. Mapping readiness assessment interview guide.* ... 55
*APPENDIX 2. Form A: Community Informant Interview Questionnaire* ... 84
*APPENDIX 3. Form B Venue Informant Interview Questionnaire* ... 89
*APPENDIX 4. Form C Patron and Worker Interview Questionnaire* ... 100

Appendix 5. Cabinda Study Questionnaire ..... 108
Appendix 6. RDS Non-response Assessment Form ..... 109
Appendix 7. Mapping Readiness Assessment Consent Form ..... 112
APPENDIX 8. Fact sheet for Community and Venue Informant Interviews (in lieu of consent form) ..... 114
Appendix 9. Programmatic Mapping and RDS Consent Form ..... 115
APPENDIX 10. Consent form for storage of specimens ..... 118
APPENDIX 11. RDS Coupon Example ..... 120
APPENDIX 12. Data Corrections Log ..... 122
APPENDIX 13. Population Size Estimation Contact Log ..... 123
Appendix 14. References ..... 125

# I. ACRONYMS

| | |
|---|---|
| AIDS | Acquired Immunodeficiency Syndrome |
| CDC | Centers for Disease Control and Prevention |
| CT | *Chlamydia trachomatis* |
| FHI | Family Health International |
| FSW | Female Sex Worker |
| GDP | Gross Domestic Product |
| HIV | Human Immunodeficiency Virus |
| INLS | Instituto Nacional de Luta contra a SIDA (National Institute Against AIDS) |
| INSP | Instituto Nacional de Saúde Pública (Natinoal Institute for Public Health) |
| IRB | Institutional Review Board |
| MSM | Men who have Sex with Men |
| MSW | Male Sex Worker |
| N | Number |
| NG | *Neisseria gonorrhoeae* |
| NGI | Next Generation Indicators |
| NGO | Non-Governmental Organization |
| NSP | National Strategic Plan |
| PEPFAR | U.S. President's Emergency Plan for AIDS Relief |
| PLACE | Priorities for Local AIDS Control Efforts |
| RDS | Respondent Driven Sampling |
| RDSAT | Respondent Driven Sampling Analysis Tool |
| STI | Sexually Transmitted Infection |

| | |
|---|---|
| SUAE | Serious Unanticipated Adverse Events |
| PLACE | Time Location Sampling |
| UAE | Unanticipated Adverse Events |
| UNAIDS | The Joint United Nations Programme on HIV/AIDS |
| USD | U.S. Dollars |
| USG | U.S. Government |

## II. SUMMARY

**Purpose:** Sex workers, men who have sex with men (MSM) and transgender women bear a disproportionate burden of HIV in Angola. This programmatic mapping study in Angola aims to identify and map priority prevention areas (PPAs) —also known as high transmission areas or “hot spots” in the district where HIV prevention activities are needed; to characterize and map high risk venues and events in these PPAs; and to describe characteristics of sex workers, MSM and transgender women socializing or working at these venues. We will also estimate the prevalence of HIV, syphilis, gonorrhea, chlamydia and trichomoniasis for these groups in the PPAs. The study will use dried blood spot or whole blood collection to assess viral load suppression in HIV-infected patients who are receiving antiretroviral therapy (ART).

**Participants:** In each geographic area of data collection, participants will include community informants, site representatives who are knowledgeable about the sites, and venue patrons and workers which will include sex workers, MSM and transgender women. Respondent driven sampling (RDS) may be used to recruit MSM, transgender women or sex workers.

**Procedures:** Interviewers ask community informants for names and locations of venues where people meet new sexual partners. Next, a representative at each venue is interviewed to learn about activities at the venue. Finally, interviews, HIV, STI and CD4 testing are conducted with workers and persons socializing at venues, as well as a separate sample of MSM and transgender women, to learn about their sexual behavior, HIV risk, and exposure to prevention programs, as well as their HIV status. Finger prick blood samples will be collected for rapid HIV, syphilis and Hepatitis B diagnosis. Additional finger prick blood and venous blood samples will be collected among HIV positive participants to obtain CD4 T-cell count and plasma HIV-1 RNA level. Vaginal and anal swab specimens for women and urine and anal swab specimens from men will be collected for subsequent gonorrhea, chlamydia and trichomoniasis testing.

# III. COLLABORATORS

## Principal Investigator

**Dulcelina Serrano**
Directora
Instituto Nacional de Luta Contra a Sida
Advises protocol development, data analysis and participates in dissemination of findings.

## Co-Investigators

**Marquez Gomez**
Vigilancia e Epidemiologia
Instituto Nacaional da Luta contra a Sida
Luanda, Angola
marquesgomes463@yahoo.com.br
Oversees preparations and conduct of survey, monitors implementation, advises data collection procedures and data analysis assists with interpretation of results, and dissemination of findings.

**Pedro Sapalalo**
Coordenador Geral
Tchikos Consultoria Empresarial
Via Expressa, Bairro Nova Urbanização II
Cacuaco, Luanda Angola
sapalalo@gmail.com
Training of interviewers and field staff, supervises data collection, data cleaning and analysis. Responsible for writing final reports and participation in dissemination of results.

**Michael Herce**
Research Assistant Professor
Division of Infectious Diseases
Department of Medicine
University of North Carolina at Chapel Hill
130 Mason Farm Rd., CB# 7030
Chapel Hill, NC 27599-7030 USA
michael_herce@med.unc.edu
Assists in protocol development, data analysis and dissemination of results.

**William Miller**
Strategic Information Advisor
Department of Epidemiology
UNC Gillings School of Global Public Health
University of North Carolina at Chapel Hill
135 Dauer Drive
2101 McGavran-Greenberg Hall, CB #7435
Chapel Hill, NC 27599-7435 USA
william_miller@unc.edu
Develops protocol, assists with survey preparation and procedures, monitors implementation, advises data collection procedures and data analysis, assists with interpretation of results, and participates in dissemination of findings.

**Kate Muessig**
Assistant Professor
Department of Health Behavior
Gillings School of Global Public Health
University of North Carolina at Chapel Hill
306 Rosenau, CB # 7440
Chapel Hill, NC 27599-7440 USA
kate_muessig@med.unc.edu
Assists in protocol development, data analysis and dissemination of results.

**Sharon Weir**
Research Assistant Professor
UNC Gillings School of Global Public Health
University of North Carolina at Chapel Hill
400 Meadowmont Village Circle, 3rd floor
Chapel Hill, NC, 27517 USA
Sharon_weir@unc.edu
Advises protocol development and data collection procedures, assists with interpretation of results, and participates in dissemination of findings.

**Lauren Zalla**
Program Associate
University of North Carolina at Chapel Hill
Gillings School of Global Public Health
135 Dauer Drive
2101 McGavran-Greenberg Hall, CB #7435
Chapel Hill, NC 27599-7435 USA
zalla@unc.edu

Assists in adaptation of questionnaires, protocol, data collection procedures, interpretation of results and dissemination of findings.

**Armando Cotrina**
HIV/AIDS Senior Technical Advisor
USAID Angola
Rua Huari Boumedienne, #32 Miramar
Luanda, Angola
acotrina@usaid.gov

Participates in supervision of overall study procedures and dissemination of results.

**Joana Cardão**
Project Management Specialist HIV/AIDS
Rua Huari Boumedienne, #32 Miramar
Luanda, Angola
jcardao@usaid.gov

Participates in supervision of overall study procedures and dissemination of results.

**Ana Diaz**
Program Manager
FHI 360
adiaz@fhi360.org

Participates in supervision of overall study procedures and dissemination of results.

**Giuseppe Cristino**
Technical Director
FHI 360
gcristino@fhi360.org

Participates in supervision of overall study procedures and dissemination of results.

# IV. BACKGROUND

The National Institute against HIV (INLS) in Angola along with technical partners including the USAID Mission in Angola, has requested that UNC-CH (under USAID PEPFAR LINKAGES project) conduct a mapping readiness assessment, programmatic mapping and bio-behavioral surveillance among key populations in five provinces of Angola in order to help address gaps in knowledge of the HIV epidemic, as well as increase capacity of national organizations to address HIV locally. Key populations in Angola are defined as sex workers (male and female), men who have sex with men and transgender women. UNC-CH will be working with an implementing partner in Angola to implement the study and to lead capacity building efforts and national coordination.

Currently, national level HIV-related data in Angola is limited to surveillance in pregnant women and to modeled Spectrum data. HIV prevalence for adults (15-49 years old) is estimated at 2.4% in Angola.[1] Approximately 223,350 adults and 29,103 children are living with HIV/AIDS. Estimates indicate that 53 percent (118,476) of people living with HIV/AIDS are in need of antiretroviral therapy (ART).[1]

In 2011, the prevalence of HIV among men who have sex with men in Luanda, Angola was found to be between 3.8 and 10.5%. 6,236 MSM were estimated to live in the capital.[2] The prevalence of HIV among female sex workers has only been studied in Cunene, an Angolan province bordering Namibia, where 8.5% of women engaging in transactional sex were estimated to be HIV positive.[3] No prevalence or size estimate studies have been conducted among key populations in Benguela, the second largest city, Bié province, where 5.8% of pregnant women were found to be HIV positive or Cabinda.[4]

In order to monitor and evaluate the prevention response for key populations outlined in the new HIV/AIDS Strategic Plan for 2015-2018, more information is required. Current gaps in information include the specific venues where key populations (sex workers, their clients, and MSM) can be reached by programs. It is in this context that National Institute against HIV (INLS) and its technical and financial partners (including USAID) plan to carry out a programmatic mapping, size estimation and sero-prevalence study among key populations. These activities will follow Respondent-driven Sampling (RDS) and the PLACE method, adapted specifically for key populations.

The PLACE method has been implemented in over 60 settings across 16 countries including Haiti, Jamaica, the United States, Russia, Tanzania and Zambia. Findings have confirmed that the method can be used to identify places where people meet new sexual partners. Findings have also confirmed that individuals socializing at such sites have higher rates of new sexual partner acquisition than reported by the general population (Weir et al. 2003, Weir et al. 2004, Tate et al. 2008).[5,6] A manual has also been written which describes the method in detail.[7]

RDS has been implemented in 69 countries for surveys among hard-to-reach or hidden populations. Universities, non-governmental and governmental organizations including the U.S. Centers for Disease Control and Prevention (CDC) have conducted RDS studies.[8]

**Rationale:**
The complexities of engagement in HIV care and treatment are well-identified in sub-Saharan Africa. Female sex workers, men who have sex with men and transgender women are susceptible to these complexities, while facing additional individual level barriers. LINKAGES will use the HIV Services Cascade as the overall strategic

framework for the project. The HIV cascade illustrates how individuals should ideally move through the continuum of HIV prevention, care, and treatment services to reduce HIV transmission as well as to ensure a high quality of life for key populations living with HIV. Use of the HIV cascade and the enhanced use of programmatic data associated with the cascade will allow the Ministry of Health and civil society organizations to identify “leaks” in the system where key populations are lost to follow-up or unable to access critical services in the comprehensive package such as HIV testing and counseling, sexually transmitted infection screening and treatment and other HIV services including enrollment on antiretroviral therapy. The WHO has recently released recommended global guidance on HIV prevention and treatment which includes an emphasis on key populations. The proposed work will align with the WHO guidance.

## A. Estimating the Size of the Key Populations

Estimating the number of sex workers, men who have sex with men and transgender women is important for both programmatic and research activities. There have been few studies estimating the prevalence of same key populations in Angola.

In 2011 the INLS, CDC and Tulane University estimated the size of the MSM population in Luanda. The estimated number was 6,236 MSM using unique object multiplier method.[2] Additional population size estimates are needed for prevention programming for sex workers, men who have sex with men and transgender women in priority areas.

In 2014 Population Services international estimated the size of the female sex worker population in Luanda to be 2200 using unique object multiplier method.

## B. Sampling Strategies for Key Populations

Sex workers, MSM and transgender women and have been disproportionately affected by the HIV/AIDS epidemics in many parts of the world including Africa. Accurate and precise data on the behaviors in each of these populations at risk is critical for tracking the direction of the epidemic, planning effective responses, and monitoring and evaluating those responses. The gold standard for acquiring HIV data is probability-based population surveys. Obtaining representative data on sex workers, people who inject drugs, MSM, transgender women and other populations at high risk therefore presents an enormous challenge. Because such groups are often a relatively small proportion of the total population, true population-based surveys need to be very large to include enough subjects for precise estimates. Moreover, due to the illegal and stigmatized nature of their behaviors, such populations are often under-recognized and under-reported in population-based surveys based on household interviews. On the other hand, recruiting subjects at locations where they are highly visible in large numbers, such as at service facilities or community venues, can be efficient but lacks validity in representation. Several approaches have been proposed to balance the need for recruitment efficiency and inclusiveness in representation. Snowball sampling increases efficiency, identification, and inclusion of hidden populations by having members of the target population recruit other members.[9] However, snowball sampling lacks validity in representation because the composition of the sample is dependent upon the choice of seeds (initial recruits) and short recruitment chains (the recruits of seeds). Venue-based sampling such as that employed by Priorities for Local AIDS Control Efforts (PLACE) seeks to approximate probability sampling by mapping the universe of venues where the target population can be found in large numbers, randomly selecting the locations for recruitment and systematically selecting participants from the venue. However, PLACE only includes the population that frequents selected venues. A method called respondent-driven

sampling (RDS) lends statistical rigor to conventional snowball sampling through longer recruitment chains, recruitment limits, and the collection of data used to statistically adjust for the biases inherent in how persons of similar characteristics are networked and likely to recruit each other.[10] RDS is being used for surveillance of populations at increased risk of HIV/AIDS in the United States and in more than 83 countries worldwide.[11] Although statistical questions about RDS remain, there is a growing body of experience using the method.

The RDS methodology is based on long-chain recruitment whereby members of the target population participating in the study refer other members of the target population to the study. In contrast to conventional snowball sampling, recruitment is limited to a certain number of people (two to three) in order to limit any individual's influence on sample accrual. Additionally, information on linkages between recruiters and recruits and the size of participants' social MSM networks are collected to statistically adjust for recruitment biases. An underlying assumption of RDS is that long-chain recruitment represents a first-order Markov process that reaches a dynamic equilibrium between the tendencies of persons with similar characteristics to associate with each other (homophily), the composition of the sample, relative social network sizes, and the underlying make-up of the target population.[12] Relative social network sizes affect a person's probability of being recruited into the study and their ability to recruit others. Data for the adjusted analyses are sufficient when the sample reaches ''equilibrium,'' that is, when additional waves of recruitment do not substantially change the composition of the sample with respect to key variables (e.g. socio-economic status). In practice, equilibrium is usually achieved in four to five waves for most variables. Recruitment chains begin with ''seeds'' or persons purposefully selected as members of the target population. Each seed receives uniquely coded coupons to be used to recruit other participants. Eligible people who presented with a coupon are consented, enrolled, and in turn given recruitment coupons until the sample size is reached.

A second sampling strategy is PLACE, a probability based method for enrolling members of a target population at times and places where they congregate. It is a useful strategy for sampling key population, because it concentrates resources where key populations can be reached. However, venue-based sampling for MSM has been used primarily in gay-identified areas of urban centers, where relatively hospitable field conditions prevail. Important questions remain about whether venue-based sampling is feasible in less gay identified areas and whether respondents interviewed in public places give honest and accurate sexual reports. PLACE is essentially a 5-step method in which identify the public places (such as hotels, bars, and events) where key populations socialize or meet new sexual partners. These places are potential intervention sites where the individuals most likely to transmit HIV can be accessed. Sites and events are identified by informants in the community. Sites are then mapped and a knowledgeable person at each site is interviewed. Next, key populations working or socializing at sites are interviewed about their sexual behaviors and exposure to HIV prevention at a sample of sites/events. Workers and patrons are also tested for HIV/STI at this time. Ideally, every member of the target population has a known, nonzero probability of being selected; every person selected agrees to participate; and everyone tells the truth. Such field conditions pose many challenges. Potential respondents typically are engaged in other activities, venues may offer little privacy, and individuals who decline participation cannot be systematically re-contacted as in telephone or household surveys.

Another issue concerns choice of venues. Achieving a representative sample requires not only high participation rates but also venues that are frequented by most of the target population.

Surveying in "low yield" or difficult (e.g., unsafe) venues is costly, however, in terms of money, time, and staff morale. For these reasons, some studies like the Young Men's Survey in the US excluded settings expected to yield fewer than 2 eligible men per. Such exclusion criteria tend to rule out venues where most attendees report exclusively heterosexual behavior and gay-identified venues where attendance is sporadic or low. This raises 2 questions: (1) Are there sufficient venues to implement PLACE in areas with small and dispersed MSM populations? And (2) Does excluding small venues substantially affect study results? The accuracy of self-reports obtained in public settings is questionable, especially when questions pertain to private and often stigmatized behaviors. The problem of response bias may be magnified in neighborhoods where concerns about homophobia and being overheard may be more pronounced. Thus, it is important to know whether reports of risky behaviors are substantially depressed when data are collected in public settings and when interviewers have relatively little time to establish rapport with participants.

To provide valuable information for HIV prevention programs in Angola the following public health surveillance activities will be carried out: a mapping readiness assessment in five provinces; a bio-behavioral survey among female sex workers in Cabinda province; and programmatic mapping for key populations with HIV/STI biomarkers in Luanda. The objectives of each activity are outlined in the next section.

## V. AIM AND OBJECTIVES

**Aim**

The mapping readiness assessment, programmatic mapping and biobehavioral surveillance in several priority provinces of Angola will assess communities' readiness for mapping sites where key populations can be reached, identify key people and places within the community, strengthen partnerships, provide information on venues where key populations can be reached by prevention programs, the severity of the HIV and STI epidemics among several key populations, the behaviors that increase people's risk of HIV infection, the coverage of HIV prevention services and the HIV testing and treatment cascade.

**Objectives**

**The programmatic mapping study will be carried out in 4 provinces:**

1) Luanda – defined as the municipalities of Luanda, Cazenga, Viana and Cacuaco.
2) Benguela – defined as the municipalities of Benguela, Lobito and Baia Farta
3) Bié – specific geographic areas will be defined as part of the mapping readiness assessment
4) Cunene – specific geographic areas will be defined as part of the mapping readiness assessment

Defined areas of provinces may change during the process of study protocol development.

For each province, the specific objectives are:

- Identify key community partners for programmatic mapping
- Define and describe key populations and types of sites to be mapped
- Assess the legal environment for key populations and mapping
- Assess data safety and security considerations and capabilities
- Gather perspectives from relevant stakeholders

- Gather perspectives from key populations about mapping
- Gather perspectives on social media and web-based platforms for meeting partners
- Gather perspectives from service and healthcare providers about mapping
- Gather perspectives from programmatic mapping team about preparedness

- Make a summary decision about the risks of programmatic mapping
  - Use the information collected in the activities above to create a comprehensive list of the risks of programmatic mapping in your setting.
  - Together with key stakeholders and representatives of the community, consider the information gathered in these activities to make a decision about whether to recommend moving forward with programmatic mapping.
  - Create a step-by-step plan to address each risk identified.
- Assess the appropriateness of respondent-driven sampling vs. venue-based sampling for sex workers (FSW), men who have sex with men (MSM) and transgender women
- Assess the need for incentives for participation in bio-behavioral survey
- Assess the logistics of key populations participating in a bio-behavioral survey
- Assess interest, location and services desired at drop-in centers
- Evaluate times and locations for mobile HIV testing and counselling or other services
- Assess receptiveness and logistics of using a mobile phone data collection, unique identifier code and follow-up of key populations over time
- Increase the capacity of HIV prevention response
- To identify and map priority prevention areas (PPAs) - also known as high transmission areas or "hot spots" for key populations.
- To characterize and map high risk venues and events in each PPA where key populations meet new sexual partners.
- To characterize the men and women who visit high risk venues and events and women who work at these venues and identify gaps in HIV prevention services (HIV Testing and Counseling (HTC), condoms, and syndromic/etiologic STI management).
- To characterize key populations: sex workers, men who have sex with men and transgender women that visit venues in Luanda.
  - To estimate the prevalence of HIV, the percent of key populations with early HIV infection, among people with HIV the percent who is eligible for ART, and the percent virally suppressed.
  - To estimate the percent of people at each stage of the HIV continuum of care between HIV diagnosis and viral suppression.
  - To estimate the prevalence of syphilis, Hepatitis B, gonorrhoea, chlamydia and trichomoniasis
- To inform stakeholders about high-risk areas for HIV transmission
- To facilitate provincial action plans for HIV prevention among key populations

**A bio-behavioral survey among female sex workers will be carried out in Cabinda province.**

The specific objectives are:

- Assess the sexual behavior and other risk behaviors associated with HIV transmission among female sex workers

- Evaluate the health-seeking behavior including harm reduction and HIV testing among female sex workers
- Describe the demographic characteristics of female sex workers
- Estimate the size of the female sex worker population through various multiplier methods
- Develop the capacity and strengthen the national HIV surveillance systems for key populations
- Provide information on female sex workers that will inform public policies and services and assist the government of Angola, international partners and other local organizations in strategic planning
- Estimate the prevalence of HIV, syphilis, Hepatitis B and C and co-infections among female sex workers.

## VI. METHODS

### *A. Study Area*

The programmatic mapping will be conducted in four (4) provinces: Luanda, Benguela, Bié, and Cunene. These provinces are labeled on Figure 1. These four provinces were selected as priority areas by USAID Angola and the INLS based on HIV prevalence data, concentration of the general population and knowledge of key populations from previous studies. Luanda is the capital and the largest city of the Republic of Angola. The current population size of the city's metro area is estimated at 6.5 million, approximately 25% of the entire population of Angola (2014 estimate). The city is subdivided into seven (7) municipalities, with Luanda being the busiest and most popular municipality for entertainment venues.

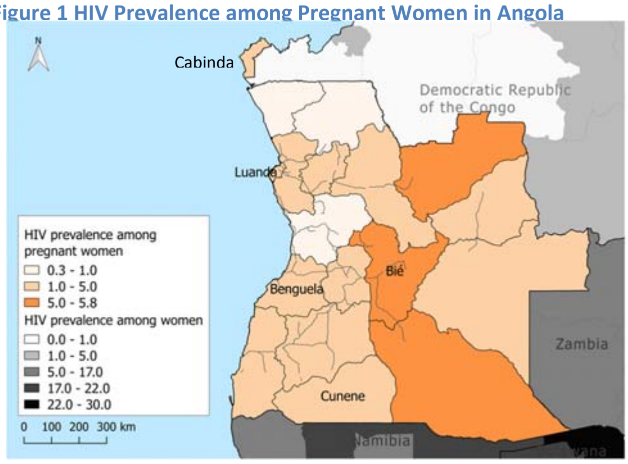

Figure 1 HIV Prevalence among Pregnant Women in Angola

Benguela is divided into 10 municipalities, the majority of the population residing in Benguela, Lobito and Baia Farta. Bié is divided into nine (9) municipalities and Cunene into six (6) municipalities. The mapping readiness assessment will permit definition of study areas based on the concentration of key populations.

### *B. Study Populations*

The study populations are sex workers, MSM and transgender women, at least 15 years of age who are residents of a to-be-defined geographic area at time of interview. International definitions of key populations follow:

- Sex worker (from the UNAIDS Guidance Note on HIV and Sex Work): "female, male and transgender adults and young people who receive money or goods in exchange for sexual services, either regularly or occasionally". This definition includes those who occasionally exchange sex for gifts. It does not include people younger than 18.
- Men who have sex with men: MSM is an abbreviation used for 'men who have sex with men' or 'males who have sex with males'. The term 'men who have sex with men' describes males who have sex with

males, regardless of whether or not they have sex with women or have a personal or social gay or bisexual identity. This concept is useful because it also includes men who self-identify as heterosexual but have sex with other men. However, abbreviations should be avoided whenever possible. Writing out the term is preferred.

- Transgender persons: ‘individuals whose gender identity and/or expression of their gender differs from social norms related to their gender of birth. The term transgender persons describes a wide range of identities, roles and experiences which can vary considerably from one culture to another.’

Operational population definitions and inclusion criteria will be established either through the mapping readiness assessment or during data analysis based on participants’ responses to the behavioral questionnaire.

### C. PLACE-Programmatic Mapping Survey Procedures

PLACE is a cross-sectional study. The general PLACE protocol can be found in English at:

http://www.cpc.unc.edu/measure/tools/hiv-aids/place

The programmatic mapping is based on the PLACE approach: identify the public places (such as hotels, bars, and events) where key populations socialize or meet new sexual partners. These places are potential intervention sites where the individuals most likely to transmit HIV can be accessed. Sites and events are identified by informants in the community. Sites are then mapped and a knowledgeable person at each site is interviewed. Next, key populations working or socializing at sites are interviewed about their sexual behaviors and exposure to HIV prevention at a sample of sites/events. Workers and patrons are also tested for HIV at this time. The full protocol will be carried out in 4 provinces of Angola.

There are five steps in PLACE:

1. Mapping Readiness Assessment, preparation, protocol adaptation in consultation with PLACE technical advisory committee, and district selection
2. Community informant surveys
3. Site visits and mapping
4. Patron, worker and key population interviews and HIV, STI and CD4 testing
5. Feedback and data use

#### *Step 1: Mapping Readiness Assessment, preparation and protocol adaptation*

As described above, Individual and/or focus group interviews will be conducted with members of key populations and service delivery organizations to develop a typology of sites where key populations can be reached as well as a typology of the major sub-groups within each key population. The guiding principle will be to identify site and group typology that can be used to inform the provision of services.

In addition, formative interviews will be used to adapt the protocol to the Angolan context, including obtaining appropriate terminology, formats for data collection, training materials, and protocol reviews. A Pilot will be implemented first and the protocol revised as needed.

Initial meetings with stakeholders will be used to identify the ideal categories of knowledgeable informants for the Community Informants survey (Step 2). Given these objectives, the inception meeting will be vital for

planning the survey as well as engaging the stakeholders about the role of programmatic mapping in HIV prevention at the district level. Participation at this meeting will be invited from technical, political and civil society groups that have potential roles in HIV prevention programs.

By the end of each meeting, provincial stakeholders will have a shared awareness of the role of programmatic mapping in the district HIV prevention, as well as guiding survey activities within the urban and rural areas and ideal categories of informants.

***Step 2: Community Informant Interviews***

Approximately 500 brief 10-20 minute anonymous Community Informant interviews will be conducted with persons knowledgeable about the community to identify the names of venues and events where people go to meet new sexual partners and where key populations can be reached.

The brief interviews will be conducted to identify venues and events where people meet new sexual partners. Respondents will be asked specifically about places where sex workers and men who have sex with men and transgender women go to meet partners or socialize. The respondents will be selected by interviewers based on their knowledge of the community. Trained interviewers will ask these community informants to identify venues and events where people meet new sexual partners, including sex workers, MSM and transgender women using Form A (see Appendix 2).

A list of venues and events named by community informants will be compiled for use in the second phase of data collection.

The informants will be sought from the categories of informants in the PLACE protocol and unique additional groups identified from the district inception workshops. Efforts will be made within each sampled area to ensure broad representation of the different categories of knowledgeable community informants. Bar managers, taxi drivers, police, security guards, cleaning women, street cleaners, market sellers, sex workers, STI clinic patients, health workers, truckers, college students, and street sellers have proven to be knowledgeable informants in other PLACE studies. Networks of MSM will also be contacted as informants. Recruitment will occur wherever the target types of informants can be found.

Prospective community informants will be approached by interviewers to voluntarily participate in the survey. They will be asked a few questions about their own characteristics and asked to identify venues or events where people go to meet new sexual partners. Specific questions will be asked about MSM venues and sex worker venues. Venues may include places such as bars and restaurants, streets, parks and public events.

Data collection form "Form A" (Appendix 2) is used in this step, and is used to:

- Collect a minimal amount of characteristics about the informant
- Lists the sites/venues identified by the informant and obtains additional information about each site.

Interviewers will be trained in administering community informant interviews during a two-day training. Also during this first training, interviewers will be trained in the PLACE method in general, ethical considerations of research, the importance of confidentiality during the study, and general interviewing techniques. The survey team will be trained to seek out the different informants and interview them in the local language. Translation

of the PLACE tools to the local language will be done at the training workshop to ensure that the appropriate language is used for interview. Due to the multi-lingual nature of most provinces, efforts will be made to have the tool translated into 2-3 languages appropriate for each province. Province-based interviewers will help in anticipating and interpreting the names and location of sites and venues provided by the informants.

*Recruitment of Community Informants*

The implementation team will brainstorm a list of the types of people likely to be knowledgeable about venues and set a target number for each type of informant to be named. Setting a target number for each type of informant helps to ensure all types of venues will be named. Bar managers, taxi drivers, police, security guards, cleaning women, street cleaners, market sellers, sex workers, STI clinic patients, health workers, truckers, college students, and street sellers have proven to be knowledgeable informants. Networks of sex workers, MSM and transgender women will also be contacted as informants. Recruitment will occur wherever the target types of informants can be found.

*Ethical Considerations for Community Informant Interviews*

Because community informants are only asked to name venues and events in the community and not to report information about their personal behavior, there is no risk involved in this phase of data collection. No personal identifying information is collected.

*Consent Process for Community Informant Interviews*

Interviewers will explain to **community informants** the purpose of the study, type of questions asked, and that no identifying information will be collected from them. Community informants will not require formal consent as the data collected from these informants is unrelated to their personal characteristics and is centered on the PPA and/or venue of interest. Community informants will receive a fact sheet about the study with contact number for the local and UNC researchers. They are asked their age in order to confirm eligibility (age 18 or older are eligible). Once eligibility is determined, the interviewer will read a short description of the study and the type of questions that will be asked.

*"Hello. My name is _______ and I am working with Tchikos/the National Institute against HIV/AIDS on a study that will improve HIV prevention programs here. I would like to ask you some questions about where people go to meet new sexual partners around here. I can offer you this Fact Sheet that has more information about the study."*

The interviewer will offer the respondent a Fact Sheet (see appendix) that contains information about the study, including contact information within Angola in case questions arrive after the interview. Next, the interviewer asks if they are willing to participate. If they are willing, the interview begins.

### *Step 3: Venue Visits and Mapping*

In this step, trained interviewers visit venues reported by community informants to verify the existence of the sites. Interviewers visit their assigned venues, identify a person knowledgeable about each venue (ideally someone from a key population), and then request an interview.

Sex workers, MSM and transgender women community informants will be asked to name the places where key populations receive services including NGOs. Venue visits will include visits to all organizations providing services to sex workers, MSM and transgender women.

Interviewers will be trained in the questionnaire to be used for interviewing a venue representative, as well as in using a GPS device to measure the latitude and longitude of each venue (see Appendix 3).

Internet sites will be visited as well. Efforts will be made to assess the number of people registered at the website and whether or not people registered at the website visit the public sites identified by the community informants. Additional venues and events will be obtained from information obtained on the internet sites. Site visits will be conducted at these new sites as well.

Information collected from venue visit interviews will be used to describe areas where there is risky behavior and to inform prevention programs. The list of venues in operation will be used as a sampling frame for the next phase of data collection, interviews with patrons and workers of venues. Maps will be created using the data to show the geographic distribution of venues and the coverage of prevention programs. Maps can also indicate the location of priority venues for interventions.

*Recruitment of Venue Informants*

Each pair of interviewers visits their assigned venues, identifies a person knowledgeable about each venue, and then requests an interview. The knowledgeable person at a venue may not be a manager or owner of that venue. At places like taxi stands, street corners, or parks, these interviews are not conducted with people in a position of leadership over the location. In such situations, the interviewer pair chooses a person who is likely to be familiar with the venue, but is not a manager, owner, or in a position of leadership. For example, a food vendor who regularly sets up near a city park could be approached for an interview about the park. While not in a position of responsibility for the park, this person is likely to know about the types of people that visit there. The field coordinator can be consulted by the interviewers for advice before going to the venue, if necessary.

Site representatives will be asked to participate in an interview about the site that will last 20 to 30 minutes. The questions asked include questions about the type of people who come to the venue, activities at the venue, whether people meet new sexual partners at the venue, and whether the respondent believes it would be feasible to have an HIV prevention activity at the venue sometime in the future. The interviewer is not asking for commitment to an onsite program, but an indication of feasibility. Commitment to an onsite program cannot be provided in any case by many of the respondents, as they will be people without any management responsibility for a venue.

There is one questionnaire used in this step (Appendix 3):

- Form B: Site/Event Verification Questionnaire – obtains characteristics of the site or event, including number and type of patrons, activities onsite, and HIV prevention coverage onsite; one form is completed for each site/event.

Responses to the questionnaire will be recorded on a smartphone. Each site and event visited will also be geo-located using the smartphone.

GPS coordinates of each venue are also obtained so that the geographic distribution of venues can be displayed on a map.

*Ethical Considerations for Venue Visits and Mapping*

Because knowledgeable people interviewed at venues are only asked questions about the venue and not about their personal behavior, there is no personal risk involved in this phase of data collection. No personal identifying information is collected from the respondent.

Maps made using the data from this phase of data collection will not indicate the location of vulnerable populations such as sex workers, MSM and transgender women. Venue maps will only indicate the distribution of venues and the coverage of prevention programs. Maps of priority venues for interventions may include venues where sex workers, MSM and transgender women visit, however the presence of specific populations will not be indicted.

*Consent Process for Venue Visits and Mapping*

Interviewers will explain to **site informants** the purpose of the study, type of questions asked, and that no identifying information will be collected from them. Site informants will not require formal consent as the data collected from these informants is unrelated to their personal characteristics and is centered on the PPA and/or venue of interest. Site informants will receive a fact sheet about the study will contact number for the local and UNC researchers.

Knowledgeable people at venues are not asked any personal information. They are asked their age in order to confirm eligibility (age 18 or older are eligible). Once eligibility is determined, the interviewer will read a short description of the study and the type of questions that will be asked.

*"Hello. My name is _______ and I am working with Tchikos/the National Institute against HIV/AIDS the national on a study that will improve HIV prevention programs here. I would like to ask you some questions about this place to find out about people who come here to socialize or meet sex partners and about HIV prevention that has occurred here. I can offer you this Fact Sheet that has more information about the study."*

The interviewer will offer the respondent a Fact Sheet (see Appendix 8) that contains information about the study, including contact information within Angola in case questions arrive after the interview. Next, the interviewer asks if they are willing to participate. If they are willing, the interview begins.

### *Step 4: Key population interviews among site workers and patrons; HIV, CD4 and STI testing Selection of Sites and Events for Worker and Patron Surveys and Testing*

Individual interviews and HIV testing with male and sex workers, men who have sex with men and transgender women will be performed in a sample of sites and events visited in the previous step. Social mobilizers will assist interviewers in identifying sex workers, men who have sex with men and transgender women at venues. At venues where social mobilizers accompany interview teams, all people identified by social mobilizers as potentially people from key populations will be interviewed. Additionally, people at venues will be approached randomly by interviewers and will not be screened for eligibility based on history of sex worker or same-sex practices. Through random selection, some participants may not be sex workers, MSM or transgender people.

Sites will be selected using a systematic fixed-interval sampling strategy with the probability of selection proportional to the size of the site. The size of a site is defined by the number of people socializing at the venue during a busy time as reported by the site informant. A sampling frame of sites that were confirmed to exist and be in operation and those that were temporarily closed during site visits (Step 3) will be constructed, with sites and events sorted by province/geographic area, type and size. Prior to interval selection, venues eligible for individual interviews are sorted by geographic location, as indicated by geographic code, and size of venue.

*Inclusion Criteria for PLACE*

Male and female participants who meet the following eligibility criteria will be invited to participate in the project:

- At least 15 years of age at time of interview and not accompanying or running an errand for parents
- Recruited at selected venues
- Consent to participant in a face-to-face structured behavioral survey and provide written informed consent with initials or an X

Additionally participants that meet one of the following inclusion criteria for key populations will be offered additional STI testing:

- At least one sexual contact for cash in the last 12 months
- Man or transgender woman that has had oral or anal sex with another man in the past 12 months
- Identification as a male-to-female transgender woman

*Recruitment of sex workers, men who have sex with men and transgender women at venues*

In this step, trained interviewers visit the sampled sites and events to interview eligible and consenting key populations present at that time. The team of survey interviewers will be accompanied by a team of people from the district health office trained in HIV counseling and testing.

Upon arrival, a field supervisor will speak with the person in charge of the site and explain the study and HIV testing that will be offered free of charge to participants. They will ask permission to begin requesting the participation of workers and patrons. It may be necessary to visit the venue at a time that the workers are less busy, for example in late afternoon or early evening before patrons arrive for an evening of socializing.

After being informed about the survey and testing, patrons and workers will be asked to participate. Social mobilizers will assist interviews in recruitment of patrons and workers who are from key populations. As most venues are expected to be small, all patrons and workers will be approached to participate. If more patrons or workers are present at a given site than can be feasibly interviewed in the time allotted to field staff, a systematic sample of patrons and a systematic sample of workers will be approached and asked to participate. If a patron or worker does not agree to be tested for HIV, the interview will not be carried out. The patron or worked will be screened for age eligibility. Persons who do not meet the inclusion criteria will not be interviewed. The respondent may be asked to move to an area of the site or near the site in order to preserve

privacy of responses. Once a respondent finishes the interview, they will be brought to the HIV counselors for testing and counseling.

The interviewer will use a structured questionnaire on a smartphone to interview respondents (see Appendix 4). The interview will take 20 to 30 minutes and the testing and counseling will require extra time. The survey instrument includes questions about socio-demographic information, sexual behavior, and exposure to HIV prevention programs. With participants of sufficient literacy, a portion of the sensitive questions about sexual behavior will be administered with the interviewer reading the questions and the respondent marking answers on a separate pre-printed answer sheet that will then be placed in an envelope without the interviewer seeing the responses. Either electronic data collection tools, i.e. mobile phones/tablets, or paper forms will be used to interview site workers, patrons and key populations.

There is one questionnaire used in this step (Appendix 4):

- Form C: Patron and Worker Questionnaire – obtains sociodemographic characteristics of the individual being interviewed, their sexual history and behavior, and prior exposure to HIV testing/prevention activities.

HIV testing will be conducted independently in or near the venue by a trained counselor and tester from the District Health Team. The HIV testing and counseling provided will include a rapid HIV test (using a blood spot from a finger prick) and the standard national counseling protocol, including pre- and post-test counseling (Determine HIV 1/2, Alere/Abbott, Waltham, MA, USA and Unigold HIV, Trinity Biotech, Bray, Ireland). Any person testing positive will be asked to give another drop of blood for a confirmatory HIV test. In the case of conflicting results from the two tests, a third test will be done on site. HIV test results will be provided on site along with post-test counseling. Counselors will follow up with participants testing positive for HIV to link them to additional health services in accordance with national guidelines.

Finger-prick blood samples will be collected to measure the CD4 T-cell count of HIV-positive participants using a rapid CD4 T-cell count analyzer, such as the PIMA Analyzer (Alere, Waltham, MA, USA). Based on the results of the CD4 test, patients will be referred to appropriate ART clinics for medical care and treatment. Venous blood will be collected from HIV positive consenting participants for posterior viral load quantification using Xpert Viral Load or PCR. Dried blood spots (DBS) will be collected for all consented participants. Samples will be collected by trained project staff. Participants will receive a finger stick to obtain blood sample according to the methods described in the Abbott DBS Collection Reference Guide (Abbott Laboratories, Abbott Park, IL, USA). Samples will be transported to a central laboratory site for quantitative viral load assay. All specimens must be processed within seven days of receipt at laboratory. Remaining samples will be stored at the testing laboratories in gas-impermeable zip closure storage bags in an -80C freezer for additional testing, as funding becomes available. Participants will be informed of their viral load results within one month of testing.

Men and women identified as at increased risk based on the interview will be offered additional STI testing. People who have been paid for sex or men who have had sex with other men in the past 12 months will be offered testing for syphilis, hepatitis B, gonorrhea, chlamydia and trichomoniasis. Syphilis and Hepatitis B serologic testing will be conducted using finger stick blood and an onsite rapid test. In addition, women will be asked to provide a self-collected vaginal specimen and men will be asked to provide a urine sample for testing

to detect current gonorrhea and chlamydial and trichomonal infections. Men who have had sex with another man and transgender women will be asked to provide a self-collected anal swab for further testing for chlamydia and gonorrhea at the study laboratory. Patrons or workers will be asked to collect these specimens in a restroom onsite. Interviewers will wait outside the restroom to receive the specimens. Participants will be given a phone number and an ID number with which they can collect the results of viral load, gonorrhea, chlamydia and trichomoniasis testing two weeks after participating.

Interviews with sex workers, MSM and transgender women will be conducted at all venues named as places where these populations can be found until the target number of respondents is reached. Our target is to interview 600 sex workers, 600 MSM and 200 transgender women at sites. However, a specific target cannot be determined until the end of the site verification phase. Interviewers will ask sex workers, MSM and transgender women about other venues where key populations can be reached. The list of venues will be expanded until the target number of key population participants has been reached.

The field supervisor will notify the venue owner and manager that the team will conduct the survey 1 or 2 weeks prior to recruitment and as soon as the team reaches the venue on the specific day and time. The coordinator will also be responsible for completing the Data Corrections Log (Appendix 11) and ensuring the appropriate information is collected prior, during and after the recruitment event.

*Ethical Considerations for Individual Interviews with Venue Patrons and Workers*

The individual interview does ask questions about the participant's own sexual behavior. Because sites where patron, worker, MSM and transgender women participation is requested are public, the interviewer will ask that he or she move to a different location within or outside the site in order to preserve privacy for the interview. The interviewer will seek a location where no one can visually observe the responses as they are recorded or can overhear the questions and responses. No identifying information will be collected, and written informed consent using initials or an X will be requested in order to preserve privacy to the greatest extent. To protect confidentiality and reduce social desirability bias where the in participants responds to questions with an answer he or she thinks is the correct or safe behavior, a number of questions regarding sensitive information could be completed by the participant on their own. If the participant is comfortable responding on the smartphone, the interviewer will ask for the participant to record their responses on the smartphone rather than tell the answers to the interviewer. The pros and cons of self-administered questions vs. interview administered questions will be assessed in the mapping readiness assessment given that in some cultures people may trust an interviewer more than they would an electronic device. Safety and literacy can also influence the feasibility of asking participant to respond on the devices.

For the HIV testing and counseling portion of the study, participants will be asked to enter a private area where their identity cannot be observed and where they cannot be overheard. In some districts this may be a vehicle outfitted with testing kits and supplies, and in others it may be possible to occupy a room or office nearby a cluster of sites where the participant can go for private testing and counseling. After post-test counseling, the participant may be given paper materials about HIV, HIV prevention and contact information for HIV/AIDS treatment and support providers. All participants regardless of their test results will receive the same information.

Because the interviewers will be residents of the areas where the survey is conducted, it will be important that respondents be interviewed by someone unknown to them. Not only does this protect the privacy of the respondent, but it also can prevent coercion. Interviewers will be instructed to not collect survey responses from potential patrons or workers at venues, or participants in the RDS study, with whom they are already acquainted.

The manager or owner of a site will give permission for interviews and HIV testing of workers to occur at the site. This may exert undue influence over workers to agree to consent to the study. In order to minimize the risk of coercion, interviewers will offer to talk to workers who do not want to participate for a period of time equal to the duration of the interview and testing process, so that the manager or owner of the site does not know whether a worker participated or not.

*Consent Process for Individual Interviews with Venue Patrons and Workers*

Interviewers will explain to site patrons and workers the purpose of the study, type of questions asked, and that minimal information will be collected from them. The interviewer will review an informed consent statement that describes risks and benefits of participating in the interview, HIV and STI testing, along with the procedure. Subjects will initial or write an "X" on the consent form acknowledging their consent to participate. They will then be provided a copy of the consent form to keep (Appendix 9).

Site patrons and workers, MSM and transgender women will be required to provide written consent by initialing or marking an "X" on the consent document. This mode of consent avoids excluding the significant portion of the potential subject pool that is not be able to sign their name and diminishes the social, psychological, and economic risks to participants should a breach of confidentiality occur, as initials would be more difficult to definitively link to a given subject. We do not collect subject names on the survey questionnaire itself, in order to protect participant confidentiality/privacy given the sensitive nature of some of the questions regarding individual sexual and drug use behavior.

Subjects aged 15-17 who wish to participate in the study will be allowed them to provide independent consent which would be documented with their initialing or marking an X on a written consent form. Subjects age 15-17 will be asked if at the time of the interview, they are accompanied by their parents or if they are running an errand for their parents. Only minors who are not accompanied or running an errand will be asked to participate. Participation in the study may facilitate access to the crucial service of HIV testing for adolescents who may face greater stigma or embarrassment requesting testing at other clinics. Adolescents aged 15-17 have been included in many previous PLACE studies, as they are often among those at highest risk for HIV and other sexually transmitted infections. Furthermore, most of the adolescents eligible for this study will have been identified by nature of visiting a venue where people go to meet sexual partners.

A separate consent will be requested for storage of dried blood spots and unused urine, vaginal swab specimen and/or plasma (Appendix 10). Participants may participate in the survey and not consent to storage of specimens. However, people approached that do not consent to HIV testing will not be eligible for participation.

*Incentives*

Participants recruited through the PLACE methodology will not be given an incentive. Participants will not have to spend pocket money on transportation and will benefit from onsite HIV and STI testing.

### *Step 5: Feedback and data use*

If electronic data collection tools are used, data will be entered and cleaned during field work. If paper data collection forms are used, following field work data entry will be completed in Angola. Data analysis will subsequently be conducted by both the implementing partner in Angola and UNC. Stakeholders in HIV/AIDS prevention and control activities in each province will be invited to participate in a workshop presentation of the results and to discuss the priority action plan based on the sites / people at risk identified. A data use workshop will be conducted to train Ministry of Health and other organization implementing HIV prevention programs for key populations in programmatic planning based on mapping and survey data.

### *Risk assessment and collection of biologic specimens*

All participants will tested for HIV using a rapid test and finger prick at or near the recruitment venue. Based on the behavioral questionnaire described above, participants that have sold sex, identify as transgender or are men who have had sex with another man in the past 12 months will be offered additional STI testing for syphilis, Hepatitis B, gonorrhea, chlamydia and trichomoniasis. Participants that meet the definition for key population will be tested for syphilis and Hepatitis B using a rapid test and finger prick at or near the recruitment venue. Participants that test positive for syphilis with a rapid treponemal test will be offered a confirmatory test, which will be conducted at the study laboratory. Participants will be given a phone number and instructions about where and when to collect their results.

The types of tests and specimens performed on participants from the different populations are outlined in Table 1. Briefly, after consent and completion of the behavioral interview, trained counselors will provide HIV and STI pre-test counseling in a private setting. The nurse or lab technician will instruct participants how to collect vaginal swabs, urine samples and anal swabs. Among female sex workers a vaginal swab, among male key populations first-catch urine and anal swabs will be collected to test for *Neisseria gonorrhoeae*, *Chlamydia trachomatis* testing and *Trichomonas vaginalis*. Samples will be immediately stored according to the laboratory procedures manual.

**Table 1 HIV and STI testing schematic by population approached at venues and defined in behavioral questionnaire**

| Population | HIV rapid test | Syphilis rapid test | Hepatitis B rapid test | Vaginal swab | Urine specimen | Anal swab | CD4 rapid test | Venous blood |
|---|---|---|---|---|---|---|---|---|
| Female sex workers | X | X | X | X | | | | |
| Male sex workers | X | X | X | | X | X | | |
| Men who have sex with men | X | X | X | | X | X | | |
| Transgender women | X | X | X | | X | X | | |
| General population | X | | | | | | | |
| HIV /syphilis positive | | | | | | | X | X |

The justification for the collection of anal swabs is that there have been increases in anal gonorrhea, chlamydia, including strains that cause Lymphogranuloma venereum (LGV) among men who have sex with men in various countries.

A nurse or laboratory personnel will conduct rapid HIV tests with the Determine HIV 1/2 test (Alere/Abbott Laboratories, Waltham, MA, USA) using whole blood obtained by finger prick. A negative test will be considered as a true negative, and the result will be reported to the patient after post-test counseling. A positive test will be confirmed by the Uni-gold HIV test (Trinity Biotech, Bray, Ireland). It is estimated that the HIV test results for the rapid tests will be available after 20 to 30 minutes. Results will be recorded on a HIV test results form.

If the Uni-gold test is positive, the result will be communicated to the participant as a positive result during post-test counseling. The participant will be advised that all positive results with rapid tests will be confirmed by viral load testing at the study laboratory. If results are indeterminate (initial test positive and confirming test negative) a third test will be done for confirmation from the blood already collected. The participant will be counseled about the result and the importance of obtaining follow up, and provided a reference card with his or her code number to obtain the results from the viral load test at the study laboratory in two weeks. The counselor will also advise participants on the recommended referral options for following up HIV and notification of sex partners.

Biologic samples will be stored  appropriately as specified in the laboratory standard operating procedures (SOPs).

Female participants that meet the definition for female sex worker will be given a flocked swab from the Xpert CT/NG Vaginal Specimen Collection Kit and asked to collect a vaginal swab specimen in the venue restroom. The participant will return the swab to study staff who will then insert the swab into the Xpert CT/NG collection tube, break off the swab stick at the top of the tube and screw on the top. Study staff will label the tube with the participants ID sticker and note the type of specimen and the date and time of specimen collection. The Xpert CT/NG Specimen Collection Kit is designed to collect, preserve and transport specimens from symptomatic and asymptomatic individuals prior to analysis with the Cepheid Xpert CT/NG Assay. When the study team completes data collection for that day, swab specimens will be transported to the laboratory for testing on the Gene Xpert Instrument Systems. Swab samples in Xpert CT/NG Swab Transport Reagent tubes are stable up to 60 days at 2 °C to 30 °C before testing with the Xpert CT/ NG Assay.

Male participants who meet the definition for MSM or transgender women will be asked to provide first-catch urine (approximately 50 to 60 mL of the initial urine stream) into a urine collection cup free of any preservatives. Collection cups will be marked at the level of 50 mL using a permanent maker (all the way around the cup) to guide participants, however, field staff will instruct participants not to discard urine if the volume exceeds the marked line. Urine collection cups will be labeled with the patient ID sticker and study staff will note the date and time of specimen collection on the cup. At the end of data collection for a given day, all urine specimens will be transported to the study laboratory.

Men who have sex with men and transgender women intercourse will be instructed on collection of an anal specimen using a Xpert CT/NG Specimen Collection Kit designed to collect, preserve and transport patient *Chlamydia trachomatis* and *Neisseria gonorrhoeae* DNA in specimens from symptomatic and asymptomatic

individuals prior to analysis with the Cepheid Xpert CT/NG Assay. Anal specimens are collected from patients using flocked swabs included in the kit. Swabs are broken off into the transport reagent tubes to elute organisms and stabilize DNA. Study staff will label the tube the participant ID sticker and note the date and time of specimen collection on the tube. When the study team finishes data collection for the day, swab specimens will be transported to the laboratory for testing on the Gene Xpert Instrument System. Anal swab specimens will be stored at 2-30 °C and processed within 15 days.

Among HIV positive participants, nurses or lab technicians will collect additional finger prick blood for the rapid CD4 analysis. CD4 testing will be conducted on site using a PIMA CD4 analyzer (Alere, Waltman, MA). The CD4 test takes approximately 20 minutes for processing. Participants will be given the result of their CD4 test by a trained counselor.

Additionally, the laboratory personnel will take a 10 ml sample of venous blood from participants who test positive for HIV and a set of dried blood spots for posterior viral load testing. Whole blood should be collected in EDTA, EDTA-PPT, or ACD collection tubes and will be centrifuged at the study laboratory to separate the plasma and red blood cells per the manufacturer's instructions. A minimum of 1 mL plasma is required for the Xpert HIV-1 Viral Load Assay. Whole blood will be centrifuged and plasma will be stored at 2-8 °C for up to 6 days, prior to testing. Alternatively plasma specimens are stable frozen at ≤ -18 °C.

Any pregnant women (regardless of HIV status) receive a rapid syphilis test in order to ensure quick identification and treatment of syphilis-infected pregnant women to prevent congenital syphilis. The nurse will ensure pregnant women with positive rapid syphilis tests receive immediate IM benzathine penicillin G, and treated pregnant women will referred to a health center once per week for two additional weeks for therapy. Other serum samples including VDRL or RPR will be done for pregnant women as for other participants.

The nurse will refer any person reporting genital ulcers, men reporting urethral or rectal discharge or women reporting vaginal discharge for STI treatment at local health center. The nurse will also provide vouchers for partners (as many as requested by participant) to access free care and treatment at the clinic, NGO or other nearby clinical facility.

Finally, the nurse or counselor will provide a “Results Card” (Appendix X) with a participant number (that will be linked to interview and lab results) and will explain to the participant how they can use this card and get final results of the STI tests taken that day, along with free treatment if any of the results are positive. Participants may choose to refuse collection of one or several of the specimens listed here. Table 2 presents the types of biological specimens and the location where processing will occur.

**Table 2 Specimens and laboratory tests**

| Specimen | Test | Laboratory | Volume |
|---|---|---|---|
| Whole Blood | Determine and Unigold HIV rapid tests | Study venue | Finger prick |
| Whole Blood | CD4 count, PIMA Analyzer | Study venue | Finger prick |
| Whole Blood | Dried Blood Spots for future analysis | Study venue | Finger prick |
| Whole Blood | Determine TP rapid syphilis test | Study venue | Finger prick |
| Whole Blood | Determine HBsAg rapid Hepatitis B test | Study venue | Finger prick |
| Serum | RPR or VDRL for syphilis | Study Laboratory, Luanda | 10 ml whole blood in EDTA, EDTA-PPT, or ACD collection tube |
| Serum | Xpert Viral load test | Study Laboratory, Luanda | |
| Vaginal swab from women | Xpert CT/NG *(C. trachomatis/N. gonorrhoeae)*<br>Xpert TV *(Trichomonas vaginalis)* | Study Laboratory, Luanda | Swab |
| Urine from men | Xpert CT/NG *(C. trachomatis/N. gonorrhoeae)*<br>Xpert TV *(Trichomonas vaginalis) n=100* | Study Laboratory, Luanda | 20 ml |
| Anal swab from men | Xpert CT/NG *(C. trachomatis/N. gonorrhoeae)* | Study Laboratory, Luanda | Swab |

*Quality Control Procedures*

All study personnel involved in specimen collection, handling, processing and testing will undergo training and regular supervision. The surveillance project includes detailed laboratory procedures manuals outlining standard procedures. Regardless of laboratory where testing was done, all positive and a representative sample of 10% of negative specimens will be retested to ensure validity of test procedures. Internal and external quality control.

*Clinical Specimen Collection Flow*

Samples tested on site with results provided same day:

- HIV Determine and Uni-gold with finger prick whole blood
- SD Bioline Syphilis 3.0 rapid syphilis test with finger prick whole blood
- Determine HBsAg Hepatitis B rapid testing with finger prick whole blood

Samples collected at venues for processing at study laboratory:

- Venous blood sample for syphilis confirmatory testing with RPR or VDRL and viral load testing using Xpert Viral Load Assay (patients with positive rapid test results for syphilis and HIV, respectively

  - Whole blood may be held at 2-8 °C for up to 72 hours, prior to preparing and testing the specimen.

- Dried blood spots for posterior HIV and STI testing through PCR will be stored at ambient temperature with desiccant bags or in low temperature freezer based on availability

- 20 ml urine for *Neisseria gonorrhoeae, Chlamydia trachomatis* and the first 100 samples for *Trichomonas vaginalis* (men meeting key population definitions) stored at ambient temperature for up to 24 hours.

- Rectal swabs for men and transgender women meeting key population definitions

- Vaginal swabs for *Neisseria gonorrhoeae, Chlamydia trachomatis* and *Trichomonas vaginalis* from women meeting key population definition

- Samples tested at study Laboratory will provide results to participants within 2 weeks of receipt using the participant ID number

  - RPR or VDRL for confirmation of syphilis
  - *Neisseria gonorrhoeae* (vaginal, urine or anal, as applicable)
  - *Chlamydia trachomatis* (vaginal, urine or anal, as applicable)
  - *Trichomonas vaginalis* (vaginal or urine, as applicable)
  - HIV viral load

Quality control for HIV testing will be conducted for all positives and 10% of negatives

### *Test results*

As noted earlier, rapid HIV test results will be returned to participants by a trained counselor after the necessary pre- and post-test counseling. Negative results will be provided 30 minutes after testing. Participants with positive results will receive a preliminary positive results 30 minutes after being tested and the confirmatory results in 2 weeks. HIV-positive participants will be referred to HIV treatment sites for evaluation and therapy if needed.

If found to be HIV-positive, participants will be encouraged to refer all partners for HIV testing at a predetermined local clinic or VCT site, and the HIV-positive participant will be referred to a site where they will be able to access free treatment. However, such partners and their test results will not be a part of this survey and will not be linked in any fashion with the HIV-infected subjects in this survey. There will not be a separate effort by study clinic staff participating in this survey to try to contact (telephonically or personally) any partners of any subjects in this survey (regardless of test results) since this would constitute a clear violation of the confidentiality agreement by the survey's PI with the subject. The subject will be given the option for him/her to contact his/her sexual partners for follow-up.

Participants with signs and symptoms of STI will referred for treatment at a local health center. Participants will be provided as many partner notification slips as requested and will be encouraged to refer their partners to a pre- determined local clinic for free treatment.

Participants will receive a test results card with their survey number and the place and date to collect other test results. This card should be presented at the study laboratory to collect STI and viral load results. Results for tests processed at the study laboratory, such as N. gonorrhoeae and C. trachomatis and viral load testing will be available in 2 weeks. Participants with positive test results will be referred for treatment.

If participants loses their results card and the only link to their laboratory tests a unique identifier code will be used to return STI and viral load test results. During specimen collection participants will be assigned a unique identifier code based on personal information such as birth year, first letters of their parents' names and gender. This unique identifier will not permit study staff to identify the participant. It will be recorded with the specimen roster in case the participant returns to collect results without their results card. If a participant comes to the laboratory without their results card, laboratory staff will ask the participant a series of questions to reconstruct the unique identifier code, look up the results and return the test results to the participant.

### *Indications for antiretroviral therapy (ART)*

HIV-positive patients, confirmed by two rapid tests and a CD4 test will be referred to ART programs in clinical sites managed by the Angolan government. A referral letter will be prepared with the participant's name, demographics and results of HIV, CD4 and viral load tests. This letter will be provided to the patient in a closed envelope and will not be linked to any survey materials. HIV- positive patients will be provided with information on the closest ART site and attendance times. At the ART site, patients will receive a clinical evaluation and ART will be initiated according to these findings.

The government of Angola has a treatment program for HIV-positive patients. There is at least one treatment site in each of the cities where the survey will take place. Although the point of entry for highly active antiretroviral therapy (HAART) in Angola is a CD4 count < 350 or a clinical diagnosis for AIDS, people are evaluated and also provided counseling on healthy living (e.g., safe water, food, information about hygiene and activities to avoid), given counseling to avoid transmission to sex partners or unborn children, evaluated for tuberculosis, and where indicated provided prophylactic antibiotics against opportunistic infections.

### *Storage of Samples*

Patients will be asked for consent to store dried blood spots and leftover urine and serum samples after testing is complete. Blood and urine samples will be stored with no demographic or clinical data for future use at a specimen bank at the INLS Laboratory in Luanda. The samples will be stored with a code and without any personal information that could link them to the participant. Samples may be used for testing for infectious diseases, immune markers of infection or HIV phylogenetic. If a researcher wishes to use these specimens in a future survey, they will need to submit a research proposal to the INSP Ethics Committee who will act on the participants' behalf to weigh the risks and benefits of future testing of specimens, and whether the results can be linked to the coded questionnaire data from this survey. Participants can choose not to have their specimens stored and still be part of this survey.

## D. RDS Survey Procedures

### Study Site

The behavioral survey employing RDS methodology will be conducted at a public health center in Cabinda, a site known to the local population. At this site, private rooms will be provided for interviews and local coordination of the survey, with access restricted to the research team. This site will be designed to provide an inviting and comfortable environment for participants. Days and times of operation will be established after the start of field work. If necessary, alternative RDS sites (i.e. mobile van, participant's house, etc.) will be identified for participants who request more privacy. A field study coordinator will be responsible for managing the team and for ensuring compliance with all procedures described in this protocol. At least three interviewers, one receptionist, and one field coordinator will be at the study site during hours of operation.

If MSM and transgender women in any province cannot be effectively recruited through public sites, we will seek the assistance of community leaders and members in recruiting five to eight persons from the MSM and transgender community to serve as "seeds" or as persons who will recruit peers from their own communities via a system of recruitment invitations. The number of seeds may be increased if half of the recruitment chains fail to produce new recruits a month or two before data collection ends. We will attempt to identify a diverse (i.e. different age groups, ethnicities, socio-demographics, and education level) and popular (i.e., those having many network connections) group of seeds to start data collection.

### Inclusion Criteria

For the Cabinda study, female participants who meet the following eligibility criteria will be invited to participate in the project:

- At least one sexual contact for cash in the last 12 months
- At least 15 years of age at time of interview
- Resident of Cabinda province at time of interview
- Possession of a valid RDS coupon
- Consent to participate in a face-to-face structured behavioral survey

The interviewer will explain the study to the participant and ask for written inform consent (Appendix 9).

### Peer Recruitment and Survey Participation

We will have two types of participant through RDS: 1) seeds, who are the initial study participants recruited by field staff and investigators; and 2) new study participants recruited by previous study participants.

Each seed will receive three unique invitation coupons to be distributed to their peers which will constitute the first "wave" of the study. The number of seeds may be increased if recruitment slows or halts in the first month of data collection.

Times and locations where the invitee can participate in the study will be listed on the coupon (see Appendix 10) along with a telephone number of a field supervisor so that participants can call and schedule an appointment to participate.

All people who arrive with a coupon at a predetermined study time and place will be checked for eligibility. Interviews and testing will be conducted at sites convenient to participants and investigators. If eligible, team members will explain study procedures and ask participants to sign the informed consent form before administering a 20-25 minute standardized questionnaire on paper form or smartphone. HIV and STI testing and counseling will also be administered per the SADC study protocol and laboratory manual. As with site patrons and workers, the counselor will follow up with RDS participants testing positive for HIV or other STI to link them to additional health services in accordance with national guidelines.

After participating in the study, each participant will receive a gift, and three new coupons to give to their peers inviting them to participate in the study. Before receiving the three invitations, each participant will be trained on the peer recruitment process and be told to give one coupon to each of three friends, relatives or people they are close to, who are over 15, who are residents of Luanda, to whom they have seen or spoken to in the past 30 days and who received money for sex in the past 12 months. This process will be repeated until the sample size is achieved. After distributing their invitations to their peers, participants will return to collect their secondary incentives and will be asked to complete a non-response assessment to determine the number of invitations distributed, number of people who refused to accept the invitations, reasons given for refusing to accept the invitations, and type of relationship with each person they attempted to recruit (see Appendix 6).

Once 75% of the sample size is reached and equilibrium of the sample is achieved, the study supervisor may inform study staff to reduce the number of recruitment coupons dispensed to participants from three coupons to two coupons to one coupon. Determining when to end RDS recruitment will take several factors into consideration including the number of recruits still needed to reach the sample size, number of valid (unexpired) coupons still in the community, and average number of participants enrolling at the study site every day.

Various sociodemographic variables and type of sex work will be monitored during data collection to identify and prevent recruitment bottlenecks. Recruitment bottlenecks occur in RDS studies when participants recruit many peers with similar characteristics, such as youth or street-based sex workers. If it seems that during data collection, the sample will be heavily influenced by a particular subpopulation, the study coordinator and investigator can reduce or eliminate the number of coupons given to a particular recruitment chain. Investigators can also start new seeds that have characteristics that are underrepresented in the sample (such as telephone based sex workers) in an attempt to obtain a more representative sample.

As recruitment nears sample size, equilibrium of the sample will be assessed using RDSAT. If both sample size and equilibrium criteria are met, no new coupons will be distributed and the duration of the expiration periods will be narrowed to reduce the chances of persons trying to enroll in the study once it has ended. During every interview, all participants will be informed that no more interviews will be conducted once the sample size is reached. Furthermore, interview sites will have a protocol in place for explaining the termination of the survey to those who show up once the sample size is reached.

### *Coupon*

Each coupon will be uniquely coded in order to link seeds with new individuals in their recruitment chain. Each coupon will have an identifying code of the recruiter and a unique code for new recruitments, allowing us to

track social networks. Names and other personal identifying information will not be collected on any of the study forms. The coupons will be produced in such a way as to make them difficult to reproduce.

The coupon will consist of two parts and each part will bear an identical recruiter code. When the recruiters invite a new person they must hand over one of the parts of the coupon to the new recruited person, with the other part being kept by the recruiter. When the recruiters come back for their incentives, they must hand over their half of the coupon, which must match the half handed out to the new participants. In this manner the participants will remain anonymous, no names or personal identifying information will be collected on any study form.

Coupons will have the following elements:

- ID number (coupon number)
- Survey name (the target population and exact purpose will not be mentioned)
- Interview site address
- Telephone number of interview site
- Days and hours of operation
- Activation date: Date before which the coupon may not be used for enrolment. This date may vary; initially, the activation date will be two days after the coupon's issuance date. At the discretion of the study coordinator or the PI, recruits presenting coupons before the activation date may be accepted.
- Expiration date: Date after which coupon should not be used. Initially, coupons will be valid for two weeks. This time period may be extended or shortened if coupon return rates are below or above expectation and as the sample size approaches the target. At the discretion of the study coordinator or the PI, recruits with coupons that are within a set period of days past the expiration date may still be accepted.
- Information about its use and validity

A coupon may be invalid if: expired, tampered with, unreadable, or already used. Invalid coupons will be retained and stamped "VOID." Valid coupons of recruits undergoing screening for eligibility will be retained and stamped "USED." Recruits who are re-scheduled for a future visit will have their coupons returned to them. Re-scheduled visit dates may be past the coupon expiration date without rendering the coupon invalid.

#### *Incentives*

MSM, transgender women and female sex workers that participate in the peer referral study (RDS), whether they are the initial "seeds" or a subsequent recruit, will receive mobile phone credit or other gift worth 900 Angolan Kwanzas (9 USD). The participant must consent to completing the survey and undergoing HIV and STI testing in order to be eligible for any amount of the incentive. If they subsequently refuse the HIV test and/or do not complete a majority of the questionnaire, they will not receive any incentive.

If an RDS participant successfully recruits one additional person to participate, they will receive an additional mobile phone credit or other gift worth 400 Angolan Kwanzas (4 USD) beyond the primary incentive, for a total incentive worth 1300 Kwanzas (~13 USD). If s/he recruits two additional persons to participate, s/he will receive an additional phone credit or gift worth 800 Kwanzas beyond the primary incentive, for a total incentive of 1700 Kwanzas (~17 USD). If s/he recruits three additional persons to participate, he will receive an

additional 1200 Angolan Kwanzas (~12 USD) beyond the primary incentive, for a total of 2100 Kwanzas (~21 USD).

### *Collection of biologic specimens and testing*

All participants will tested for HIV using a rapid test through finger prick blood sample at the health center study site. Participants will be offered syphilis, Hepatitis B, and Hepatitis C testing based on the SADC protocol. Nurses will collect venous blood for STI testing and provide results when participants return to the study site on a later date.

## *E. Sample Size*

The sample size will be calculated based on the budget and the estimated number of sex workers, men who have sex with men and transgender women that are thought to be reachable (Table 3). For PLACE/Programmatic mapping the exact number of each population interviewed will depend on the site identification, verification and patron and worker interview steps.

Approximately 2000 men and women will be interviewed and tested for HIV at venues in Luanda. We expect to recruit approximately 600 sex workers, 600 men who have sex with men and 200 transgender women. Around 600 participants could be from the general population. Approximately 400 female sex workers will be recruited in Cabinda using RDS.

**Table 3 Summary of interviews to be completed by province**

| Population | Luanda | Benguela | Bié | Cunene | Cabinda | Total |
|---|---|---|---|---|---|---|
| Community informant interviews | 500 | 500 | 500 | 500 | 0 | 2000 |
| Venue informant interviews | 700† | 500 | 500 | 500 | 0 | 1500 |
| **Behavioral interview and HIV/STI testing** | | | | | | |
| Female sex workers | 600 | 400 | 400 | 400 | 400 | 2200 |
| Men who have sex with men | 600 | 400 | 400 | 400 | 0 | 1800 |
| Transgender women | 200* | ǂ | ǂ | ǂ | 0 | 200* |
| Other men and women at venues | ǂ | ǂ | ǂ | ǂ | 0 | ǂ |

* Could be less based on the number and percent of people at venues who identify as transgender, has received money for sex and who are men who have had sex with a man in past 12 months. † If there are more than 700 venues are identified in Luanda, a sample of venues will be selected. **The sample must be confirmed by UNC staff prior to field work.** ǂ No defined sample size as sample will depend on distribution of key populations in venues sampled.

## F. Population Size Estimation Procedures

### PLACE-Programmatic Mapping Population Size Procedures

The process is to estimate the number of each type of key population at each site, sum up the estimates across all of the sites, adjust the estimate to take into account visits to multiple sites and mobility, and finally extrapolate the number to areas not covered by the mapping.

### RDS Population Size Procedures

We will use the unique object multiplier method to obtain a population size estimate of female sex workers in the province of Cabinda. The multiplier method is the most commonly used population size estimation method, in part because it is relatively straightforward and utilizes simple calculations. The unique object multiplier method involves the distribution of a set number of unique objects, such as key chains, to members of the target population. Unique objects will be distributed widely at specific locations where female sex workers congregate (i.e. bars, brothels, hotels, etc.). The goal is to distribute as many unique objects as possible, but no less than the agreed upon study sample size.

*Data Sources*

This method uses data from two overlapping sources to estimate population size. The first set of data comes from the number of unique objects distributed in a two week period by field staff two weeks preceding the implementation of the RDS survey. The second set of data comes from the RDS survey data, where study participants are asked whether they had received a unique object. The number of participants receiving a unique object is then multiplied by the inverse proportion of the percentage of RDS participants who received the unique object.

Source 1: Field staff distributing unique objects will report data regarding location and time of unique object distribution, and the exact number of unique objects distributed using the Population Size Estimation Form (Appendix 12).

Source 2: Relevant questions related to receipt of unique objects will be included in the RDS survey. These include:

1. Did you receive a [object] when contacted by a field staff in the past few months?
2. Can you show it to me?
3. Can you describe it to me? (if the [object] is described properly, show it to them)
4. Is this the [object] you received?
5. How many [objects] did you receive?
6. When did you receive this [object]?
7. Where did you receive this [object] (at what site)?

*Data Collection*

A standardized form (Appendix 12) will be used to collect data from staff distributing unique objects two weeks prior to the implementation of the RDS survey. Data will include a) Number of unique objects distributed to eligible female sex workers, b) Location of unique object distribution; and c) Time of unique object distribution.

*Sample Size*

The number of unique objects will be agreed upon by the project coordinator and organizations assisting in distribution. While increasing the number of unique objects may improve precision of the final estimate, there is no set formula to determine a specific number for distribution. Factors related to distribution of unique objects, such as logistics and human resources, will be considered. In general, the number of unique objects will be determined by the number of contacts the distributing organizations anticipate reaching within one to two weeks. The process for determining the sample size will be documented and included in study reports. The distribution of unique objects should saturate the target population so that by the end of the distribution period all people meeting the study definition in the study are have received the unique object.

*Implementation*

NGOs and other organizations will be asked to distribute a specified number of unique objects to female sex workers for a period of two weeks. Each person will receive only one object and will be asked to keep the object because they may be asked about it by another field staff in the near future. Field staff will determine whether the participant has previously received a unique object to avoid double counting. No personal identifying information will be collected.

To estimate population proportions, persons enrolled in survey will be asked if they had received the unique object and be asked to show or describe the object to the interviewer. Those who are in possession of the object or can recall it in sufficient detail will be counted as the proportion who received the unique object. Calculations are then made to estimate the population size.

### G. Selection and Training of the Field Team

All members of the field team will be contracted locally. The Project Coordinator overseeing both RDS and PLACE studies will be the first field staff member to be selected and will participate in the selection process of other field staff members. The study field coordinator for each province will be interviewed by the project coordinator and study investigators. Potential interviewers and receptionists will be interviewed by the project and field coordinators.

All members of the field team will be trained in the following:

- Introduction to RDS methodology (RDS team only)
- Introduction to PLACE methodology (PLACE team only)
- Study procedures and flow

- Interview techniques on how to obtain answers to sensitive and non-sensitive questions
- Data collection instruments
- Management of electronic instruments for data collection
- Ethical aspects involving human research
- Stigma and discrimination issues affecting MSM, female sex workers and transgender women
- Communication skills
- Confidentiality
- Data security

Theoretical and hands-on training will be conducted by study investigators and national and international consultants. During data collection, each field team will receive direct supervision by one of the study investigators to troubleshoot specific recruitment and data collection procedures and to anticipate and solve potential problems that might arise in the field.

Interviewers and supervisors will be provided specialized training on the questionnaire, the use of the smartphones and survey software, and interviewing procedures.

Specialized HIV counselors trained in and responsible for providing information about HIV and other STI, the HIV test, and where people can go for additional HIV services.

Nurses will conduct HIV, syphilis and Hepatitis B rapid testing at or near the study site.

Laboratory personnel: will process the VDRL or RPR, N. gonorrhoeae, C. trachomatis and viral load tests the study laboratory, deliver the results of the tests to the participants as they return for their results, and will also store, coordinate adequate transport, and prepare the samples.

Study staff, including supervisors, interviewers, nurses, volunteers and laboratory personnel will be provided a special training by MOH and UNC staff in order to carry out the study protocol and comply with study procedures. In addition, counselors, who will be selected from a pool of experienced, trained personnel from the MOH, will be provided a refresher training course on counseling skills and provided detailed information on how to refer patients found to be HIV- infected and on partner notification practices. All personnel working on the study will be provided special training on keeping information confidential. Experienced research, program and laboratory staff from the MOH and UNC will conduct the training.

Recruiters (interviewers and volunteers) and supervisors will be provided training on RDS recruitment methodology. RDS uses the practice of participant-driven recruitment and offers remuneration to participants who refer their peers to study sites. Interviewers and volunteers will be in charge of instructing the participants on the eligibility criteria, how to recruit other participants using coupons and the reimbursement for participation.

Laboratory staff and nurses will be given specialized training on the rapid HIV, syphilis, Hepatitis B and CD4 test, venipuncture and collection of blood, handling of urine specimens, vaginal and anal swabs. Laboratory technicians will be given specialized training on processing, storage, and transport of urine specimens, vaginal and anal swabs and venous blood for viral load testing. They will also receive refresher training on biosafety

and protocols they should use in the event of unexpected occupational exposure to blood or blood products. Training will include provision of a laboratory handling procedures manual.

## VII. DATA MANAGEMENT

### A. Data Management

Data will be collected using a smartphones to minimize data entry error. Electronic versions of the survey will be created by Tchikos and pre-tested for accuracy and relevance. Upon completion of the interview, the interviewer will press a "submit" button, which encrypts the data before sending it to a secure server in Angola, specified for data storage. This server is only accessible by the server manager and key study staff, and is password protected. When internet access is unavailable for submitting completed surveys directly to the server, interviewers will save the data on the smartphone until the end of the work day, at which time the tablet will be returned to the field supervisor or study coordinator. The field supervisor or coordinator will then download the data from all smartphones onto a password protected laptop or desktop. When internet access is restored, he or she will send the encrypted data to the server.

Data will be stored in a database generated by the software and transferred weekly via a secure FTP server managed by UNC or data download to UNC. Separate databases will be created for each survey and will contain data from the questionnaire for all participants. Data will be backed up daily and the data manager will run weekly cleaning and validation programs and provide feedback to the field to carry out appropriate corrective actions. These will include checking for screening criteria, incomplete interviews, and proportion of missing key variables. All data collected on smartphones will be stored on a secure server. Once the data collection phase is completed, data will be transferred to SPSS, SAS and RDSAT for analysis. The clean data sets will be moved by authorized personnel for long-term storage on UNC's secure server. Only members of the UNC research team for this study that are using the data will have access to the related data files on that secure server.

Only authorized, survey-related personnel will have access to any data, whether computerized or in hard copy. Electronic data will be stored in a password-protected computer in the study's coordinator's locked office. Hard copy and back-up disks will be stored in locked file cabinets. Databases containing study information, hard copy files, and consent forms will be destroyed five years after the completion of the survey. The data will be joint property of UNC and the INLS. Records and data will be maintained based on their recommendations and regulations. As per the separate data use agreement cleaned data sets will be transferred to the Dataverse at UNC's Odum Institute. Appropriate precautions will be taken to protect against indirect identification of individuals. People who which to obtain the data will have to complete a request explained their intended purpose for the data.

### B. Coupon Data Management

RDS coupon data will be managed using a coupon management software and stored into a database. This software will allow field staff to monitor participants, assign coupon ID numbers, verify study eligibility, track study participation, track number of incentives given out, monitor recruitment chain growth in real-time, and back up data. This database will contain the coupon number of each participant recruited, and coupon numbers provided to participants for peer recruitment.

### C. Population Size Estimation Data Management in Cabinda

Field staff will complete a form recording the site (by site code), time, and number of unique objects distributed. After field correction, forms will be faxed, e-mailed, or hand-delivered to the data manager, who will double-enter the data into a password-protected Microsoft Excel spreadsheet stored centrally at a site designated Tchikos. All data will be encrypted before being transferred via secure FTP server and will be backed up daily.

### D. Study Identification Numbers

For RDS, a unique RDS code will be assigned to all participants. The RDS code will be noted in the survey coupons and entered twice in control coded fields in smartphone. Each coupon will have a unique coupon number for new recruitments and will be numbered in such a way as to allow the coupon management system to track social networks.

For PLACE, a unique PLACE code will be assigned to all participants. This code will come from the specimen identification stickers. We will use the same PLACE code on all study forms, including test results form, questionnaire and specimens collected. The unique code will guarantee that all records for a participant can be reliably linked to one another. In the smartphone questionnaire, the PLACE code will be entered twice in control coded fields to detect any errors.

All participants will also be assigned a unique identifier code using a combination of gender, year of birth, day of birth, and the first two letters of their parents' given name. The unique identifier code will be piloted for acceptability as part of the mapping readiness assessment. The unique identifier code will be constructed by the nurse or counselor conducting HIV testing and entered on the test results record. This unique identifier code will allow participant to retrieve their STI or viral load test results if they have lost their unique RDS or PLACE code card. It will also allow for piloting a unique identifier code that could be used by outreach workers to avoid double-counting people reached during prevention activities.

### E. Data Quality Control

The field coordinator will be responsible for the flow of information and the quality control of the information flow between the venues, study site in Cabinda and the Tchikos.

Data quality control will begin in the pre-test phase, with the identification and replacement of unclear survey terms and questions. The second step in data quality control will occur during training of field staff, especially of interviewers who will be trained to ask personal and sensitive questions in a non-judgmental manner. The third step in data quality control will entail local and continuous monitoring of recruitment activities, protection of participant's rights, data collection, coupon management, and overall compliance with the protocol.

## VIII. Data Analysis

### A. RDS Analysis

RDS-based surveys will be analyzed with RDSAT or RDS-A, free software packages that adjust RDS-collected data for network sizes and recruitment patterns. Using RDSAT and NetDraw, a software for drawing social

networks, we will examine the equilibrium of key variables and recruitment patterns by key demographic characteristics to identify potential biases associated with recruitment (i.e. homophily, number of recruitment chains). We will exclude all data from seeds in the analysis and export RDSAT weights directly into SAS to conduct analyses not supported by RDSAT. We will also use RDSAT to generate population estimates for specific key indicators from UNAIDS[1] and PEPFAR[2] for behavioral surveillance purposes (Table 4).

### *B. PLACE Analysis*

PLACE-based surveys will be analyzed using survey specific commands in SAS to account for the cluster design and weights. Weights will be constructed to produce unbiased population-based estimates and will be based on the following information: attendance at venues, attendance at specific venues or cluster size. Non-response bias will also be assessed by comparing proportion of missing data by key characteristics. We will generate population estimates for specific key indicators (Table 4).

One study objective is to estimate the proportion of key populations at each step of the HIV testing and treatment cascade, the basis for the LINKAGES Project. Table 4 includes indicators for the percent of HIV positive people who are aware of their status, the percent of those positive that are enrolled in HIV care, the percent who have initiated ART, the percent sustained on treatment for 12 months and the percent who are virally suppressed. Other indicators focus on coverage of HIV prevention activities such as HIV testing, STI screening, condom and lubricant distribution, information on HIV, AIDS, STI and safe sex and the percent of the population that has received a package of interventions.

**Table 4 Key indicators targeting MSM used by FHI, UNAIDS, and PEPFAR for behavioral surveillance purposes.**

| Cascade Step | Indicator | Indicator # | Measure |
|---|---|---|---|
| Identify population size | Estimated total number of key populations in the program geographic area (denominator for KP_PREV) | PMP 1.1b (denom.) | |
| Reach people, Coverage | Percentage of people from key population who have received free condoms and lubricant in the past 12 months | WHO CCP-3, CCP-5 | Num: Number of respondents who received free condoms and lubricant in the past 12 months<br>Denom: Number of people interviewed |
| | Percentage of respondents who have been tested for HIV during the last 12 months and who know their results (prior to participation in the survey) | GARPR 1.9 & 1.13 | Numerator: Number of respondents who have been tested for HIV during the last 12 months and who know the results.<br>Denominator: Total number of respondents |

| Cascade Step | Indicator | Indicator # | Measure |
|---|---|---|---|
| | Percentage of people from key population who have been screened for STI in the past 12 months (prior to participation in the survey) | WHO SRH-4 | Num: Number of respondents who have been screened for STI in the past 12 months<br>Denom: Number of people interviewed |
| | Percentage of people from key population who have received information on HIV, AIDS, condoms or safe sex in the past 12 months (from an outreach or health worker)? | | Num: Number of respondents who have received information on HIV, AIDS, condoms or safe sex in the past 12 months?<br>Denom: Number of people interviewed |
| | Key population reporting they have received a combined set of health sector interventions | WHO PKG-2 | Condoms; lubricant; information on condom use and safe sex; HTC; STI screening; |
| | Proportion of key populations reached with individual and/or small group level HIV preventive interventions that are based on evidence and/or meet the minimum standard required (KP_PREV/CUSTOM INDICATOR) | PMP 1.1b | Num: Number of respondents who have received any of the above interventions in the past 12 months<br>Denom: Number of people interviewed |
| | Percentage of people from key population reached with HIV prevention programs | GARPR 1.7, 1.11, 2.1 | Participants who respond, 'Yes' to the following questions:<br>1. Do you know where you can go if you wish to receive an HIV test?<br>2. In the last 12 months, have you been given condoms?<br>Denom: Number of people interviewed |
| Test people | Proportion of key populations who received HIV testing and counseling services for HIV and received their test results (HTC-3, GARPR 1.9, 1.13 & 2.4) | 1.3b | Num: Number of people tested for HIV who receive their results<br>Denom: Estimated number of key population living with HIV in the program geographic area |

| Cascade Step | Indicator | Indicator # | Measure |
|---|---|---|---|
| Prevalence | Percentage of people from key population who are living with HIV | GARPR 1.10, 1.14 & 2.5 | Num: Number of people diagnosed with HIV<br>Denom: Number of people tested for HIV |
| | Percentage of people from a key population diagnosed syphilis | WHO O-3b | Num: Number of people diagnosed with active syphilis<br>Denom: Number of people tested for syphilis |
| | Percentage of people from a key population diagnosed gonorrhea | WHO O-3b | Num: Number of people diagnosed with gonorrhea<br>Denom: Number of people tested for gonorrhea |
| | Percentage of people from a key population diagnosed chlamydia | WHO O-3b | Num: Number of people diagnosed with chlamydia<br>Denom: Number of people tested for chlamydia |
| | Percentage of people from a key population diagnosed Hepatitis B | WHO O-3b | Num: Number of people diagnosed with HBV<br>Denom: Number of people tested for HBV |
| Awareness of status | Proportion of key populations who are HIV positive and knew their status | WHO 4 | Num: Number of people with HIV who have been diagnosed and received their results<br>Denom: Number of people living with HIV |
| Enroll PLHIV in care | Proportion of key populations with HIV enrolled in HIV care (WHO 1.3) | 1.6b | Num: Number of people with HIV who have been enrolled in HIV care<br>Denom: Number of people diagnosed with HIV |

| Cascade Step | Indicator | Indicator # | Measure |
|---|---|---|---|
| | Proportion of KP with HIV **currently** enrolled in care among those who tested positive (WHO 1.3 & HCT_TST) | PMP 1.10 | Num: Number of people with HIV who were currently enrolled in HIV care<br>Denom: Total number of key populations who tested positive for HIV and who know their results |
| Initiate on ART | Proportion of key populations **currently** receiving antiretroviral therapy (TX_CURR/CUSTOM INDICATOR) | PMP 1.12b | Num: Number of people with HIV who are taking ART<br>Denom: Number of people diagnosed with HIV |
| | Proportion of key populations currently receiving antiretroviral therapy among those who are currently enrolled in care<br>(TX_CURR/WHO 1.5 & WHO 1.3 & ART-2) | PMP 1.13 | Num: Number of people with HIV who are taking ART<br>Denom: Total number of key populations who tested positive for HIV, know their results and are currently enrolled in care |
| Sustain on ART | Proportion of key populations known to be alive and on treatment 12 months after initiation of antiretroviral therapy (TXT_RE/WHO 1.6) | PMP 1.14b | Num: Number of people with HIV who are taking ART for at least 12 months<br>Denom: Number of people diagnosed with HIV |
| | Percent of key populations with HIV known to be on treatment 12 months after initiation of antiretroviral therapy (TX_RET) | PMP 1.15 Core | Num: Number of people with HIV who are taking ART for at least 12 months<br>Denom: Number of key populations in the ART start-up group initiating ART at 12 months prior to the end of the reporting period |

| Cascade Step | Indicator | Indicator # | Measure |
|---|---|---|---|
| Suppress viral load | Proportion of key populations on antiretroviral therapy who are virologically suppressed (WHO 1.7, CUSTOM INDICATOR) | PMP 1.17b | Num: Number of people with HIV on ART whose viral load is less than 1000 copies/ml<br>Denom: Number of key populations in the ART start-up group initiating ART at 12 months prior to the end of the reporting period |
| | Proportion of key populations on antiretroviral therapy who are virologically suppressed among those tested for viral load (WHO 1.7) | PMP 1.18 | Num: Number of people with HIV on ART whose viral load is less than 1000 copies/ml<br>Denom: Number of key populations on antiretroviral therapy tested for viral load in the reporting period |
| | Proportion of viral load tests with undetectable viral load (<1000 copies/ml) (TX_UNDETECT) | PMP 1.19 Core | Number of viral load tests where the result is <1000 copies/ml<br>Denom: Number of viral load tests performed key populations on ART in the past 12 months prior to the beginning of the |
| Behavior | Percentage of sex workers reporting the use of a condom with their most recent client | 1.8; O-4a | Num: Number of sex workers who reported that a condom was used with their last client<br>Denom: Number of people who reported having received money for sex in the last 12 months |

| Cascade Step | Indicator | Indicator # | Measure |
|---|---|---|---|
| | Percentage of men reporting the use of a condom the last time they had anal sex with a male partner | 1.12; O-4e, O-4g | Num: Number of male or transgender respondents who report condom use at last anal sex with a male partner in the last 12 months.<br><br>Denom: Number of male respondents who have had sex with at least one male partner in the past 6 months. |
| Stigma | Percentage of key populations reporting stigma and discrimination | O-5a | Num: Number of people who report experiencing stigma or discrimination due to their sexual orientation, work or HIV status<br><br>Denom: Number of people interviewed |
| | Percentage of people from key populations who have experienced discrimination by health workers | NEEDS.7 | Num: Number of people who report experiencing discrimination by a health care worker<br><br>Denom: Number of people interviewed |

## C. Population Size Estimate Analysis

### Population Size Estimation Analysis using PLACE

1. For each area that was mapped, we will estimate the number of sites in each area by type of site and type of key population at site
2. We will estimate the number of each type of key population at each type of site
3. We will provide a map of sites indicating the number of key populations at each
4. We will estimate the total number of key population member in each zone and in the sub-national areas included in the estimate
5. We will extrapolate the estimate to include areas not covered
6. We will estimate the number of key populations missing from the estimate based on findings from formative research
7. We will refine the size estimates based on mobility of key populations, visits to multiple venues, and variety in frequency of attendance
8. We will use the size estimates to calculate coverage for components of the comprehensive program for key populations

The following steps for size estimation will be followed:

1. Review data to confirm completeness of site lists and adequacy of estimates of the number of key population members at each site. Check the quality of the data by comparing the number of sites named and the number of times each was named with the number of reports naming a site from community informants. Check that there are sites named in every area included in the mapping activity. Confirm that the interviewers included members of each key population and that targets for type and number of community informants were reached. Review the list of sites that were not located and re-visit a random sample to confirm that the sites do not exist.
2. Calculate a point estimate for the number of each key population at each site based on the reported range provided. At each site, informants will be asked to estimate the maximum and minimum number of key populations at the site at a busy time.
3. Separate the estimates from sites that were initially identified as sites with key populations from sites that were not reported to have key populations. The rationale for treating the two types of sites differently is that they are sampled with different sampling fractions and the expected number of key populations at the sites reported to have key populations will be higher than at the other sites.
4. Add up the median number of key populations at each site for an initial crude estimate of the size of each population.
5. Produce a map illustrating number of key populations at each site using the median for the size estimate.
6. Adjust estimates based on information about frequency of visiting sites, frequency of visiting more than one site in a day, and the percentage of sites that have key populations but were not initially identified as sites with key populations.
7. Use the formula below to estimate the size based on the key population sites and separately for the other sites. The formula is:

   $E_i = s_i(1\text{-}p_i) + (s_i * p_i / m_i)$

   $E_i$ = the estimated key population members in an area

   $s_i$ = estimate at the site level

   $p_i$ = Proportion of female sex workers solicit in two or more sites

   $m_i$ = Mean number of places a female sex worker solicit

   For example,
   If $s_i$ from all the key population sites is 10,000 and 10% solicit in other sites, and the mean number of places the 10% solicit is 2 then the estimate is
   10,000 (1 - 0.1) + (10,000 * 0.1 / 2)
   = 9000 + 10,000 * 0.05
   = 9000 + 500
   =9500

   The formula is reasonable because it counts all of who are only at one place and for the ones who go to more places, the percentage of that group is reduced because the people might have counted at more than one place. This could be repeated for key populations at Other Sites. If 500 were found at Other Sites, the estimate would be 10,000.

8. Use the area estimates to develop national estimates. National estimates are developed by extrapolation of data. One approach is to estimate the percentage of the male and female population who is a member of a key population in the areas where the mapping and size estimation activity was done. Apply the percentage estimated in one area to the areas that are similar.
9. Meet with stakeholders to review the estimates and ensure that the size estimates are reasonable and acceptable to the stakeholders.
10. The size estimates can be used as the denominator in coverage estimates. If the randomly selected members of the key population were asked questions about access to testing and other prevention initiatives, additional analysis of their responses would be useful.

Size estimates of populations at risk for HIV/AIDS infection help policy makers and program administrators understand the scope of the HIV problem, plan appropriate interventions, and allocate sufficient resources. Population size estimates will be used as denominators in calculating program coverage and producing estimates and projections of the HIV epidemic in areas where calculated. The data are intended to be used by the MoH, non-governmental organizations (NGOs), and other stakeholders for disease control purposes, specifically for program coverage planning and assessments.

### *Population Size Estimation Analysis using Unique Object Multiplier and RDS*

We will collect information on the proportion of sex workers, MSM or transgender women who received a unique object. Three sets of results will be produced with the multiplier by comparing number of individuals receiving unique objects with 1) RDS numbers, 2) numbers from the distribution log from Data Source 1, and 3) numbers collected from survey interviews from Data Source 2.

**The formula for estimating the population size using a multiplier is**

$$N = \frac{(n_1 \text{ x } n_2)}{m}$$

where N is the estimate of total population size; n1 is the number of individuals receiving unique objects; n2 is the number of individuals participating in the RDS; m is the number in both populations (i.e. the number of MSM who received unique objects and participated in the RDS)

**Calculation for the variance of the population size estimate and standard errors using the multiplier method**

Using the following formula derived from the Delta method we can calculate the variance of the population size estimate and 95% confidence bounds around our estimate:

V(S) ≈ (n12) * ( 1 - (n2/m) ) / ( m * ( (n2/m)3 ) ) + (r2) * n1

where V(S) is the variance of the population size estimate; n1 the number of MSM receiving a unique objects from data source 1; n2 is the total number of sex workers, MSM or transgender women interviewed in the RDS; m is the number of sex workers, MSM or transgender women interviewed in the RDS receiving a unique object; r is the ratio of m to n2.

**Assumptions**

Members of the sex worker, MSM or transgender population must have a chance of being included in both sources of data. Data from both sources will be evaluated for any potential barriers to inclusion of members of the target population. Where this assumption is violated, this will be clearly documented and reported, and other sources of data will be considered.

The multiplier method requires clear and consistent population definitions, time reference periods, and catchment areas between the different data sources. During preparation for implementation of multiplier activities and collection of unique object and survey data, these issues will be carefully considered, and investigators will ensure that definitions across data sources will be coordinated.

**Objective 8: To provide data to inform and guide future behavioral surveys and prevention strategies targeting** sex workers, MSM or transgender women in Angola**.**

Responses collected at a roundtable meeting with key stakeholders held at the end of data collection will be analyzed. We will invite all USG agencies, local NGOs, and key government officials to discuss how survey results can assist ongoing and future behavioral activities targeting sex workers, MSM and transgender women and how these results can advance priorities outlined in the Country Operating Plan (COP) and Angola's National Strategic Plan (NSP). Priorities for Local AIDS Control Efforts (PLACE) studies in identifying high transmission HIV/AIDS areas to improve HIV prevention program delivery. Combined data from objectives A, B and C will be used to guide the design of new prevention messages targeting sex workers, MSM and transgender women. All results will be shared with NGOs and other agencies working directly with sex workers, MSM and transgender women in Angola.

## IX. ETHICAL CONSIDERATIONS

### *A. Protocol Approval and Confidentiality Protection*

Any protocol involving human subjects must follow internationally accepted practices with assurance and confidentiality. The protocol will be submitted for local ethics approval to the INSP Ethics Committee and will be reviewed by the Institutional Review Board at the University of North Carolina. In addition to the survey protocol, the INSP ethics committee will review the content and language of the informed consent form to be sure that they can be easily understood by the survey participants and also to ensure that all aspects of this research survey are appropriately explained to the subjects prior to enrollment. In order to protect confidentiality, neither participants' names nor personal identifying information will be attached to any of the survey forms. All forms will be identified only by a unique personal numeric code. Survey records will be kept in a locked file cabinet in the main survey office at the Tchikos until completion of the project and then destroyed. All official protocol files (protocol and forms, IRB minutes and approvals) will be kept on a secure server at UNC. All consent forms will be kept at the Tchikos office and copies will be given to the participants. The database will be sent to collaborators at the UNC on a regular basis.

### B. Confidentiality and Data Protection

No personal identifying information will be collected as part of this study. All datasets will not have any personal information stored; each record will be identified by a unique study ID number and participants will be assigned a unique identifier code (UIC) based on personal information such as birth year and parents given names. The UIC components will investigated as part of the Mapping Readiness Assessment and finalized before the PLACE or RDS survey initiation.

Databases, computers and smartphones and forms will be protected. Databases will be password protected and only persons working in the study will have access. Laptop computers and study forms will be locked in filing cabinets or drawers when not being used by study staff.

All field team members will have to sign a data use and confidentiality agreement prior to the data collection phase.

### C. Consent Process

Trained interviewers will obtain written informed consent from all survey participants. Interviewers will initial or mark the consent form with an ‘X’ to verify that the content of the informed consent was explained and that the individual voluntarily agreed to participate. Participants will be informed about their rights to stop the interview at any time and to refuse any questions they do not feel comfortable answering.

### D. Potential Risks to Participants

Inconvenience to participants may occur due to the time required to complete the survey. A psychological risk due to the sensitive nature of the questions may exist. The survey will be administered through smartphone in a private setting and will not contain any personal identifiers. The risk of inadvertent disclosure of HIV or STI status to interviewers if positive also exists. Investigators are not required to report to the MOH HIV status or risk related behaviors. Participants will be told that they can refuse to answer any question they do not feel comfortable answering. Any interview in which physical injury, psychological, social or economic harm to participants is detected will be terminated immediately. Homosexuality and sex work are not effectually prosecuted in Angola/

There are minimal physical risks of bleeding, bruising, fainting or light- headedness and rare risk of infection related to needle sticks. However, the survey will hire only trained medical personnel and will ensure an adequate supply of new, sterile disposable needles. Survey participants may experience discomfort during genital exams and sample collection.

There is inconvenience to participants due to the time required to complete the survey and collect samples. There are also some psychological risks from learning that one has an STI or HIV, and the risk of inadvertent disclosure of HIV or STI status if positive. Every participant will receive pre-test and post- test counseling, preliminary HIV, syphilis and Hepatitis B and if applicable, CD4 results will be provided the same day of testing. Confirmatory results for syphilis and HIV as well as gonorrhea, chlamydia, trichomoniasis and viral load will be available in two weeks. Personnel at the clinics have been trained specifically to counsel clients regarding HIV test results. Participants found to be HIV or STI-positive will be referred to specialized care for further evaluation and treatment. Participants will be encouraged to notify their partners if they have been diagnosed with STI or HIV, this entails a risk of breakdown in relationships or other negative consequences. Strategies for

partner notification will be discussed during counseling and for HIV-positive participants, couples counseling will be offered.

### *E. Potential Benefits to Participants*

The benefits to the participant in the survey include education, testing and treatment for sexually transmitted infections, education on STI and HIV infection and counseling to prevent HIV transmission to partners. In addition, participants' sex partners will have the opportunity to be treated, minimizing the risk for re-infection with the STI and possible adverse sequelae. Participants with asymptomatic STI will have the opportunity for diagnosis and treatment using laboratory tests, which is above and beyond standard of care in Angola.

Other benefits include referral to an HIV specialist for complete examination and treatment. The country of Angola will benefit because information on HIV and STI prevalence and the population at different stages of the HIV treatment cascade will be available. The information collected in this survey will contribute directly to the design and evaluation of interventions among the surveyed groups.

### *F. Reimbursement for Participation*

RDS recruited participants will also receive a gift for participant and as motivation for bringing their invitees to the study site (maximum of three). These token gifts will include T-shirts, make-up, a small handbag or mobile phone credit valued at 900 Angolan Kwanas (~9USD). Remuneration for time and cost for participant-driven recruitment provided in this study is small enough to be considered non-coercive, does not cause undue inducement, is not excessive, and does not compromise the voluntariness necessary for informed consent. If the risk of harm from recruitment or participation is identified during the interview process, study staff can choose to withhold recruitment or remuneration.

### *G. Possible Adverse Events from Survey*

We do not anticipate any adverse events from these surveys since they are cross-sectional in nature.

Unanticipated Adverse Events (UAE) are defined as unforeseen problems involving risks to subjects or others encountered during the survey. This includes: 1) physical injury to a participant, 2) psychological, social, or economic harm to a participant, 3) breaches of protocol, such as breakdowns in the informed consent process, violations of confidentiality or data privacy of a participant, and complaints by participants or their representatives, and 4) any serious or continuing non-compliance with federal regulations by investigators or research staff. These events will be reported to the survey ethics committee and data monitoring group within 72 hours of the discovery of the adverse event. Any interview in which physical injury, psychological, social or economic harm to participants is detected will be terminated immediately.

## X. DISSEMINATION OF FINDINGS

The main actors in the HIV response in each province will be invited to participate in a workshop presentation of the results and hence the priority action plan based on the sites / people at risk identified. The workshops will be by region. The dissemination/data use workshops have the objective to translate evidence into service delivery planning and action.

UNC, FHI 360 and Tchikos will establish and strengthen community committees as well as strengthen the capacity of peer educators and case managers to collect, analyze and use routine M&E data with a view to quarterly stakeholder data review meetings in year two. Towards this end, the study team will develop tools for provide training/technical assistance on data interpretation and use for planning. Community committees will consist of key population community groups and networks that will serve as a mechanism for communities to provide feedback and to shape quality of program services.

Data collected from this study will be disseminated to different audiences at different times. A final report will be presented to the INLS and USAID. At the end of the study, a seminar presenting significant findings will be organized in Luanda and presented to all stakeholders, including civil society.

The results will also be disseminated to the scientific community through peer-reviewed publications and presentations at national and international scientific conferences. All data will need pre-approval from the UNC prior to dissemination.

[1]UNAIDS. Monitoring the Declaration of Commitment on HIV/AIDS: Guidelines on Construction of Core Indicators: 2008 reporting. Switzerland: WHO. 1-144.

[2]PEPFAR. Next Generational Indicators Reference Guide. Version 1.1. Washington DC, 2009. Accessed on December 8, 2009 at http://www.pepfar.gov/documents/organization/81097.pdf

## XI. CHRONOGRAM

Survey preparation activities began in May 2015 and field work and interviews are scheduled to begin in September 2015. It is anticipated that data collection, analysis, and dissemination will be finalized in June 2016.

| Activity | August | | | | September | | | | October | | | | November | | | | December | | | | January | | | | February | | | | March | | | | April | | | | May | | | |
|---|---|---|---|---|---|---|---|---|---|---|---|---|---|---|---|---|---|---|---|---|---|---|---|---|---|---|---|---|---|---|---|---|---|---|---|---|---|---|---|---|
| Signing contract | | | | | | | | | | | | | | | | | | | | | | | | | | | | | | | | | | | | | | | | |
| Development of protocols and interview guides | | | | | | | | | | | | | | | | | | | | | | | | | | | | | | | | | | | | | | | | |
| Request for ethics approval | | | | | | | | | | | | | | | | | | | | | | | | | | | | | | | | | | | | | | | | |
| Selection of participants for MRA | | | | | | | | | | | | | | | | | | | | | | | | | | | | | | | | | | | | | | | | |
| MRA data collection in Cabinda | | | | | | | | | | | | | | | | | | | | | | | | | | | | | | | | | | | | | | | | |
| Transcription of interviews in Cabinda | | | | | | | | | | | | | | | | | | | | | | | | | | | | | | | | | | | | | | | | |
| Coding and analysis of interview data | | | | | | | | | | | | | | | | | | | | | | | | | | | | | | | | | | | | | | | | |
| First Draft report for MRA completion | | | | | | | | | | | | | | | | | | | | | | | | | | | | | | | | | | | | | | | | |
| RDS recruitment of FSW in Cabinda (SADC study) | | | | | | | | | | | | | | | | | | | | | | | | | | | | | | | | | | | | | | | | |
| Data collection for MRA Luanda, Benguela, Bié and Cunene | | | | | | | | | | | | | | | | | | | | | | | | | | | | | | | | | | | | | | | | |
| Transcription of interviews | | | | | | | | | | | | | | | | | | | | | | | | | | | | | | | | | | | | | | | | |
| Coding and analysis of interviews | | | | | | | | | | | | | | | | | | | | | | | | | | | | | | | | | | | | | | | | |

| Activity | August | | | | September | | | | October | | | | November | | | | December | | | | January | | | | February | | | | March | | | | April | | | | May | | | |
|---|---|---|---|---|---|---|---|---|---|---|---|---|---|---|---|---|---|---|---|---|---|---|---|---|---|---|---|---|---|---|---|---|---|---|---|---|---|---|---|---|
| Final MRA report completion | | | | | | | | | | | | | | | | | | | ■ | ■ | ■ | | | | | | | | | | | | | | | | | | | |
| Selection of districts for PM in Luanda | | | | | | | | | | | | | | | ■ | | | | | | | | | | | | | | | | | | | | | | | | | |
| Approval of sampling frame | | | | | | | | | | | | | | | | ■ | | | | | | | | | | | | | | | | | | | | | | | | |
| Training of study staff | | | | | | | | | | | | | | | | | ■ | | | | | | | | | | | | | | | | | | | | | | | |
| Community informant interviews | | | | | | | | | | | | | | | | | | ■ | ■ | ■ | | | | | | | | | | | | | | | | | | | | |
| Data entry and compiling venue lists | | | | | | | | | | | | | | | | | | ■ | ■ | ■ | ■ | | | | | | | | | | | | | | | | | | | |
| Venue informant interviews | | | | | | | | | | | | | | | | | | | | | | ■ | ■ | ■ | | | | | | | | | | | | | | | | |
| Patron and worker interviews | | | | | | | | | | | | | | | | | | | | | | | | | ■ | ■ | ■ | ■ | | | | | | | | | | | | |
| Data cleaning and analysis | | | | | | | | | | | | | | | | | | | | | | | | | | | | ■ | ■ | ■ | ■ | | | | | | | | | |
| Drafting report from PM | | | | | | | | | | | | | | | | | | | | | | | | | | | | | | | ■ | ■ | ■ | ■ | | | | | | |
| Delivery of draft PM report | | | | | | | | | | | | | | | | | | | | | | | | | | | | | | | | | | | ■ | | | | | |
| Review of report draft and feedback | | | | | | | | | | | | | | | | | | | | | | | | | | | | | | | | | | | | ■ | | | | |
| Dissemination of results | | | | | | | | | | | | | | | | | | | | | | | | | | | | | | | | | | | | | ■ | ■ | | |

MRA: mapping readiness assessment; PM: programmatic mapping

## XII. APPENDICES

***APPENDIX 1. Mapping readiness assessment interview guide.***

**Programmatic Mapping Readiness Assessment for use with Key Populations**

*August 4, 2015*

*(Malawi, Haiti, Angola)*

**The Programmatic Mapping Readiness Assessment for use with Key Populations was compiled and edited by a team at the University of North Carolina-Chapel Hill Gillings School of Global Public Health:**

Kathryn Muessig

Sharon Weir

Kathryn Lancaster

Michael Herce

William M. Miller

Mamie Sackey Harris

**Sponsored by:**

Linkages across the Continuum of HIV Services for Key Populations Affected by HIV (LINKAGES), a USAID PEPFAR Program. Cooperative Agreement No. AID-OAA-A-14-00045

**Abbreviations**

| | |
|---|---|
| **FSW** | female sex workers |
| **KP** | key populations |
| **MRA** | mapping readiness assessment |
| **MSM** | men who have sex with men |
| **PLWH** | people living with HIV |
| **PM** | Programmatic Mapping |
| **PWID** | people who inject drugs |
| **SW** | sex workers |
| **TG** | Transgender persons |
| **VP** | vulnerable populations |

**Purpose**

The Programmatic Mapping Readiness Assessment tool (MRA) identifies the potential risks and benefits of conducting Programmatic Mapping (PM) and creates an action plan for addressing risk. The MRA focuses on the safety and well-being of key populations (KP) as individuals and as a group. The MRA guides discussions among program staff, steering committee members, and community leaders to assess the current situation of key populations and the risks and benefits of PM prior to initiating any data collection or mapping.

**What is Programmatic Mapping?**

PM is a collaboration of key population communities, service delivery providers and researchers to systematically identify and map the locations where people most at risk of acquiring and transmitting HIV can be reached. It is a tool to focus HIV prevention programing and resources where they have the greatest impact on the HIV epidemic. PM systematically identifies and maps sites (physical structures, public spaces, and internet-based) where KP congregate, estimates the number that could be reached at each site, and documents health and wellness services available near these locations. As described by the Global Fund, “the aim of this process is to improve program coverage by ensuring services are located where they are needed most.” PM can be conducted using a variety of methods including PLACE.[5,6] Estimates of the number of key populations at each site can be used to estimate the size of key populations, information that can be used to determine resource needs, program planning and resource allocation.

**Why conduct a Mapping Readiness Assessment?**

The primary purpose of conducting an MRA is to determine whether Programmatic Mapping can be implemented in a way that protects the safety, well-being and confidentiality of individuals and KP groups. Collecting information from vulnerable populations, even to improve programs, may pose unintended risks through breaches in confidentiality, misuse of spatial data, or drawing unwanted attention to KP groups.

Conducting an MRA helps achieve a safe and successful PM through a consultative process to gather information about risk, identify key people and places within the community and strengthen partnerships. These relationships will help guide the appropriate use of data generated by PM to best improve the availability of health services for KP. The MRA may determine that mapping poses too great a risk and should not be implemented.

**Whose voices should be included in a Mapping Readiness Assessment?**

The MRA  reflects conversations with a wide range of stakeholders including service delivery providers, law enforcement, legal experts, representatives from KP, leaders of KP organizations, experts in the field, general community members, religious leaders as appropriate, and local and regional leaders including government officials and those at local academic institutions who may already be working with KPs.

**Who should use this tool?**

The MRA is used by stakeholder(s) planning to conduct a PM study. It should be implemented in collaboration with the group(s) who will conduct the actual mapping, however it may rely on input, guidance and expertise of other individuals or institutions.

**How should this tool be used?**

The MRA assesses the appropriateness, readiness and safety of conducting PM within a community. Each community should review the MRA and adjust it as necessary to meet the particular needs and contexts of their community. The Linkages team will assist with this process.

The tool includes three guides:

1) Step-by-Step Guide to gather the information needed to identify collaborating partners, describe the KP that will be mapped, assess the local legal environment, and identify data safety and security procedures;

2) Stakeholder Question Guides to gather input from KP/KP representatives, healthcare and service providers and mapping team members; and

3) Summary Tables to describe each risk identified and create a plan for addressing this risk.

MRA activities do not need to be conducted in a specific order.  For example, the Summary Tables may be completed throughout the process of information gathering and then reviewed together at the end of the assessment.

After completing the MRA activities, a discussion should be held with key stakeholders and representatives of the community in order to arrive at a decision on whether, how and when to move forward with PM. Importantly, the MRA initiates a reflective process that should engage program staff and stakeholders at each stage of PM in order to best protect the safety and well-being of individuals and the community.

This list summarizes the activities required for conducting an MRA. They do not need to be conducted in a specific order. Information from Step 5 can inform the responses to all other steps. Question guides are provided on the following pages for each step. These can be adapted for your specific country context. The Linkages team can assist you in this process if you need help. ***To the extent possible, answers to these questions should first be gathered from existing data sources (e.g. USAID country office reports, Linkages Desk Review, Linkages preliminary site visits, other existing reports)***

1. Identify key community partners for programmatic mapping
2. Define and describe key populations (KP) to be mapped
3. Assess the legal environment for KP and mapping
4. Assess data safety and security considerations and capabilities
5. Gather perspectives from relevant stakeholders
    a. Gather perspectives from KP about mapping
    b. Gather perspectives from service and healthcare providers about mapping and using the information for program development
    c. Gather perspectives from programmatic mapping team to assess preparedness
6. Make a summary decision about the risks of PM
    a. Use the information collected in the activities above to create a comprehensive list of the risks of PM in your setting.
    b. Together with key stakeholders and representatives of the community, consider the information gathered in these activities to make a decision about whether to move forward with programmatic mapping.
    c. Create a step-by-step plan to address each risk identified.

**Step-by-Step Guide for Mapping Readiness Assessment**

The implementing partner identifies key stakeholders and community partners for Programmatic Mapping, gathers information from members/representatives of MSM, TG, FSW and PWID populations, and shares this information with a steering committee consisting of representatives of each of these groups.

The purpose of these consultations is to ensure that all key stakeholders are engaged in the discussion, to define and describe a typology of each key population that can be used in the mapping, to assess the legal environment for KP, and identify and assess safety concerns regarding mapping. At the end of the information gathering, a meeting should be held to review the evidence and determine whether to move ahead with the mapping. If the decision is made to move ahead, a plan should be developed to address risks. The key questions that should be answered are listed below. Some of these questions may be answered first using existing information sources. Additional information can then be added as you conduct stakeholder consultations (see three stakeholder consultation question guides that can be adapted as needed starting on page 13).

**1. Identify key community partners for Programmatic Mapping**

A. Who are the key community partners for PM? Identify one or more key partners in each of the following types of organizations (Fill-in the table below – use additional pages if needed).

| Community Partner Type | Name of organization | Contact person and information |
|---|---|---|
| Organizations led by and/or serving KP | | |
| Service delivery organizations and medical providers | | |
| Ministry of Health | | |
| Police and law enforcement representatives | | |
| Religious organizations | | |
| Traditional governance structures (i.e. Traditional Authorities, Village Headmen/women) | | |
| Other | | |

B. What new relationships will need to be built to bring the community to the point of readiness to support and participate in PM?

**2. Define and describe Key Populations to be mapped**

A. What is the typology of each KP? Can the typology be described in terms of locations, such as street-based, bar-based, brothel based? Which KP will be mapped?

B. What is the typology of KP sites? Which KP sites will be mapped?

C. What is already known about the characteristics and culture of sites where [sex work/injection drug use/MSM partner meeting] take place?

D. What is already known about the structure of [sex work/injection drug use/MSM partner meeting] in this setting that is relevant for PM activities? For example, are sex workers organized in tiers, types of sites, or types of exchange relationships?

E. What is the level of awareness of KP sites to the larger community? For example, are these venues known, tolerated, integrated or segregated within the community?

F. What is the current community-level stigma against KP?

G. What is the current level of empowerment and collective identity among KP? For example, are there existing KP activist or advocacy groups?

**3. Assess the legal environment for Key Populations and mapping**

A. What are the official local and national laws that affect KP?

    1) In practice, how are these laws applied?

    2) What penalties are enforced?

B. What protective laws are in place that affect KP?

    1) What laws protect people living with HIV from discrimination?

    2) What laws specify protections for KP?

C. What punitive laws are in place that affect KP?

1) What laws present obstacles to access to HIV services for KP?

2) What laws criminalize same sex sexual activities between consenting adults?

3) What laws deem sex work (prostitution) to be illegal?

4) What kind of compulsory treatment is available (e.g. methadone maintenance) for people who use drugs?

5) What offences carry the death penalty as a sentencing option? E.g. drug offences, same-sex behaviors.

D. What legal recourses are available to KP? For example Healthy Options Project Skopje (HOPS) in Macedonia provides legal services to female sex workers. It worked with sex workers to raise public awareness and fight police repression following the detention, forced HIV testing and public humiliation of sex workers in 2008. Similarly, the Asociación de Mujeres Meretrices de la Argentina (AMMAR) provides legal aid to female sex workers and has challenged police harassment and restrictions on where sex workers can work, partly through an alliance with labour unions.[13]

E. What political and legal trends and changes might occur in the coming years that would affect PM or KP? How could these changes alter the safety of PM for KP?

**4. Assess data safety and security considerations and capabilities**

A. What confidentiality agreements and data protection can realistically be put into place to ensure that maps and KP data are not used inappropriately?

B. Would mapping of public sites where a broader group of at-risk individuals could be reached be more appropriate or safer in this setting (as compared to focusing on KP sites)?

C. What is the plan to use the information gathered to improve services at the sites that are identified?

   1) What resources are or will be available to implement this plan?

   2) What additional resources are needed?

### 5. Gather perspectives from relevant stakeholders

#### a. Stakeholder Question Guide: Key Populations

**Goal:** Gather perspectives from members of each key population group and/or KP advocacy groups that will be mapped.

**Guidance:** Include both key opinion leaders and typical members. The following questions can be adapted and expanded for use in one-on-one consultancies or group settings. The exact methods used and questions asked should be chosen to best fit the local setting and practices. You may wish to circle or highlight the numbers of the questions you plan to ask before the conversation begins. You may also write-in additional questions you plan to ask.

Begin the interactions by providing a brief overview of the PM activities that are planned.

At the top of the notes page write down some basic information: assign a number to the conversation so you can keep track of it. Use this number on each page of your notes. Record the date and location where the conversation is taking place. Write down a description of who you are speaking with (include only their role, position or connection to KP, do not write down their name).

You may wish to take some brief notes below each question while you are conducting the interview to help you remember what the respondent says. As soon as possible once you have completed the interview take some additional time to expand your notes with more details from your memory of the conversation. Also take some time to write down follow-up questions you forgot to ask the person or new questions that come to mind based on the information you have learned.

All interviews should be documented within the MRA interview log book and stored in a locked cabinet. The MRA interview log book and hard-copy interview forms filled out by interviewer should be stored in a locked cabinet.

All responses and interview notes should be transcribed (typed) and translated on a UNC Project password protected computer. Both Chichewa and English transcriptions should be checked for quality and completeness by the interviewer and another LINKAGES Malawi staff. Transcripts should be saved locally and uploaded to the password protected UNC Project shared server daily.

**KP Consultation Notes (page 1 of 4)**

**Conversation#:**__________________

**Date:** ___________________________

**Location:** _______________________

**Role of respondent** (do not write down their name, just a description of their position, role, or reason why you are interviewing them):______________________________

___________________________________________________________________

___________________________________________________________________

**Interview questions for FSW (Adapt for MSM):**

1. At what types of places do FSWs socialize/ meet clients?

2. Which FSW do you think are not currently being reached by HIV/AIDS-related prevention and care services? Where do they meet clients?

3. Do you think providing HIV/AIDS-related prevention messaging/ SBCC and information regarding treatment and care services at places where they meet new clients is a good strategy?

4. What services are available that you would consider key population-friendly in this area?

5. What health and legal protection/advocacy services do FSW access? (e.g. legal representation, peer education, condoms, HIV testing and counseling, ART, PEP, PrEP, CD4/VL testing, STI screening, mental health services, family planning?)

**KP Consultation Notes (page 2 of 4)**

**Conversation#:____________________**

6. Where are these services located? (types of places)

7. What are the main barriers to accessing prevention and care services [HIV/STD testing/condoms/reproductive health care/HIV care]?

8. How do people in this community treat FSW?

9. The idea behind programmatic mapping is to identify where the key populations can be reached and make a map of these places so that programs can take services to these locations. Do you think that mapping these locations is a good idea? Why or why not?

10. Have you personally experienced any violence or harm from other people in this community or from law enforcement? What happened?

11. Have you witnessed or heard about these kinds of experiences from others?

12. What do you think about the mapping activities we have described?

13. What are your specific concerns about the mapping activities?

**KP Consultation Notes (page 3 of 4)**

**Conversation#:__________________**

14. What can we do to alleviate your concerns about mapping?

15. **MSM specific:** What do you think about mapping semi-public events like parties?

**Questions about HIV, CD4, and viral load testing at mapped locations:**

*Finger prick testing using just a few drops of blood can be done by trained fieldworkers in private rooms or unmarked clinic vans at the mapped locations. These tests can provide participants with their HIV status and current CD4 count within 30 minutes in the same visit and their viral load results within a few days/weeks.*

1. How easy do you think it would be to recruit [sex workers, MSM, people who inject drugs] at mapped locations to participate in finger prick testing?

2. What things would make it difficult to recruit people for this testing?

3. What things would make it easy to recruit people for testing?

4. What concerns would you have about this testing at mapped venues?

5. How would you feel about getting HIV and CD4 test results in this way?

**KP Consultation Notes (page 4 of 4)**

**Conversation#:__________________**

6. What risks to safety or privacy for [sex workers, MSM, people who inject drugs] do you think should be considered?

7. How willing would people be to provide their phone number or other contact information so that they could be provided their viral load test results a few days or weeks later?

8. What other suggestions would you have on how to give back viral load test results?

9. **MSM Specific:** What is the willingness of participants to recruit or refer their male partners?

10. **MSM Specific:** How feasible is couples-based enrollment and counseling?

11. **MSM Specific:** What is the willingness/ability to share test results with partners?

**Additional notes (use additional pages if necessary, add conversation # at top of each additional pate):**

**b. Stakeholder Question Guide: Healthcare and Service Providers**

**Goal:** Gather perspectives from healthcare and service providers about mapping

**Guidance:** Include both key opinion leaders and typical members. The following questions can be adapted and expanded for use in one-on-one consultancies or group settings. The exact methods used and questions asked should be chosen to best fit the local setting and practices.

Begin these interactions by providing a brief overview of the PM activities that are planned.

At the top of the notes page write down some basic information: assign a number to the conversation so you can keep track of it. Use this number on each page of your notes. Record the date and location where the conversation is taking place. Write down a description of who you are speaking with (include only their role, position or connection to KP, do not write down their name).

You may wish to take some brief notes below each question while you are conducting the interview to help you remember what the respondent says. As soon as possible once you have completed the interview take some additional time to expand your notes with more details from your memory of the conversation. Also take some time to write down follow-up questions you forgot to ask the person or new questions that come to mind based on the information you have learned.

All interviews should be documented within the MRA interview log book and stored in a locked cabinet.  The MRA interview log book and hard-copy interview forms filled out by interviewer should be stored in a locked cabinet.

All responses and interview notes should be transcribed (typed) and translated on a UNC Project password protected computer.  Both Chichewa and English transcriptions should be checked for quality and completeness by the interviewer and another LINKAGES Malawi staff.  Transcripts should be saved locally and uploaded to the password protected UNC Project shared server daily.

**Healthcare and Service Provider Consultation Notes (page 1 of 3)**

**Conversation#:**____________________

**Date:** ____________________________

**Location:** __________________________

**Role of respondent** (do not write down their name, just a description of their position, role, or reason why you are interviewing them):________________________________

___________________________________________________________________________

___________________________________________________________________________

**Interview questions:**

1. Could you describe any special arrangements you have to care for key populations?

2. What partnerships/relationships do you/your organization have with owners or managers of sites such as bars or clubs where key populations meet new sexual partners? What outreach activities have you done at these sites? (e.g. offer on-site HIV/STD testing, distribute condoms, etc.)

3. Could you describe any outreach efforts you have participated in that reached key populations?

4. What are your experiences providing HIV testing, condom distribution, or condom promotion at high-risk sites such as bars or clubs?

5. What kinds of challenges have you/your organization faced in conducting HIV prevention/care related activities in the past?

6. How did you/your organization overcome these challenges?

**Healthcare and Service Provider Consultation Notes (page 2 of 3)**

**Conversation#:___________________**

7. During a clinic visit, do you record whether a patient engages in high-risk behavior for HIV-transmission such as sex work, drug use, same sex behavior with men?

8. What do you think about the mapping activities we have described?

9. What are your specific concerns about the mapping activities?

10. What can we do to alleviate your concerns about mapping?

11. How could you/your organization use the information generated from mapping?

**Questions about HIV, CD4, and viral load testing at mapped locations:**

*Finger prick testing using just a few drops of blood can be done by trained fieldworkers in private rooms or unmarked clinic vans at the mapped locations. These tests can provide participants with their HIV status and current CD4 count within 30 minutes in the same visit and their viral load results within a few days/weeks.*

1. How easy do you think it would be to recruit [sex workers, MSM, people who inject drugs] at mapped locations to participate in finger prick testing?

2. What things would make it difficult to recruit people for this testing?

3. What things would make it easy to recruit people for this testing?

**Healthcare and Service Provider Consultation Notes (page 3 of 3)**

**Conversation#:__________________**

4. What concerns would you have about this testing at mapped venues?

5. What risks to safety or privacy for [sex workers, MSM, people who inject drugs] do you think should be considered?

6. How do you think this kind of testing might help your HIV prevention/care work?

7. What could be some benefits or challenges for health workers using this DBS tool?

8. Would you be willing to participate in the DBS collection at mapped locations?

9. What could be some benefits or challenges for Health workers participating in the assessment?

10. What procedures should be developed for these results to be used by healthcare and service provides to link [sex workers, MSM, people who inject drugs] to HIV care and treatment?

**Additional notes (use additional pages if necessary, add conversation # at top of each additional pate):**

### c. Stakeholder Question Guide: Programmatic Mapping Team

**Goal:** Gather perspectives from the programmatic mapping team about preparedness

**Guidance:** Include both key opinion leaders and typical members. The following questions can be adapted and expanded for use in one-on-one consultancies or group settings. The exact methods used and questions asked should be chosen to best fit the local setting and practices. Begin these interactions by providing a brief overview of the PM activities that are planned.

Begin these interactions by providing a brief overview of the PM activities that are planned.

At the top of the notes page write down some basic information: assign a number to the conversation so you can keep track of it. Use this number on each page of your notes. Record the date and location where the conversation is taking place. Write down a description of who you are speaking with (include only their role, position or connection to KP, do not write down their name).

You may wish to take some brief notes below each question while you are conducting the interview to help you remember what the respondent says. As soon as possible once you have completed the interview take some additional time to expand your notes with more details from your memory of the conversation. Also take some time to write down follow-up questions you forgot to ask the person or new questions that come to mind based on the information you have learned.

All interviews should be documented within the MRA interview log book and stored in a locked cabinet. The MRA interview log book and hard-copy interview forms filled out by interviewer should be stored in a locked cabinet.

All responses and interview notes should be transcribed (typed) and translated on a UNC Project password protected computer. Both Chichewa and English transcriptions should be checked for quality and completeness by the interviewer and another LINKAGES Malawi staff. Transcripts should be saved locally and uploaded to the password protected UNC Project shared server daily.

**Programmatic Mapping Team Consultation Notes (page 1 of 3)**

**Conversation#:**__________________

**Date:** __________________________

**Location:** _______________________

**Role of respondent** (do not write down their name, just a description of their position, role, or reason why you are interviewing them):______________________________

___________________________________________________________________

___________________________________________________________________

**Interview questions:**

1. What will your role be in the mapping activities?

2. What specific jobs will this entail?

3. What steps do you need to take for your safety and comfort while conducting this job?

4. What are your specific concerns about the mapping activities?

5. What concerns have you heard from others in the community?

6. What can we do to alleviate your concerns about mapping?

7. What value/benefit do you think the information from mapping could provide?

**Programmatic Mapping Team Consultation Notes (page 2 of 3)**

**Conversation#:__________________**

8. What do you think are the most important things we can do to protect the safety and security of those who will be affected by these mapping activities?

**Questions about HIV, CD4, and viral load testing at mapped locations:**

*Finger prick testing using just a few drops of blood can be done by trained fieldworkers in private rooms or unmarked clinic vans at the mapped locations. These tests can provide participants with their HIV status and current CD4 count within 30 minutes in the same visit and their viral load results within a few days/weeks.*

1. How easy do you think it would be to recruit [sex workers, MSM, people who inject drugs] at mapped locations to participate in finger prick testing?

2. What things would make it difficult to recruit people for this testing?

3. What things would make it easy to recruit people for this testing?

4. What concerns would you have about doing this testing at mapped venues?

5. What concerns would you have about providing people with their test results at mapped venues?

6. What risks to safety or privacy for [sex workers, MSM, people who inject drugs] do you think should be considered?

7. How difficult do you think it would be to re-contact people a few days or weeks later to share their viral load results with them?

**Programmatic Mapping Team Consultation Notes (page 3 of 3)**

**Conversation#:__________________**

8. How comfortable would you be conducting this testing?

9. What risks to safety for you should be considered with conducting this testing?

10. What could be some benefits or challenges for health workers using this DBS tool?

**Additional notes (use additional pages if necessary, add conversation # at top of each additional page):**

### 6. Make a Summary Decision about the risks of Programmatic Mapping

#### a. Create a comprehensive list of the risks of Programmatic Mapping

Use the form of the following page (Summary of PM Risks) to create a list of the risks of PM. Consider the information gathered in these activities to make a decision about whether and how to move forward with PM in order to protect the safety and security of KP. These questions will help guide your list creation, discussion and decision.

- Could consensus among KP/KP representatives be reached regarding the risks and benefits of PM?
- How likely is the data generated from PM to be used?
- What risks of PM were identified?
- Who do these risks most affect? *(e.g. brothel-based SW, street-based SW, mapping team, MSM, etc.)*
- How serious are these risks? *(e.g. a "low" risk might include individual discomfort or embarrassment or minor financial loss, a "moderate" risk might include discrimination or a major financial loss, a "high" risk might include high likelihood of violence or arrest)*
- How likely are they to happen? *(unlikely, possible, very likely, certain)*
- What precautions could be taken in order to ameliorate or minimize these risks? *(list multiple possible steps)*
- In summary, is it possible to implement PM safely in the country?
- In summary, is it possible and acceptable to conduct finger stick testing for HIV, CD4 count and viral load at the mapped venues?

**Summary of PM risks**

| Description of risk | Who is affected? | Seriousness of risk | Likelihood of risk | Possible precautions to minimize risks |
|---|---|---|---|---|
| | | | | |
| | | | | |
| | | | | |
| | | | | |
| | | | | |
| | | | | |
| | | | | |
| | | | | |
| | | | | |
| | | | | |
| | | | | |
| | | | | |
| | | | | |
| | | | | |
| | | | | |
| | | | | |
| | | | | |
| | | | | |
| | | | | |
| | | | | |
| | | | | |
| | | | | |

**b. Make a decision about whether to move forward with Programmatic Mapping**

Together with key stakeholders and representatives of the community consider the information gathered in the five activities above and summarized in the risk tool to make a decision about whether to move forward with programmatic mapping. This decision should be made following an appropriate local procedure such as a community forum or vote.

**c. Create a step-by-step Action Plan to address each risk identified**

**If the decision is made to move forward with PM:**

- What action steps will be taken to address each risk?
- Who will be responsible for completing these actions?
- When will these actions be completed?

- **Action Plan**

| Risk | Action steps | Person(s) responsible | Complete by date | Completed? (Y/N) |
|---|---|---|---|---|
| | | | | |
| | | | | |
| | | | | |
| | | | | |
| | | | | |
| | | | | |
| | | | | |
| | | | | |
| | | | | |
| | | | | |

**A. RDS SURVEY METHODS**

A.1. How well do the MSM, transgender, and male sex worker populations interact with each other in _______ (i.e. frequent the same venue, have the same friends, etc.)?

A.2. Do you think that a recruitment system where MSM recruit other MSM using coupons is feasible in _______?

A.3. Please review the information on the coupon. Is it easy to understand? Should we be offering appointments? Are we missing any important information that really needs to be there to make it easy for participants to come see us?

A.4. _______ has agreed to serve as the study site. Is this a good location or should the study be moved to another location?

A.5. What should the hours of operation be during the data collection phase?

A.6. We want to compensate participants for their time but we don't want to offer too much. We don't want men to pretend they have sex with men so that they can join the study for the study incentives.

The first study visit takes about 1 hour. At the end of visit 1, participants will be given incentives equivalent to 900 Kwanzas in the form of mobile phone credit for participating in the survey. Is this about the right amount? Are the items appropriate? Are there other items that MSM would like to have for participating in this survey.

Probe: If no, what amount would be more appropriate? Is it more or less?

A.7. What would motivate MSM to participate in our survey? What would prevent MSM from participating in our survey?

A.8. How many sex workers do you know and who know you and live in this city?

A.9. How many men who have sex with men do you know and who know you and live in this city?

**By knowing someone we mean that you know their names and have a way to contact them.**

**Men who have sex with men: all men who are openly homosexual or gay or bisexual, or living in the closet. (not transgender)**

Observation notes: is the question easily understood, does the respondent have problems estimating a number?

A.10. How many transgender people do you know and who know you and live in this city?

**Transgender:** people who were assigned one sex at birth but who feel that this is a false or incomplete description of themselves

***APPENDIX 2. Form A: Community Informant Interview Questionnaire***

## FORM A: Instructions to Interviewer for Community Informant Interviews

1. READ: Hello. My name is <full name> and I am working with <implementing agency and collaborating groups> on a study that will improve health programs in this area. I would like to ask you some questions about where programs are needed. This includes asking you about places around here where people meet new sexual partners and places where people who inject drugs congregate. People who have many sexual partners or inject drugs are more at risk for some infections. I can offer you this Fact Sheet that has more information about the study. This should take 15 minutes.
2. OFFER FORM A2 FACT SHEET. ANSWER QUESTIONS.
3. CONFIRM THAT RESPONDENT IS AGE 18 OR OLDER. IF YOUNGER THAN 18, STOP AND FIND ANOTHER RESPONDENT.
4. ASK: Are you willing to answer some questions? IF RESPONDENT REFUSES, FIND ANOTHER RESPONDENT.
5. FILL IN FORM A3 FOR EACH COMMUNITY INFORMANT WHO CONSENTS.
6. ASK: We want to know about public places in this area that may benefit from visits from health workers.
    - Could you tell me about public places in this area where people go to meet new sex partners? We are interested in public sites and events and internet sites. We are not interested in private homes.
    - Can you tell me about public places where people who use drugs could be reached with a health program? You can tell me up to 5 sites.
    - Can you tell me about any other public sites where sex workers solicit clients? Or clients look for sex workers?
    - Can you tell me about any other public sites where gay or bisexual men or any other men who have sex with men congregate?
    - Can you tell me about any other public sites where transgender people congregate?
7. FOR EACH QUESTION ANSWERED AFFIRMATIVELY, FILL IN 1 SITE AND EVENT REPORT FORM A4 FOR EACH SITE NAMED.
8. LIMIT THE PLACES TO SITES IN THE ZONE WHERE THE INTERVIEW IS BEING CONDUCTED.
9. RECORD NUMBER OF SITES OR EVENTS NAMED BY THE COMMUNITY INFORMANT ON FORM A3 QUESTIONS 6A, 6B, AND 6C.

QUESTION-BY-QUESTION INSTRUCTIONS FOR FORM A3

1 Date is date of interview. Use Day / Month / Year format. Example: 27 / 05 / 14 for May 27, 2014.

2 Use geographic codes, indicating province, district, and zone where interview is conducted.

3 This is the interviewer's ID code. Letters or numbers can be used. Every interviewer should have an ID code. For Example Lisa Smith could be LS.

4 This is a consecutive number that starts at 1 and continues to 2, 3, 4, etc. for each community informant interviewed by a particular interviewer. Each interviewer begins at 1 every day. If an interviewer does 15 interviews in a day the first community informant number will be 1 and the last one 15.

5 Type of community informant. Must be adapted to situation in each country. Enter the one best code to describe the type.

6 Categorized by type of site, the total number of unique sites given by the community informant (6A is the total number of physical sites the informant named, 6B is the number of websites, and 6C is the number of events).

## FORM A: Characteristics of Community Informant

| FORM A COMMUNITY INFORMANT CHARACTERISTICS FORM FILL OUT 1-6 FOR EACH INFORMANT. | | | |
|---|---|---|---|
| **1.** Date: Day ☐☐ Month ☐☐ Year ☐☐ | **2.** Geographic Codes **2A.** Province: ☐☐ **2B.** District: ☐☐ **2C.** Zone: ☐☐ | | |
| **3.** Interviewer Code: ☐☐ | **4.** Community Informant Number: ☐☐☐ | **5.** Type of Informant USE CODES: ☐☐ | |
| **6.** Number of physical sites, websites, and events named by this informant | **6A.** Physical Sites: ☐☐ | **6B.** Websites: ☐☐ | **6C.** Events: ☐☐ |

| **Community Informant Type Codes**<br>USE FOR QUESTION 5. | **If Not Key Population:** | | |
|---|---|---|---|
| | Taxi driver 7 | Nurse 16 | Mechanics, petrol attendants 27 |
| | Truck driver 8 | Peer educator 17 | Hairdresser 28 |
| **If Key Population:** | Shebeen owner 9 | Community health worker 18 | Community leader 29 |
| Trans woman (Male to Female) 1 | Bar or tavern owner 10 | Youth in school 19 | Political leader 30 |
| Trans man (Female to Male) 2 | Barmaid, waiter, bar worker 11 | Youth out of school 20 | Traditional leader 31 |
| Person who injects drugs 3 | Individual socialising at site 12 | Teacher 21 | Ward counsellor 32 |
| Man who has sex with men 4 | Bottle store/brewery drop off 13 | SAPS (police) 22 | Car guard 33 |
| Man who sells sex for money 5 | CBO/NGO staff 14 | Security guard 23 | Other man 34 |
| Woman who sells sex for money 6 | Military 15 | Trader/business 24 | Other woman 35 |
| | | Hawker/ street vendor 25 | Other 36 |
| | | Unemployed 26 | |

## Data Entry Template for Form A

Below are sample column headings to use in an Excel spreadsheet when entering data collected using Form A3.

Use 1 row per Form A3 (i.e., one row for each community informant interviewed). See example Below. Each column is a variable:

- Int_Code=Q3  Interviewer code
- CI_Num= Q4 Community informant Number
- CI_type= Q5 Community Informant Type
- Num_sites= Q6a Number of physical sites named by this community informant
- Num_websites=Q6B Number of websites named
- Num_events= Q6C Number of events named

| | A | B | C | D | E | F | G | H | I | G |
|---|---|---|---|---|---|---|---|---|---|---|
| 1 | DATE | PROVINCE | DISTRICT | ZONE | INT_CODE | CI_NUM | CI_TYPE | NUM_PHYSSITES | NUM_WEBSITES | NUM_EVENTS |
| 2 | 30/2/2016 | Eastern | Orange | 3 | 2 | 23 | 5 | 2 | 3 | 1 |
| 3 | | | | | | | | | | |
| 4 | | | | | | | | | | |

## FORM A2: Characteristics of Site or Event

NUMBER OF CI REPORTING: [ ][ ]

**FORM A2 SITE AND EVENT IDENTIFICATION FORM (1 FORM PER SITE OR EVENT NAMED)**

**1.** Date: Day [ ][ ] Month [ ][ ] Year [ ][ ]

**2.** Geographic Codes **2A.** Province: [ ][ ] **2B.** District: [ ][ ] **2C.** Zone: [ ][ ]

**3.** Interviewer Code: [ ][ ]

**4.** Community Informant Number: [ ][ ][ ]

**5.** Site Name:

**6.** Type of Site USE CODES BELOW: [ ][ ]

**7.** Address/How to Find the Place:

**8.** Landmark:

**9.** Busiest Day of Week at Site:
CIRCLE ONE DAY.

| | |
|---|---|
| Monday | 1 |
| Tuesday | 2 |
| Wednesday | 3 |
| Thursday | 4 |
| Friday | 5 |
| Saturday | 6 |
| Sunday | 7 |

**10.** Busiest Time of Day:
CIRCLE ONE TIME.

| | |
|---|---|
| Early Morning | 1 |
| Late Morning/Lunch | 2 |
| Afternoon | 3 |
| Evening/Night | 4 |
| Whole Day/24 Hours | 5 |
| Day Time Work Hours | 6 |

**11.** Any of these populations at site?

| | | Yes | No | Don't know |
|---|---|---|---|---|
| **A.** | Women who sell sex for money | 1 | 2 | 9 |
| **B.** | Women who sell sex for goods/favors | 1 | 2 | 9 |
| **C.** | Men who sell sex for money | 1 | 2 | 9 |
| **D.** | People who inject drugs | 1 | 2 | 9 |
| **E.** | Men who have sex with men | 1 | 2 | 9 |
| **F.** | Transgender, transvestite, transsexual | 1 | 2 | 9 |

**12.** How many people are at the site at a busy time?

| | |
|---|---|
| Fewer than 30 people | 1 |
| 30-100 people | 2 |
| More than 100 people | 3 |

**Site Type Codes**
USE FOR QUESTION 6.

| | | | | | | | |
|---|---|---|---|---|---|---|---|
| Formal bar | 1 | Hotel | 7 | Kiosk | 15 | OTHER: | |
| Informal bar | 2 | Small guest house | 8 | Store | 16 | Cultural event | 23 |
| Night/Exotic club | 3 | Street sex worker site | 9 | Market | 17 | Party event | 24 |
| Massage parlor | 4 | Taxi stand/Bus stop | 10 | Shopping Centre/Mall | 18 | Other event | 25 |
| Brothel | 5 | Other street site | 11 | Fast food/Restaurant | 19 | Website | 26 |
| Truck stop | 6 | Beach/Creek | 12 | Church/Temple/Mosque | 20 | Escort service | 27 |
| | | Park/Plaza | 13 | Campus/School | 21 | Phone | 28 |
| | | Construction site | 14 | Tourist attraction | 22 | | |

## Data Entry Template for Form A2

1. After the community informant interviews have been completed for a zone, the Form A4s should be sorted by site with each stack containing the reports for one site. Because each community informant may name up to 10 sites or events, 500 community informants may generate as many as 5000 Form A4s. For example, if 20 Community Informants reported Joe's Bar, then there will be 20 Form A4s for Joe's Bar. There may be hundreds of sites named, so sorting usually requires a large room with tables.
2. After the Form A4s are sorted into stacks, the interviewing team should review each stack, for example, all of the Form A4a for Joe's Bar. Some of the reports will differ. Perhaps some people said the site has men who have sex with men and others did not. Only one report per site will be entered into the Excel spreadsheet. If the reports differ, edit one of the reports to make it the most correct version. Use a red pen to edit the report. The most common edits are usually to combine information from various reports about the address or landmarks. Another common edit is to reflect the presence of key populations at the site. If ANY of the community informants reported that a type of key population visits the site, then circle 'yes' for this type of key population in questions 11A-11F on the version that will be entered into Excel. So if one community informant said that the Joe's Bar has men who have sex with men, even if other community informants did not report that men who have sex with men are present there, circle 'yes' for men who have sex with men in the report from Joe's Bar that will be entered into the Excel spreadsheet.
3. For each site, count how many Community Informants (CI) reported the site. This will be the number of Form A4s in the stack. For each site, on the Form A4 that will be entered into the Excel spreadsheet, write the total number of CIs who reported the site in the shaded box labeled: "Number of CI Reporting" in the top right corner.
4. Create one Excel spreadsheet for each zone. Use the template below to set up each zone's spreadsheet.
5. After the corrected Form A4 for each site in the zone has been entered, sort the list by the name of the site and then enter a sequential Site ID in column E. The first three digits should indicate the province, district, and zone. Below is an example where the province was '3', the district was '2', and the zone was '4'. The last three digits should be sequential, beginning with 001. See example. The Site ID is the unique identifier for the site, and it will remain with this site for the remainder of the data collection process. For example, any further surveys conducted at Joe's Bar will be labeled with the site ID 324001.
6. In column P, be sure to enter the number of community informants who reported the site.

| | A | B | C | D | E | F | G | H | I | G |
|---|---|---|---|---|---|---|---|---|---|---|
| 1 | DATE | PROVINCE | DISTRICT | ZONE | Site_ID | Site_Name | Site_Type | Address | Landmark | Busiest_Day |
| 2 | 30/2/2016 | 3 | 2 | 4 | 324001 | Joes Bar | 1 | 20 Main Street | By Library | 6 |
| 3 | 30/2/2016 | 3 | 2 | 4 | 324002 | His Bar | 1 | 60 Main Street | By Grocery | 6 |
| 4 | 30/2/2016 | 3 | 2 | 4 | 324003 | Zahra's | 8 | 400 Elm Street | By Truckstop | 6 |

| | H | I | J | K | L | M | N | O | P |
|---|---|---|---|---|---|---|---|---|---|
| 1 | Busy_Time | FSW_11a | Goods_11b | MSW_11c | PWID_11d | MSM_11e | TG_11f | Size | Number_CI_Reports |
| 2 | 4 | 1 | 2 | 1 | 2 | 1 | 2 | 1 | 2 |
| 3 | 4 | 1 | 2 | 9 | 2 | 2 | 2 | 3 | 9 |

*APPENDIX 3. Form B Venue Informant Interview Questionnaire*

## FORM B: Instructions to Interviewer and Social Mobilizer for Site Verification

1. Social Mobilizer and Interviewer go to site. Inside, the Social Mobilizer identifies the Site Informant to interview. The Site Informant should be a member of a key population who is very knowledgeable about the site. If a key population member is not present, identify the most knowledgeable person at the site. The social mobilizer and the interviewer introduce themselves by name and show their permission letters and identification.
2. INTERVIEWER FILLS OUT PART I BEFORE INITIATING THE SITE INFORMANT INTERVIEW.
3. INTERVIEWER: Hello. My name is < > and I am working with < > on a study coordinated by < > that will improve HIV prevention programs. I would like to ask you some questions about this site and the people who come here. I can offer you this information sheet that has more information about the study. This should take about 20-30 minutes.
4. OFFER FORM B2 FACT SHEET. ANSWER QUESTIONS.
5. CONFIRM THAT RESPONDENT IS AGE 18 OR OLDER. IF YOUNGER THAN 18, STOP AND FIND ANOTHER RESPONDENT.
6. ASK: Are you willing to answer some questions? IF RESPONDENT REFUSES, FIND ANOTHER RESPONDENT.
7. FILL IN FORM B3.

QUESTION-BY-QUESTION INSTRUCTIONS FOR FORM B3

Q1. Date is date of the interview. Day / Month / Year Format. Example: 27 / 05 / 14 for May 27, 2014.

Q2. This is the interviewer's ID code.

Q3. This is the type of social mobilizer present.

Q4. Enter the site's geographic code for province, district, and zone, e.g. for Joe's Bar, province '3', district '2', and zone '4'.

Q5. Enter the Site ID number that was assigned to this site. For Joe's Bar, you would enter 324001. It is important that the same site ID number is entered here as was assigned in the Excel spreadsheet with the Form A4 data.

1. Date is date of interview. Day / Month / Year Format. Example: 27 / 05 / 14 for May 27, 2014.
2. This is the interviewer's ID code. Every interviewer should have an ID code. For Example Lisa Smith could be LS.
4. Enter the site's geographic codes for province, district, and zone.
5. This is the unique number that has been assigned to this site following collation of Form A4s.
7. This is the information for the site that was given in Form A4.
8. This is the corrected information for the site as determined during verification of the site.

## FORM B: Site Verification Form

| PART I – COMPLETE BEFORE INTERVIEW | | |
|---|---|---|
| **NO.** | **QUESTION** | **RESPONSE** |
| Q1 | Date: | Day ☐☐ Month ☐☐ Year ☐☐ |
| Q2 | Interviewer Code: | Interviewer Code: ☐☐ |
| Q3 | Type of social mobilizer present: | No social mobilizer present during site visit 0<br>Sex worker 1<br>MSM 2<br>Trans woman (Male to female) 3<br>Trans man: (Female to male) 4<br>Person who injects drugs 5<br>Other 6 |
| Q4 A-C | Geographic Code of Site: | **A.** Province ☐☐ **B.** District ☐☐ **C.** Zone ☐☐ |
| Q5 | Site ID Number: | Site Number: ________________________________ |
| | Site Name Given by Community Informant:<br>IF WEBSITE, INDICATE EXACT WEBSITE. | |
| Q6A | **A.** Verified Site Name: | |
| | Site Address Given by Community Informant: | |
| Q6B | **B.** Verified Site Address: | |
| | Landmark Given by Community Informant: | |
| Q6C | **C.** Correct Landmark: | |

| | | |
|---|---|---|
| Q7<br>A-C | Was the site found?<br><br>IF "OTHER" IS SELECTED FOR PART A, EXPLAIN.<br><br>IF SITE IS A DUPLICATE LOCATION (FOR EXAMPLE SITE WAS ALREADY VERIFIED UNDER A DIFFERENT NAME), WRITE THE NUMBER FOR THE OTHER SITE THAT THIS SITE DUPLICATES IN Q7C. | **A.** Location not found 0<br>Location found, interview conducted 1<br>Location closed 2<br>Location found, informant declined 3<br>Not a duplicate location, but other 4<br>Duplicate location 5<br>**B.** IF OTHER (Q7A = 4), EXPLAIN:______________________________<br>**C.** IF DUPLICATE (Q7A = 5), OTHER SITE NUMBER:_____________ |

**PART I – CONTINUED**

| NO. | QUESTION | RESPONSE |
|---|---|---|
| Q8<br>A-B | GPS Coordinates:<br>USE THE GPS UNIT. WRITE COORDINATES HERE.<br>FOR LONGITUDE: ALSO INDICATE WHETHER WEST OR EAST.<br>FOR LATITUDE: ALSO INDICATE WHETHER NORTH OR SOUTH. | **A.** Longitude: ___________________________<br><br>**B.** Latitude: ______________________________ |
| Q9A | Type of Site (USE CODES BELOW)<br>CODES 1 -22 ARE FOR TYPES OF VENUES.<br>CODES 23-25 ARE FOR EVENTS. IF A WEBSITE, CIRCLE 26. | ENTER 1 CODE:<br>**A.** ☐☐ |

| | | | |
|---|---|---|---|
| | Formal bar 1 | Other street site 11 | Campus/School 21 |
| | Informal bar 2 | Beach/Creek 12 | Tourist attraction 22 |
| | Night/Exotic club 3 | Park/Plaza 13 | |
| | Massage parlor 4 | Construction site 14 | OTHER: |
| | Brothel 5 | Kiosk 15 | Cultural event 23 |
| | Truck stop 6 | Store 16 | Party event 24 |
| | Hotel 7 | Market 17 | Other event 25 |
| | Small guest house 8 | Shopping Centre/Mall 18 | Website 26 |
| | Street sex worker site 9 | Fast food/Restaurant 19 | Escort service 27 |
| | Taxi stand/Bus stop 10 | Church/Temple/Mosque 20 | Phone 28 |

| | | | | |
|---|---|---|---|---|
| Q9<br>B-I | Physical characteristics of the site:<br>CIRCLE ONE ANSWER (1 OR 2) FOR EACH CHARACTERISTIC, A-H. | | Yes | No |
| | | **B.** Electricity available | 1 | 2 |
| | | **C.** Tap water available | 1 | 2 |
| | | **D.** Walls and ceiling | 1 | 2 |

| | | | | |
|---|---|---|---|---|
| | | **E.** Inside toilet | 1 | 2 |
| | | **F.** Beds on site | 1 | 2 |
| | | **G.** Videos shown onsite | 1 | 2 |
| | | **H.** TV onsite | 1 | 2 |
| | | **I.** Bar for alcohol sales onsite | 1 | 2 |
| Q9<br>J-N | Evidence of HIV/AIDS prevention activities visible at the venue:<br>CIRCLE ONE ANSWER (1 OR 2) FOR EACH PREVENTION ACTIVITY ITEM, A-E. | | Yes | No |
| | | **J.** HIV/AIDS posters displayed | 1 | 2 |
| | | **K.** Condom promotion posters | 1 | 2 |
| | | **L.** Condoms visible | 1 | 2 |
| | | **M.** Lube visible | 1 | 2 |
| | | **N.** Sterile needles/syringes visible | 1 | 2 |

**PART II – BEGIN INTERVIEW**

READ: Hello. My name is < > and I am working with < > on a study coordinated by < > that will improve HIV prevention programs. I would like to ask you some questions about this site. I can offer you this information sheet that has more information about the study. This should take about 30-40 minutes.

| NO. | QUESTION | RESPONSE |
|---|---|---|
| Q10<br>A-C | INSTRUCTIONS TO THE INTERVIEWER (DO NOT READ ALOUD):<br>**A.** DID YOU READ THE INFORMATION SHEET TO THE RESPONDENT?<br>**B.** DID YOU READ THE CONSENT FORM TO RESPONDENT IN LANGUAGE RESPONDENT UNDERSTANDS?<br>**C.** HAVE YOU ASKED THE RESPONDENT IF HE/SHE HAS QUESTIONS, AND HAVE YOU ANSWERED THOSE QUESTIONS? | Yes No<br>**A.** 1 2<br>**B.** 1 2<br>**C.** 1 2 |
| Q11 | Are you willing to answer the questions I will ask you? | Yes No<br>1 2 |
| Q12 | What is your age? IF RESPONDENT IS YOUNGER THAN 18, STOP INTERVIEW AND FIND ANOTHER. | WRITE AGE IN YEARS: [ ][ ] |
| Q13 | Do you work here? | Yes No<br>1 2 |
| Q14 | Were you born as a male or female? | MALE 1<br>FEMALE 2 |

| | | | |
|---|---|---|---|
| Q15 | Do you currently identify more as a male or more as a female? | | MALE 1<br>FEMALE 2 |
| **SECTION B. QUESTIONS ABOUT THE SITE** | | | |
| B1<br>A-G | **A-G.** During a typical week, are these days busy? | Yes No<br>**A.** Monday 1 2<br>**B.** Tuesday 1 2<br>**C.** Wednesday 1 2<br>**D.** Thursday 1 2<br>**E.** Friday 1 2<br>**F.** Saturday 1 2<br>**G.** Sunday 1 2 | |
| B2 | On a busy day, what hours are the busiest?<br>CIRCLE ONLY ONE CHOICE FOR BUSIEST TIME. | Early morning 1<br>Late morning/Lunch time 2<br>Afternoon 3<br>Evening/night 4<br>Whole day/24 hours 5<br>Day time work hours 6 | |
| **NO.** | **QUESTION** | | **RESPONSE** |
| B3<br>A-B | **A.** Did you come here on (MOST RECENT BUSY DAY FROM B1)?<br>**B.** On that day, how many other public places did you visit to socialize, drink alcohol, or look for a person to have sex with? | | Yes<br>No<br>**A.** 1 2<br>**B.** NUMBER OF SITES<br>:_____ |

<table>
<tr><td>B4<br>A-J</td><td>I would like to talk to you about the characteristics of people who come to this place at busy times.<br>In your opinion do…<br><br>READ LIST.<br><br>CIRCLE ONE RESPONSE FOR EACH QUESTION, A-J.<br>CIRCLE 1 IF YES, 2 IF NO, OR 9 IF THE RESPONDENT DOES NOT KNOW.</td><td colspan="2">Yes No Don't Know<br><br>A. People meet new sex partners here? 1 2 9<br>B. Women who sell sex come here to find customers? 1 2 9<br>C. Men who sell sex come here to find customers? 1 2 9<br>D. Any male staff meet new sex partners here? 1 2 9<br>E. Any female staff meet new sex partners here? 1 2 9<br>F. People have sex on site? 1 2 9<br>G. Men meet male sex partners here? 1 2 9<br>H. Young girls aged 15 – 17 come here? 1 2 9<br>I. People who inject drugs come here? 1 2 9<br>J. Is there someone onsite who helps people find new sex partners? 1 2 9</td></tr>
<tr><td>B5</td><td colspan="2">How many women work at this site over the course of a busy day and night? That is they are employed by the site or they are self-employed and work here.</td><td>NUMBER: ________</td></tr>
</table>

| | | |
|---|---|---|
| B6<br>A-B | Some women exchange sex for money and some exchange sex for goods or favors. Goods or favors includes housing, transportation, food or gifts.<br><br>**A.** Of the <u>(NUMBER FROM B5)</u> women who work here, how many do you estimate occasionally or regularly exchange sex for money?<br><br>**B.** Of the <u>(NUMBER FROM B5)</u> women who work here, how many do you estimate exchange sex for goods or favors? | **A.** NUMBER: _______<br><br>**B.** NUMBER: _______ |
| **NO.** | **QUESTION** | **RESPONSE** |
| B7 | On a busy day, how many women aged 18 and older come here as patrons (to socialize, drink alcohol, etc.) over the course of the entire day and night?<br><br>READ OPTIONS. CIRCLE ONE CODE BETWEEN 0 AND 13 THAT INDICATES THE CORRECT RANGE. | CIRCLE ONE CODE BETWEEN 1 AND 13:<br>0 ... 1<br>1-14 ... 2<br>15-29 ... 3<br>30-49 ... 4<br>50-99 ... 5<br>100-149 ... 6<br>150-199 ... 7<br>200-299 ... 8<br>300-399 ... 9<br>400-499 ... 10<br>500-999 ... 11<br>1000-1999 ... 12<br>Over 2000 ... 13 |
| B8 | You have given me a range. Can you be more specific now and provide your best guess (estimate)? IF NONE, ENTER 0. IF THE NUMBER DOES NOT FALL INTO THE RANGE GIVEN FOR B7 PROBE FOR AN ACCURATE RESPONSE. | NUMBER: _______ |
| B9 | On a busy night, how many of the _____(NUMBER FROM B8) female patrons who come here do you think exchange sex for money? IF NONE, ENTER 0. | NUMBER: _______ |
| B10 | On a busy night, how many of the ______(NUMBER FROM B8) female patrons who come here exchange sex for goods or favors? IF NONE, ENTER 0. | NUMBER: _______ |

| | | |
|---|---|---|
| B11 | On a busy day, how many men will be here over the course of the entire day and night?<br><br>READ OPTIONS. CIRCLE ONE CODE BETWEEN 0 AND 13 THAT INDICATES THE CORRECT RANGE. | CIRCLE ONE CODE BETWEEN 1 AND 13:<br>0 1<br>1-14 2<br>15-29 3<br>30-49 4<br>50-99 5<br>100-149 6<br>150-199 7<br>200-299 8<br>300-399 9<br>400-499 10<br>500-999 11<br>1000-1999 12<br>Over 2000 13 |
| **NO.** | **QUESTION** | **RESPONSE** |
| B12 | You have given me a range. Can you be more specific now and provide your best guess (estimate)? IF NONE, ENTER 0. | NUMBER: ______ |
| B13 | On a busy night, how many of the _______ (NUMBER FROM B12) men aged 18 or older who come here do you think exchange sex for money?<br>IF NONE, ENTER 0. | NUMBER: _______<br>. |
| B14 | On a busy night, how many of the ______(NUMBER FROM B12) men aged 18 or older who come here are men who have sex with other men? These men can be gay, bisexual or straight. IF NONE, ENTER 0. | NUMBER: _______ |
| B15 | How many transgender people, transsexuals or transvestites do you estimate come here on a busy night?<br>IF NONE ENTER ZERO. IF THE PERSON DOES NOT KNOW CIRCLE 999.<br>SOME PEOPLE ARE BORN MALE, BUT LATER ON IN THEIR LIVES MIGHT NOT SEE THEMSELVES AS A MAN. THEY MAY FEEL MORE COMFORTABLE AS A WOMAN. | NUMBER:___________<br>DON'T KNOW 999 |

| NO. | QUESTION | RESPONSE |
|---|---|---|
| B16 | Some people inject drugs illegally. These people need health care services. About how many of the men who come here on a busy day do you think may have injected drugs in the past year? Would you say…. READ OPTIONS | NONE 0<br>VERY FEW 1<br>FEW 2<br>LESS THAN HALF 3<br>ABOUT HALF 4<br>MORE THAN HALF 5<br>ALMOST ALL 6<br>ALL 7<br>DON'T KNOW 9 |
| B17 | About how many women who come here on a busy day do you think may have injected drugs in the past year? Would you say…READ OPTIONS. | NONE 0<br>VERY FEW 1<br>FEW 2<br>LESS THAN HALF 3<br>ABOUT HALF 4<br>MORE THAN HALF 5<br>ALMOST ALL 6<br>ALL 7<br>DON'T KNOW 9 |
| B18 | Which types of activities take place here at a busy time?<br><br>READ LIST.<br>CIRCLE ONE RESPONSE (1 OR 2) FOR EACH QUESTION, A-E. | Yes No<br>**A.** Alcohol consumed 1 2<br>**B.** People buy food to eat 1 2<br>**C.** People socialize for 1 hour or more 1 2<br>**D.** People watch sports events 1 2<br>**E.** Other 1 2 |
| B19 | Can I get a male condom here?<br>IF YES: Can I see one? | Yes, male condoms here but you can't see one 1<br>Yes, male condoms here and a condom was seen 2<br>No male condoms here now 3 |
| **NO.** | **QUESTION** | **RESPONSE** |

| | | | | | | |
|---|---|---|---|---|---|---|
| B22 A-J | In the last 12 months how often have there been HIV prevention activities at this venue? Has there been any…..<br><br>READ LIST.<br><br>CIRCLE ONE RESPONSE (1, 2, 3 OR 4) FOR EACH QUESTION, A-J. | | Never | Rarely | Monthly | Weekly |
| | | **A.** HIV/AIDS prevention? | 1 | 2 | 3 | 4 |
| | | **B.** Free distribution of male condoms? | 1 | 2 | 3 | 4 |
| | | **C.** Free distribution of lube? | 1 | 2 | 3 | 4 |
| | | **D.** Free distribution of female condoms? | 1 | 2 | 3 | 4 |
| | | **E.** Male condoms for sale at venue? | 1 | 2 | 3 | 4 |
| | | **F.** Persons tested onsite for HIV? | 1 | 2 | 3 | 4 |
| | | **G.** Safer sex education by peer workers? | 1 | 2 | 3 | 4 |
| | | **H.** Visits by peer outreach workers? | 1 | 2 | 3 | 4 |
| | | **I.** Visits by a mobile clinic? | 1 | 2 | 3 | 4 |
| | | **J.** Needle exchange programme? | 1 | 2 | 3 | 4 |
| | | **K.** Other prevention activities? | 1 | 2 | 3 | 4 |

| | | | |
|---|---|---|---|
| B23 | I would like to know how law enforcement is in this location. Would you say it is good, fair, abusive, or that the police do not come here? | Good | 1 |
| | | Fair | 2 |
| | | Abusive | 3 |
| | | Police do not come here | 4 |

| | | | |
|---|---|---|---|
| B24 | Now I have a few questions about you. How do you describe yourself?<br><br>CIRCLE ONE BEST ANSWER. | Heterosexual male | 1 |
| | | Heterosexual female | 2 |
| | | Gay male | 3 |
| | | Bisexual male | 4 |
| | | Lesbian | 5 |
| | | Straight acting male, but have sex with other men | 6 |
| | | Trans woman (male to female) | 7 |
| | | Trans male (female to male) | 8 |
| | | OTHER SPECIFY:________________________________ | |
| **NO.** | **QUESTION** | **RESPONSE** | |

| | | |
|---|---|---|
| B25 | In the past 12 months have you… | Yes No<br>**A.** Sold sex for money? 1 2<br>**B.** Sold sex for goods or favours? 1 2<br>**C.** Injected drugs without a prescription? 1 2<br>MEN ONLY: Had sex with another man? 1 2 |
| B26 | INTERVIEWER OPINION:<br>HOW KNOWLEDGEABLE IS THIS RESPONDENT LIKELY TO BE ABOUT THE ACTIVITIES AND PATRONS AT THE SITE OR EVENT? | Extremely knowledgeable 1<br>Knowledgeable 2<br>Not very knowledgeable 3 |
| **IF RESPONDENT ANSWERED "NO" TO ALL QUESTIONS IN B25, THANK THE RESPONDENT FOR PARTICIPATING AND END THE SURVEY.** | | |
| B27 | IF RESPONDENT ANSWERED "YES" TO ANY QUESTION IN B25 CONTINUE TO PART III OF SURVEY. | SHOULD YOU COMPLETE PART III?:<br>Yes No<br>1 2 |
| B28 | IF RESPONDENT ANSWERED "YES" TO ANY QUESTION IN B22 BUT DID NOT COMPLETE PART III, PLEASE EXPLAIN WHY. | EXPLAIN: |

***APPENDIX 4. Form C Patron and Worker Interview Questionnaire***

## FORM C: Patron and Worker Study Interview

| PART I – COMPLETE BEFORE INTERVIEW | | | |
|---|---|---|---|
| **NO.** | **QUESTION** | | **RESPONSE** |
| Q1 | Date: | | Day ☐☐ Month ☐☐ Year ☐☐ |
| Q2 | Interviewer Code: | | Interviewer Code: ☐☐ |
| Q3 | Type of social mobilizer present: | | No social mobilizer present during site visit 0<br>Sex worker 1<br>MSM 2<br>Trans woman (Male to female) 3<br>Trans man: (Female to male) 4<br>Person who injects drugs 5<br>Other 6 |
| Q4 A-C | Geographic Code of Site: | **A.** Province ☐☐ | **B.** District ☐☐ **C.** Zone ☐☐ |
| Q5 | Site ID Number: | | Site Number: ___________________________________ |
| | Site Name Given by Community Informant:<br>IF WEBSITE, INDICATE EXACT WEBSITE. | | |
| Q6A | **C.** Verified Site Name: | | |
| | Site Address Given by Community Informant: | | |
| Q6B | **D.** Verified Site Address: | | |
| | Landmark Given by Community Informant: | | |
| Q6C | **C.** Correct Landmark: | | |

| | | |
|---|---|---|
| Q7<br>A-C | Was the site found?<br><br>IF "OTHER" IS SELECTED FOR PART A, EXPLAIN.<br><br>IF SITE IS A DUPLICATE LOCATION (FOR EXAMPLE SITE WAS ALREADY VERIFIED UNDER A DIFFERENT NAME), WRITE THE NUMBER FOR THE OTHER SITE THAT THIS SITE DUPLICATES IN Q7C. | **A.** Location not found 0<br>Location found, interview conducted 1<br>Location closed 2<br>Location found, informant declined 3<br>Not a duplicate location, but other 4<br>Duplicate location 5<br>**B.** IF OTHER (Q7A = 4), EXPLAIN:______________________________<br>**C.** IF DUPLICATE (Q7A = 5), OTHER SITE NUMBER:______________ |

| PART I – CONTINUED | | |
|---|---|---|
| **NO.** | **QUESTION** | **RESPONSE** |
| Q8<br>A-B | GPS Coordinates:<br>USE THE GPS UNIT. WRITE COORDINATES HERE.<br>FOR LONGITUDE: ALSO INDICATE WHETHER WEST OR EAST.<br>FOR LATITUDE: ALSO INDICATE WHETHER NORTH OR SOUTH. | **A.** Longitude: ___________________________<br><br>**B.** Latitude: ______________________________ |
| Q9A | Type of Site (USE CODES BELOW)<br>CODES 1 -22 ARE FOR TYPES OF VENUES.<br>CODES 23-25 ARE FOR EVENTS. IF A WEBSITE, CIRCLE 26. | ENTER 1 CODE:<br>**A.** [ ][ ] |

| | | |
|---|---|---|
| Formal bar 1 | Other street site 11 | Campus/School 21 |
| Informal bar 2 | Beach/Creek 12 | Tourist attraction 22 |
| Night/Exotic club 3 | Park/Plaza 13 | |
| Massage parlor 4 | Construction site 14 | OTHER: |
| Brothel 5 | Kiosk 15 | Cultural event 23 |
| Truck stop 6 | Store 16 | Party event 24 |
| Hotel 7 | Market 17 | Other event 25 |
| Small guest house 8 | Shopping Centre/Mall 18 | Website 26 |
| Street sex worker site 9 | Fast food/Restaurant 19 | Escort service 27 |
| Taxi stand/Bus stop 10 | Church/Temple/Mosque 20 | Phone 28 |

| | | | | |
|---|---|---|---|---|
| Q9<br>B-I | Physical characteristics of the site:<br>CIRCLE ONE ANSWER (1 OR 2) FOR EACH CHARACTERISTIC, A-H. | | Yes | No |
| | | **B.** Electricity available | 1 | 2 |

| | | | | |
|---|---|---|---|---|
| | | **C.** Tap water available | 1 | 2 |
| | | **D.** Walls and ceiling | 1 | 2 |
| | | **E.** Inside toilet | 1 | 2 |
| | | **F.** Beds on site | 1 | 2 |
| | | **G.** Videos shown onsite | 1 | 2 |
| | | **H.** TV onsite | 1 | 2 |
| | | **I.** Bar for alcohol sales onsite | 1 | 2 |
| Q9 J-N | Evidence of HIV/AIDS prevention activities visible at the venue: CIRCLE ONE ANSWER (1 OR 2) FOR EACH PREVENTION ACTIVITY ITEM, A-E. | | Yes | No |
| | | **J.** HIV/AIDS posters displayed | 1 | 2 |
| | | **K.** Condom promotion posters | 1 | 2 |
| | | **L.** Condoms visible | 1 | 2 |
| | | **M.** Lube visible | 1 | 2 |
| | | **N.** Sterile needles/syringes visible | 1 | 2 |

**PART II – BEGIN INTERVIEW**

READ: Hello. My name is < > and I am working with < > on a study coordinated by < > that will improve HIV prevention programs. I would like to ask you some questions about this site. I can offer you this information sheet that has more information about the study. This should take about 30-40 minutes.

| NO. | QUESTION | RESPONSE |
|---|---|---|
| Q10 A-C | INSTRUCTIONS TO THE INTERVIEWER (DO NOT READ ALOUD):<br>**A.** DID YOU READ THE INFORMATION SHEET TO THE RESPONDENT?<br>**B.** DID YOU READ THE CONSENT FORM TO RESPONDENT IN LANGUAGE RESPONDENT UNDERSTANDS?<br>**C.** HAVE YOU ASKED THE RESPONDENT IF HE/SHE HAS QUESTIONS, AND HAVE YOU ANSWERED THOSE QUESTIONS? | Yes No<br>**A.** 1 2<br>**B.** 1 2<br>**C.** 1 2 |
| Q11 | Are you willing to answer the questions I will ask you? | Yes No<br>1 2 |
| Q12 | What is your age? IF RESPONDENT IS YOUNGER THAN 18, STOP INTERVIEW AND FIND ANOTHER. | WRITE AGE IN YEARS: [ ][ ] |
| Q13 | Do you work here? | Yes No<br>1 2 |

| | | |
|---|---|---|
| Q14 | Were you born as a male or female? | MALE 1<br>FEMALE 2 |
| Q15 | Do you currently identify more as a male or more as a female? | MALE 1<br>FEMALE 2 |

| SECTION C -CONTINUE INTERVIEW IF RESPONDENT IS A MEMBER OF A KEY POPULATION | | |
|---|---|---|
| **NO.** | **QUESTION** | **RESPONSE** |
| C1 | In the past 7 days, on which days did you come here?<br>START WITH TODAY AND THEN GO BACK DAY BY DAY FOR THE PREVIOUS 6 DAYS. FOR EXAMPLE IF TODAY IS WEDNESDAY, CIRCLE YES FOR WEDNESDAY AND THEN ASK ABOUT TUESDAY, THEN MONDAY, ETC.<br>WHEN YOU ARE FINISHED, EVERY DAY SHOULD HAVE A YES OR NO ANSWER. | Yes No<br>Saturday 1 2<br>Friday 1 2<br>Thursday 1 2<br>Wednesday 1 2<br>Tuesday 1 2<br>Monday 1 2<br>Sunday 1 2 |
| C2 | In the past 4 weeks, how many times did you come here including today? | NUMBER:_______ |
| C3 | In the past 12 months, have you met a new sexual partner here? | Yes No<br>1 2 |
| C4 | Besides this place, how many other public places have you visited or plan to visit today to socialize, drink alcohol, or look for a person to have sex with? | NUMBER:_______ |
| C5 | Have you ever had sex? | Yes No<br>1 2 |
| C6 | Now I would like to ask about how many men and women you had sex with in the last 4 weeks, including people you had sex with once and people you had sex with more often. In total, how many men did you have sex with in the last 4 weeks? | MEN:______ |
| C7 | In total, how many women did you have sex with in the last 4 weeks? | WOMEN:______ |

| | | |
|---|---|---|
| C8 | Have you had sex with a person in the past 12 months who you never had sex with before? That is, you had sex with him or her the first time in the past 12 months. | Yes No<br>1 2 |
| C9 | Have you had sex with more than 10 people in the past 12 months? | Yes No<br>1 2 |
| C10 | Have you had anal sex in the past 12 months with a man? | Yes No<br>1 2 |
| C10A | Did you use a condom use with your last male partner? | Yes No<br>1 2 |
| **NO.** | **QUESTION** | **RESPONSE** |
| C11 | Have you received money in exchange for sex in the past 12 months? | Yes No<br>1 2 |
| C11A | Did you use a condom use with your last client? | Yes No<br>1 2 |
| C12 | Have you ever received money in exchange for sex?<br>IF NO: GO TO C14. | Yes No<br>1 2 |
| C13 | How old were you the first time you received money in exchange for sex?<br>LEAVE BLANK IF NOT APPLICABLE. | WRITE AGE: ______ |
| C14 | In the last 12 months, have you given someone money to have sex? | Yes No<br>1 2 |
| QUESTIONS ABOUT ACCESS TO SERVICES | | |
| C15 | If you wanted a condom, would it be easy to get one? | Yes No<br>1 2 |
| C16 | In the past 12 months, has an outreach worker such as a peer educator given you a condom? | Yes No |

| | | |
|---|---|---|
| | | 1 2 |
| C17 | Do you have a condom with you now? | Yes No<br>1 2 |
| C18A | In the last 12 months, have you had any burning when you pee, or discharge from your penis or vagina or anus, or sores on your genitals? | Yes No<br>1 2 |
| C18B | IF YES: In the last 12 months, have you sought care for any of these symptoms at a public or private clinic?<br>IF NOT APPLICABLE (C18=2) THEN CIRCLE 9 (NA). | Yes No NA<br>1 2 9 |
| C18C | In the past 12 months Have you been discriminated against by a healthcare worker? | Yes No NA<br>1 2 9 |
| C18D | At what type of facility did you seek care? | dedicated to HIV<br>general health care<br>outreach services<br>referral facility |
| C19 | IF RESPONDENT IS MALE, ASK: Are you circumcised?<br>IF FEMALE, CIRCLE 9 (NA). | Yes No NA<br>1 2 9 |
| C20 | How many servings of alcohol have you had in the past 24 hours? A serving is a bottle of beer or a shot of alcohol. | NUMBER:___ |
| C21 | In the past 12 months, have you received information about HIV or AIDS from an outreach worker at this site? | Yes No<br>1 2 |
| **NO.** | **QUESTION** | **RESPONSE** |
| C22 | In the past 12 months, have you received information about HIV or AIDS from the radio? | Yes No<br>1 2 |

| | | |
|---|---|---|
| C23 | In the past 12 months, have you received information about HIV or AIDS from any health worker? | Yes No<br>1 2 |
| C24 | Are you currently seeing a health worker for an HIV infection? | Yes No<br>1 2 |
| C25 | Are you currently taking antiretroviral (ART) drugs to treat an HIV infection? | Yes No<br>1 2 |
| C26 | Now I have a few questions about HIV tests. Have you ever had an HIV test?<br>IF NO: GO TO C31. | Yes No<br>1 2 |
| C27 | Has a medical provider ever told you that you are infected with HIV, based on an HIV test result? IF NO GO TO C29. | Yes No<br>1 2 |
| C28 | Think about the first time a medical provider told you that you that you were infected with HIV. Did this occur in the past 3 months? Past 12 months? Or longer ago? IF LONGER AGO, SKIP TO C31. | Past 3 Months 1<br>Past 4-12 months 2<br>Longer ago 3 |
| C29 | Have you been tested for HIV in the past 12 months?<br>IF NO, GO TO C31. | Yes No<br>1 2 |
| C30 | IF YES (C29=1) ASK: The last time you were tested, did you get your test result? | Yes No<br>1 2 |
| C31 | Some people have tried injecting drugs using a needle and syringe. Have you injected any non-prescription drug in the past 12 months? | Yes No<br>1 2 |
| C32 | In the past 12 months, have you shared a needle with another person who was injecting? | Yes No<br>1 2 |
| C33 | Do you currently get health or other services from programs designed for people who inject drugs? | Yes No<br>1 2 |
| **NO.** | **QUESTION** | **RESPONSE** |

| QUESTIONS TO ASSESS SOCIAL AND ECONOMIC VULNERABILITY | | |
|---|---|---|
| C34 | In the past 12 months, have you ever spent a night in jail or prison? | Yes No<br>1 2 |
| C35 | In the past 12 months, have you been hurt physically by the police? | Yes No<br>1 2 |
| C36 | In the past 12 months, have you used legal services? | Yes No<br>1 2 |
| C37 | Have you lived in this area less than a year? | Yes No<br>1 2 |
| C38 | Are you currently married or living with a sexual partner? | Yes No<br>1 2 |
| C39 | Did you complete secondary school? | Yes No<br>1 2 |
| END OF SURVEY. THANK THE RESPONDENT FOR PARTICIPATING! | | |
| Checked by supervisor (signature)__________________ Date: | | |

***Appendix 5. Cabinda Study Questionnaire***

***Appendix 6. RDS Non-response Assessment Form***

# RDS Non-response Assessment Form

**(This form will be translated and back-translated by different translators to Portuguese.)**

## BIOBEHAVIORAL SURVEY IN CABINDA

**Instructions: Collect this information face-to-face from returning recruiters each time they come to collect their compensation.**

**Oral consent script: I am going to ask you a few questions about the people you tried to recruit with your coupons for this survey. We ask because we are especially interested in knowing about people who say they do not want a coupon. I do not want to know anyone's name, not even yours, for these questions. May I continue?**

Coupon Identification Number: |___|___|___|___|___|___|___|___|___|___|

Name of Interviewer: ____________________________________ Date of Interview: ____________________

1. Is this the first time you have been here to collect compensation? Yes______ No______

If yes, continue. If no, answer questions for the period of time between when the subject was last here and filled out this same questionnaire and now.

2. How many coupons did you give out? ______ (Between the last time you came here to receive compensation and now).

3. How many people refused to accept coupons? _______ (if zero, skip to question 5. If > zero, continue)

4. These questions are asked for each individual who refused to accept the coupon.

| | Person 1 | Person 2 | Person 3 | Person 4 |
|---|---|---|---|---|
| 1. What is your relationship to this person (Check all that apply) | ☐ 1. A stranger, someone you met for the first time<br><br>☐ 2. Someone you knew, but not closely<br><br>☐ 3. A close friend, someone you knew very well<br><br>☐ 4. A sexual partner<br><br>☐ 5. A family member<br><br>☐ 6. A dealer<br><br>☐ 7. Other ___________ | ☐ 1. A stranger, someone you met for the first time<br><br>☐ 2. Someone you knew, but not closely<br><br>☐ 3. A close friend, someone you knew very well<br><br>☐ 4. A sexual partner<br><br>☐ 5. A family member<br><br>☐ 6. A dealer<br><br>☐ 7. Other ___________ | ☐ 1. A stranger, someone you met for the first time<br><br>☐ 2. Someone you knew, but not closely<br><br>☐ 3. A close friend, someone you knew very well<br><br>☐ 4. A sexual partner<br><br>☐ 5. A family member<br><br>☐ 6. A dealer<br><br>☐ 7. Other ___________ | ☐ 1. A stranger, someone you met for the first time<br><br>☐ 2. Someone you knew, but not closely<br><br>☐ 3. A close friend, someone you knew very well<br><br>☐ 4. A sexual partner<br><br>☐ 5. A family member<br><br>☐ 6. A dealer<br><br>☐ 7. Other ___________ |
| 2. How long have you known this person? | ☐ 1. Less than 6 months<br><br>☐ 2. 6 months to 1 year<br><br>☐ 3. 1-2 years<br><br>☐ 4. 3-6 years<br><br>☐ 5. More than 6 years | ☐ 1. Less than 6 months<br><br>☐ 2. 6 months to 1 year<br><br>☐ 3. 1-2 years<br><br>☐ 4. 3-6 years<br><br>☐ 5. More than 6 years | ☐ 1. Less than 6 months<br><br>☐ 2. 6 months to 1 year<br><br>☐ 3. 1-2 years<br><br>☐ 4. 3-6 years<br><br>☐ 5. More than 6 years | ☐ 1. Less than 6 months<br><br>☐ 2. 6 months to 1 year<br><br>☐ 3. 1-2 years<br><br>☐ 4. 3-6 years<br><br>☐ 5. More than 6 years |
| 3. What was the main reason given for refusing to accept a coupon?<br><br>(Do not read. Ask for each individual who refused to accept the coupon)<br><br>**Check all that apply.** | ☐ 1. Too busy<br><br>☐ 2. Already had a coupon<br><br>☐ 3. Already participated in survey<br><br>☐ 4. Younger than 18<br><br>☐ 6. Not resident of _________<br><br>☐ 7. Fear of being identified as _____<br>☐ 8. Site is too far away | ☐ 1. Too busy<br><br>☐ 2. Already had a coupon<br><br>☐ 3. Already participated in survey<br><br>☐ 4. Younger than 18<br><br>☐ 6. Not resident of _________<br><br>☐ 7. Fear of being identified as _____<br>☐ 8. Site is too far away | ☐ 1. Too busy<br><br>☐ 2. Already had a coupon<br><br>☐ 3. Already participated in survey<br><br>☐ 4. Younger than 18<br><br>☐ 6. Not resident of _________<br><br>☐ 7. Fear of being identified as _____<br>☐ 8. Site is too far away | ☐ 1. Too busy<br><br>☐ 2. Already had a coupon<br><br>☐ 3. Already participated in survey<br><br>☐ 4. Younger than 18<br><br>☐ 6. Not resident of _________<br><br>☐ 7. Fear of being identified as _____<br>☐ 8. Site is too far away |

| | | | | |
|---|---|---|---|---|
| | ☐ 9. Not interested<br><br>☐ 10. Incentive is not worth the time<br><br>☐ 11. Other ___________ | ☐ 9. Not interested<br><br>☐ 10. Incentive is not worth the time<br><br>☐ 11. Other ___________ | ☐ 9. Not interested<br><br>☐ 10. Incentive is not worth the time<br><br>☐ 11. Other ___________ | ☐ 9. Not interested<br><br>☐ 10. Incentive is not worth the time<br><br>☐ 11. Other ___________ |

5. Of those people who accepted a coupon:

| | **Person 1** | **Person 2** | **Person 3** |
|---|---|---|---|
| 1. What is your relationship to this person (Check all that apply) | ☐ 1. A stranger, someone you met for the first time<br><br>☐ 2. Someone you knew, but not closely<br><br>☐ 3. A close friend, someone you knew very well<br><br>☐ 4. A sexual partner<br><br>☐ 5. A family member<br><br>☐ 6. A dealer<br><br>☐ 7. Other ____________ | ☐ 1. A stranger, someone you met for the first time<br><br>☐ 2. Someone you knew, but not closely<br><br>☐ 3. A close friend, someone you knew very well<br><br>☐ 4. A sexual partner<br><br>☐ 5. A family member<br><br>☐ 6. A dealer<br><br>☐ 7. Other ____________ | ☐ 1. A stranger, someone you met for the first time<br><br>☐ 2. Someone you knew, but not closely<br><br>☐ 3. A close friend, someone you knew very well<br><br>☐ 4. A sexual partner<br><br>☐ 5. A family member<br><br>☐ 6. A dealer<br><br>☐ 7. Other ____________ |
| 2. How long have you known this person? | ☐ 1. Less than 6 months<br><br>☐ 2. 6 months to 1 year<br><br>☐ 3. 1-2 years<br><br>☐ 4. 3-6 years<br><br>☐ 5. More than 6 years | ☐ 1. Less than 6 months<br><br>☐ 2. 6 months to 1 year<br><br>☐ 3. 1-2 years<br><br>☐ 4. 3-6 years<br><br>☐ 5. More than 6 years | ☐ 1. Less than 6 months<br><br>☐ 2. 6 months to 1 year<br><br>☐ 3. 1-2 years<br><br>☐ 4. 3-6 years<br><br>☐ 5. More than 6 years |
| 3. Did this person know your recruiter, that is, the person who gave you your coupon? | ☐ 1. Yes<br><br>☐ 2. No<br><br>☐ 3. Don't know | ☐ 1. Yes<br><br>☐ 2. No<br><br>☐ 3. Don't know | ☐ 1. Yes<br><br>☐ 2. No<br><br>☐ 3. Don't know |

6. How many coupons have <u>you</u> refused to accept? _________

## Consent Form for Mapping Readiness Assessment: In Depth Interviews and Focus Groups

**IRB Study #**_____15-1154________________
**Consent Form Version Date:** ___7/21/2015_________
**Angola Study Contact:** Pedro Sapalalo
**Angola Study Contact telephone number:** 924 123 312
**Angola Study Contact email:** tchikos@gmail.com

**Hello Mrs,Mr!**

### Who is conducting this study?

Tchikos Consultancy (Consultoria Empresarial), in collaboration with the National Institute Against HIV/AIDS (INLS) of the Ministry of Public Health of Angola, is conducting a Mapping Readiness study in 4 Provinces of Angola with USAID support.

### What is this study about?

The study is part of an outreach program to populations at risk of health problems such as infectious diseases especially HIV. This survey has been approved by the Ministry of Public Health and INLS in Angola. We will ask you a few questions to get some information necessary to protect the safety, well-being and confidentiality of individuals who participate in this type of study and to develop and monitor the programs to prevent new HIV cases. The new knowledge obtained from the study will help to identify where better HIV/AIDS programs are needed in this area.

### Why is this study important?

The results will be used to improve HIV programs in Angola.

### What will happen if I participate?

If you decide to take part in this project, you will be interviewed either in a group or alone. If you are interviewed in a group, you will sit in a room with 6-10 other individuals and two project members and will be asked a series of questions for about one hour. Your discussion will be audio-recorded. If you are in a one-on-one interview, you will sit in a room with two project leaders and will be asked questions for about one hour.

### What are the benefits to me for participating?

Your responses will also be used to help improve HIV prevention programs in Angola.

### What are the risks to me for participating?

Some of the questions we ask you may cause you to feel embarrassment and/or emotional distress. You can choose not to answer any of the questions we ask you. All responses will remain confidential.

**Can I refuse?**

Participation is voluntary. You have the right to refuse to participate, or you can refuse to answer any question. If you change your mind about participating during the study, you have the right to withdraw and end your participation at any time.

**Who will have access to my answers?**

Your answers will not be shared outside of the team working on this study. We will not ask or record your name or other information about your identity. The questionnaires will be kept in a locked cabinet. When describing the findings from the study, only summary information will be used and never any information about you specifically. In the focus group, no questions will be directed to you individually, but instead will be posed to the group. You may choose to respond or not respond at any point during the discussion. The focus group discussion will be audiotaped so we can capture comments in a transcript for analysis.

**What if I have questions?**

The study is being conducted by Tchikos Consultoria Empresarial in collaboration with the National Institute Against HIV/AIDS (INLS) of the Ministry of Public Health, with support and oversight the University of North Carolina.

If you have any questions about the study or about your participation, you may contact the local study coordinator Mr. Pedro Sapalalo at 924 123 312 or 917 013 371. If he is unavailable, you may also contact Mr. William Miller, the UNC study coordinator at 938 207 292 or +49 176 5441 2859.

I was informed about the nature and the contents of the study on the mapping readiness and HIV prevention in Angola.

I was given the opportunity to ask questions about the study and obtained satisfactory answers. Accordingly, I agree freely to participate in the study and I know that I can withdraw at any time without prejudice or explanation. By completing this survey, I agree to collaborate completely and to give my modest contribution to the HIV prevention in Angola. My information will be handled confidentially.

| | |
|---|---|
| Initials or "X" of the interviewed | Signature of the investigator obtaining consent |
| ---------------------------------- | ---------------------------------- |

Date and time: ____/____/2015 at ____hrs ___min

*APPENDIX 8. Fact sheet for Community and Venue Informant Interviews (in lieu of consent form)*

# FACT SHEET FOR PROGRAMMATIC MAPPING:

# PLACE STUDY IN ANGOLA

## What is this survey about?

The purpose of the study is to identify ways to target HIV prevention programs to prevent more people from becoming infected with HIV. This survey has been approved by the National Institute of Public Health's, Research and Ethics Committee. We will ask you a few questions to get some information necessary to develop and monitor the programs.

## What will the survey cover?

The survey will include questions about your knowledge of this particular area or location. Some questions may be related to sexual partnerships, but none of the questions will be about your behavior specifically.

The interview will last between 10 to 30 minutes of your time.

## Can I refuse?

Participation is voluntary and you can quit at any time. You have the right to refuse to participate or answer any questions that you feel uncomfortable with. If you change your mind about participating during the course of the interview, you have the right to withdraw at any time.

## Who will see my survey answers?

The only people who will see the results are people working on this study. The data will be kept on a secure server and password locked smartphones. Your name will not appear anywhere on this survey. We will not ask your name. When describing the findings from the survey only summary information will be used and never any information about you specifically.

## Who wants to know?

The study is being conducted by Tchikos Consultancy in connection with the National Institute against HIV/AIDS (INLS). The new knowledge obtained from the study will help identify where better HIV programs are needed in this area in order to prevent the spread of infections.

If you have any questions about the study or your rights as a research participant, you can contact Mr. Pedro Sapalalo, Tchikos Consultoria Empresarial. He can be reached at Tel: 924 123 312.

You can also contact Mr. William Miller of the University of North Carolina at 938 207 292 or +49 176 5441 2859.

## For Your Information: Health Facilities You Can Call or Visit for Treatment and Care Services for HIV

<To be filled in separately for each district.>

**HIV PREVALENCE STUDY**

## Consent Form for Form C

**IRB Study #**_____15-1154________________
**Consent Form Version Date:** ___6/29/2015_________
**Angola Study Contact:** Pedro Sapalalo
**Angola Study Contact telephone number:** 924 123 312
**Angola Study Contact email:** tchikos@gmail.com

**Hello Mrs,Mr!**

### Who is conducting this study?

Tchikos Consultancy (Consultoria Empresarial), in collaboration with the National Institute Against HIV/AIDS (INLS) of the Ministry of Public Health of Angola, is conducting a survey for the implementation of Programmatic Mapping: the PLACE Method in 4 Provinces of Angola with USAID support.

### What is this study about?

The study is part of an outreach program to populations at risk of health problems such as infectious diseases especially HIV. This survey has been approved by the Ministry of Public Health and INLS in Angola. We will ask you a few questions to get some information necessary to develop and monitor the programs to prevent new HIV cases. The new knowledge obtained from the study will help to identify where better HIV/AIDS programs are needed in this area.

### Why is this study important?

The results will be used to improve HIV programs in Angola.

### What will happen if I participate?

You were approached for this study because you are present at a venue selected for a visit by the study. If you choose to participate, someone will interview you about your socio-demographic background, sexual behavior, condom use, and exposure to health services and HIV prevention programs. This interview will last about 30 minutes.

### What about HIV testing?

If you accept to participate in this survey, you will also undergo an HIV test at no cost. The testing process will take approximately 30-45 minutes. Trained personnel will prick your finger to draw a small blood sample to conduct a rapid HIV test.

You will receive pre- and post-test counseling, and you can receive your results at this site today if you choose. The confidentiality of your results is very important to us. If you choose to receive your test result and you have a positive test, we will offer you a rapid CD4 test and collect several dried blood spots for later viral load testing. We will also provide you with information about services for HIV treatment.

If, based on the interview, you are at risk of other sexually transmitted infections you will be offered testing for syphilis, gonorrhea, chlamydia, trichomoniasis and Hepatitis B. Syphilis and Hepatitis B testing will be through finger prick at the same time as HIV. For gonorrhea, chlamydia and trichomoniasis testing you will be asked to provide a urine sample, an anal or vaginal swab. In 2 weeks, you can pick up the results of the gonorrhea, chlamydia and trichomoniasis testing at the address below or over the phone. We will not contact you following your participation.

Gonorrhoea, chlamydia, trichomoniasis and viral load results can be picked up at the study laboratory: Rua das Salgas, Barrio dos Pescadores, Cacuaco, Luanda; Phone number: 924 123 312.

---

**What are the benefits to me for participating?**

You will receive an HIV test at no cost and learn your HIV status if you choose. If you have a positive test, you will have the option to receive information about where to access additional services related to HIV in addition to CD4 testing. Your CD4 test result will help determine when you can start treatment for HIV. Your responses will also be used to help improve HIV prevention programs in Angola.

**What are the risks to me for participating?**

Some of the questions we ask you may cause you to feel embarrassment and/or emotional distress. You can choose not to answer any of the questions we ask you. It is not uncommon for people that learn they are HIV positive for the first time to experience emotional pain and discomfort. However, a trained counselor will talk to you before and after you undergo the HIV test. You can also choose not to receive your result. Results of the rapid HIV test will remain confidential.

**Can I refuse?**

Participation is voluntary. You have the right to refuse to participate, or you can refuse to answer any question in the survey. If you change your mind about participating during the survey, you have the right to withdraw and end your participation at any time.

**Who will have access to my survey answers and test results?**

Answers from your survey and your test result will not be shared outside of the team working on this study. We will not ask or record your name or other information about your identity. The questionnaires and test results will be kept in a locked cabinet. When describing the findings from the study, only summary information will be used and never any information about you specifically.

## What if I have questions?

The study is being conducted by Tchikos Consultoria Empresarial in collaboration with the National Institute Against HIV/AIDS (INLS) of the Ministry of Public Health, with support and oversight the University of North Carolina.

If you have any questions about the study or about your participation, you may contact the local study coordinator Mr. Pedro Sapalalo at 924 123 312 or 917 013 371. If he is unavailable, you may also contact Mr. William Miller, the UNC study coordinator at 938 207 292 or +49 176 5441 2859.

---

**HIV PREVALENCE STUDY**

## Consent Form for Form C

**IRB Study #**_____15-1154________________
**Consent Form Version Date:** ___6/04/2015_________
**Angola Study Contact:** Pedro Sapalalo
**Angola Study Contact telephone number:** 924 123 312
**Angola Study Contact email:** tchikos@gmail.com

I was informed about the nature and the contents of the study on the identification of the priority areas of HIV prevention in Angola.

I was given the opportunity to ask questions about the study and obtained satisfactory answers. Accordingly, I agree freely to participate in the study and I know that I can withdraw at any time without prejudice or explanation. By completing this survey, I agree to collaborate completely and to give my modest contribution to the HIV prevention in Angola. I know that as part of my participation, I will voluntarily undergo a rapid test for HIV, and will receive the test results at the site if I want. In the case that I test positive for HIV, I will voluntarily undergo a rapid CD4 test. If my behavior puts me at risk, I may undergo testing for other sexually transmitted infections such as syphilis, gonorrhea, chlamydia, trichomoniasis and Hepatitis B. My information will be handled confidentially.

| Initials or "X" of the interviewed | Signature of the investigator obtaining consent |
|---|---|
| ---------------------------------- | ----------------------------------- |

Date and time: ____/____/2015 at ____hrs ___min

## HIV PREVALENCE STUDY

### Consent to Store Dried Blood Spots

**IRB Study #**_____15-1154________________
**Consent Form Version Date:** ___6/04/2015_________
**Angola Study Contact:** Pedro Sapalalo
**Angola Study Contact telephone number:** 924 123 312
**Angola Study Contact email:** tchikos@gmail.com

### Hello Mrs, Mr!

### What is this study about?

We would like to store additional dried blood spots that are left over after completing the tests associated with the survey. Dried blood spots would be stored at the National Institute Against HIV/AIDS (INLS) for future analysis of HIV genotype and sexually transmitted infections. The stored samples will not include any personal information. The samples will be labelled with a number. Any file linking the number to your name to the samples will be destroyed when the study is completed. The samples will be stored for up to five years after which they will be destroyed.

### Why is this study important?

The samples can be used to gain better understanding of infectious diseases including the types of the HIV virus that exist in Angola.

### Can I refuse?

You can choose not to permit the storage of your dried blood spots. You can decide at any time that you do not want your samples stored.

### What if I have questions?

The study is being conducted by Tchikos Consultoria Empresarial in collaboration with the National Institute Against HIV/AIDS (INLS) of the Ministry of Public Health, with support and oversight the University of North Carolina.

If you have any questions about the study or about your participation, you may contact the local study coordinator Mr. Pedro Sapalalo at 924 123 312 or 917 013 371. If he is unavailable, you may also contact Mr. William Miller, the UNC study coordinator at 938 207 292 or +49 176 5441 2859.

## HIV PREVALENCE STUDY

## Consent to Store Dried Blood Spots

**IRB Study #**_____15-1154________________
**Consent Form Version Date:** ___6/04/2015_________
**Angola Study Contact:** Pedro Sapalalo
**Angola Study Contact telephone number:** 924 123 312
**Angola Study Contact email:** tchikos@gmail.com

I was informed about the storage of dried blood samples for future analyses.

I was given the opportunity to ask questions about the study and obtained satisfactory answers. Accordingly, I agree freely to participate in the study and I know that I can withdraw at any time without prejudice or explanation. I know that as part of my participation, my dried blood samples will be stored at the National Institute Against HIV/AIDS for no more than 5 years. My information will be handled confidentially.

| Initials or "X" of the interviewed | Signature of the investigator obtaining consent |
| --- | --- |
| ---------------------------------- | ---------------------------------- |

Date and time: ____/____/2015 at ____hrs ___min

*APPENDIX 11. RDS Coupon Example*

Coupon #
00102

**Site:**

**Date/Hours**

**3:30 pm-7:30 pm Monday-Friday**

**8am 4 pm Saturday**

**Please keep this ticket!**

**Address:**

**Telephone:**

**ID #: _ _ _ _ _**

**Activation Date: __/__/__**

**Expiration Date: __/__/__**

Coupon #
00102

**Site**:

**Date/Hours**

3:30 pm-7:30 pm Monday-Friday

8am-4 pm Saturday

**Give this to a friend**

**Address:**

**Telephone: ???**

**ID #: _ _ _ _ _ _**

**Activation Date: __/__/__**

**Expiration Date: __/__/__**

***APPENDIX 12. Data Corrections Log***

## <u>Data Corrections Log</u>

**(This form will be translated and back-translated by different translators to Portuguese.)**

## PROGRAMMATIC MAPPING AND HIV PREVALENCE STUDY IN ANGOLA

| Interview Date | Event # | Venue ID | Interviewer ID | Survey ID | Problem | Correction | Initials |
|---|---|---|---|---|---|---|---|
| | | | | | | | |
| | | | | | | | |
| | | | | | | | |
| | | | | | | | |
| | | | | | | | |
| | | | | | | | |
| | | | | | | | |
| | | | | | | | |

*APPENDIX 13. Population Size Estimation Contact Log*

Population Size Estimation Contact Form Page _________ of __________

***(This form will be translated and back-translated by different translators to Portuguese.)***

**PROGRAMMATIC MAPPING AND HIV PREVALENCE STUDY IN ANGOLA**

| | | | | | |
|---|---|---|---|---|---|
| 1. Date | ____/____/09 | ____/____/09 | ____/____/09 | ____/____/09 | ____/____/09 |
| 2. Distribution site | | | | | |
| 3. Time of distribution | | | | | |
| **Demographic Information:** | | | | | |
| 4. Age *(If <18 years old, do not give object.)* | | | | | |
| 5. Living in ________ *(If no, do not give object.)* | □ Yes □ No | □ Yes □ No | □ Yes □ No | □ Yes □ No | □ Yes □ No |
| 6. Is a female sex worker *(If no, do not give object.)* | □Yes □ No | □Yes □ No | □Yes □ No | □Yes □ No | □Yes □ No |
| **Unique Object Distribution:** | | | | | |
| 7. Have already received [object] (***If yes**, do not give object).* | □Yes □ No | □Yes □ No | □Yes □ No | □Yes □ No | □Yes □ No |
| 8. Accepted [object] | □Yes □ No | □Yes □ No | □Yes □ No | □Yes □ No | □Yes □ No |

**Instructions for using Size Estimation Field Staff Contact Log**

**1. Date:** Write the date of distribution activity.
**2. Distribution Site:** Write the site in which the distribution activity is taking place.
**3. Time:** Write the time of distribution activity.
**4. Age:** If the person is under 15 years old, do not give object or collect any other information.
**5. Living or working in Cabinda Province:** Ask question: *Do you live or work in Cabinda province?* If answer is no, do not give object or collect any other information.
**6. Is a female sex worker:** Ask question: *In the past 12 months, have you had received money for sex?* If answer is no, do not give object or collect any other information.
**7. Have already received [object]:** Ask question: *Have you received a [object] by a field staff member in the past two weeks?*
**8. Accepted [object]:** Explain that these objects are being distributed for a survey and that the only participation needed from him in this survey is to accept the object and that no other questions will be asked. If the person accepts the object, ask him to keep the object because in about two weeks to four months, someone might ask them again if they have received this object.

***Appendix 14. References***


1. Ministério da Saúde (MINSA), Instituto Nacional de Luta contra a SIDA (INLS). V Plano Estratégico Nacional de Resposta às ITS-VIH/SIDA e Hepatites Virais 2015 - 2018: " Zero é a nossa Meta". Luanda, Angola: MINSA, INLS; 2014.
2. Kendall C, Kerr LR, Mota RM, et al. Population size, HIV, and behavior among MSM in Luanda, Angola: challenges and findings in the first ever HIV and syphilis biological and behavioral survey. J Acquir Immune Defic Syndr 2014;66:544-51.
3. Pinho A, Bastos FI, Sampaio CAM, et al. Estudo de Vigilância Comportamental e Serológica do VIH e Sífilis (BSS) entre mulheres jovens envolvidas em Sexo Transaccional na zona da Fronteira entre Angola e Namíbia. Luanda, Angola: Instituto Nacional de Saude Publica de Angola; Instituto Nacional da Luta contra Sida (INLS); FIOCRUZ; CDC 2011.
4. Ministerio da Saúde - Instituto Nacional de Luta Contra a Sida (INLS). Relatório de Progresso da Resposta Global à SIDA (GARPR, 2014) República de Angola. Luanda2014.
5. Weir SS, Pailman C, Mahlalela X, Coetzee N, Meidany F, Boerma JT. From people to places: focusing AIDS prevention efforts where it matters most. AIDS 2003;17:895-903.
6. Weir SS, Tate JE, Zhusupov B, Boerma JT. Where the action is: monitoring local trends in sexual behaviour. Sex Transm Infect 2004;80 Suppl 2:ii63-8.
7. Measure Evaluation Project. Priorities for Local AIDS Control Efforts: a manual for implementing the PLACE method. Chapel Hill: MEASURE Evaluation: University of North Carolina at Chapel Hill; 2005.
8. White RG, Hakim AJ, Salganik MJ, et al. Strengthening the Reporting of Observational Studies in Epidemiology for respondent-driven sampling studies: "STROBE-RDS" statement. Journal of clinical epidemiology 2015.
9. Erickson B. Some problems of inference from chain data. Sociological Methodology Research 1971;10:276-302.
10. Ramirez-Valles J, Heckathorn DD, Vazquez R, Diaz RM, Campbell RT. From networks to populations: the development and application of respondent-driven sampling among IDUs and Latino gay men. AIDS Behav 2005;9:387-402.
11. Johnston LG, Khanam R, Reza M, et al. The effectiveness of respondent driven sampling for recruiting males who have sex with males in Dhaka, Bangladesh. AIDS Behav 2008;12:294-304.
12. Heckathorn D. Respondent driven sampling: A new approach to the study of hidden populations. Social Problems 1997;44:174-99.
13. Csete J, Cohen J. Health benefits of legal services for criminalized populations: the case of people who use drugs, sex workers and sexual and gender minorities. Journal of Law, Medicine and Ethics 2010;38:816-31.
