# Supplementary material for: Achieving the first 90 for key populations in sub‐Saharan Africa through venue‐based outreach: challenges and opportunities for HIV prevention based on PLACE study findings from Malawi and Angola
Source: J Int AIDS Soc. 2018 Jul 22;21(Suppl Suppl 5):e25132. doi: 10.1002/jia2.25132 (PMC6055127; doi:10.1002/jia2.25132)
Supplement: Supplementary file 2 — Additional file 2: Table S1. Demographic and behavioural characteristics of women identifying as FSWs and women reporting receiving money for sex in the last six months. [file JIA2-21-e25132-s002.doc]

**Supplementary Table 1. Demographic and behavioural characteristics of women identifying as FSWs and women reporting receiving money for sex in the last 6 months.**

| **Characteristic** | **Population Prevalence (95% CI)**^†^ | | |
| --- | --- | --- | --- |
|  | **All FSW**  **(n=959)** | **Identifies as FSW (n=704)** | **Received Money for Sex, Last 6 Months**  **(n=916)** |
| **Age in years, median (IQR)** | 26 (21–31) | 26 (21–31) | 26 (21–31) |
| **Completed secondary school** | 34% | 31% (26, 37) | 34% (29, 40) |
| **Employed full- or part-time** | 49% | 50% (43, 58) | 49% (43, 56) |
| **Works at the venue** | 58% | 63% (56, 71) | 58% (52, 66) |
| **Number new sex partners, past 4 weeks, median (IQR)** | 2 (1–5) | 2 (1–5) | 2 (1–5) |
| **Identifies as FSW** | 78% | 100% | 77% (70, 85) |
| **Received money for sex, last 6 months** | 95% | 94% (91, 97) | 100% |
| †Population prevalence is weighted based on venue-based sampling strategy.  CI: Confidence interval; FSW: Female sex worker; IQR: Interquartile range | | | |
